# MR-TADF liquid crystals: towards self assembling host–guest mixtures showing narrowband emission from the mesophase†‡

**DOI:** 10.1039/d4sc04429k

**Published:** 2024-10-11

**Authors:** Julius A. Knöller, Franziska Müller, Tomas Matulaitis, John M. dos Santos, Abhishek Kumar Gupta, Eli Zysman-Colman, Sabine Laschat

**Affiliations:** a Institute of Organic Chemistry, University of Stuttgart Pfaffenwaldring 55 D-70569 Stuttgart Germany sabine.laschat@oc.uni-stuttgart.de; b Organic Semiconductor Centre, EaStCHEM School of Chemistry, University of St Andrews St Andrews, Fife KY16 9ST UK eli.zysman-colman@st-andrews.ac.uk +44 (0)1334 463808 +44 (0)1334 463826

## Abstract

Creating (room temperature) liquid crystalline TADF materials that retain the photophysical properties of the monomolecular TADF emitters remains a formidable challenge. The strong intramolecular interactions required for formation of a liquid crystal usually adversely affect the photophysical properties and balancing them is not yet possible. In this work, we present a novel host–guest approach enabling unperturbed, narrowband emission from an MR-TADF emissive core from strongly aggregated columnar hexagonal (Col_h_) liquid crystals. By modifying the DOBNA scaffold with mesogenic groups bearing alkoxy chains of different lengths, we created a library of Col_h_ liquid crystals featuring phase ranges >100 K and room temperature mesomorphism. Expectedly, these exhibit broad excimer emission from their neat films, so we exploited their high singlet (S_1_ ∼2.9 eV) and triplet (T_1_ ∼2.5 eV) energies by doping them with the MR-TADF guest BCzBN. Upon excitation of the host, efficient Förster Resonance Energy Transfer (FRET) resulted in almost exclusive emission from BCzBN. The ability of the liquid crystallinity of the host to not be adversely affected by the presence of BCzBN is demonstrated as is the localization of the guest molecules within the aliphatic chain network of the host, resulting in extremely narrowband emission (FWHM = 14–15 nm). With this work we demonstrate a strategy for the self-assembly of materials with previously mutually incompatible properties in emissive liquid crystalline systems: strong aggregation in Col_h_ mesophases, and narrowband emission from a MR-TADF core.

## Abstract 2

We developed a MR-TADF based, columnar liquid crystalline host system and doped it with a MR-TADF guest. Efficient FRET allowed us to construct the first columnar liquid crystal with narrowband MR-TADF from the strongly aggregated mesophase.

## Graphical abstract

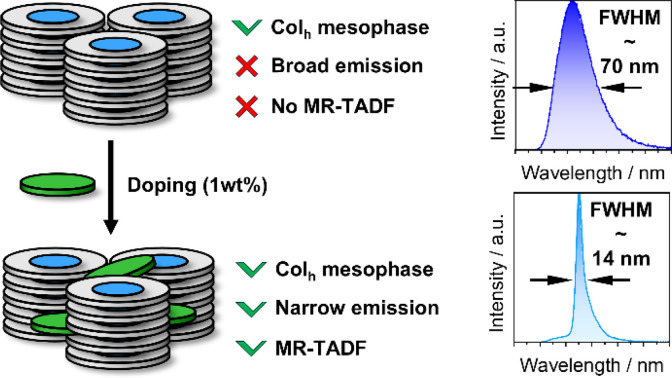

## Introduction

Thermally activated delayed fluorescence (TADF), which involves the (up)conversion of triplet excitons (T_1_) to singlets (S_1_), can be induced in an organic fluorophore through one of two principal distinct molecular designs. First, and more common, are compounds that adopt a strongly twisted donor–acceptor structure (DA-TADF).^1–5^ An exciting alternative strategy involves the doping of a polycyclic aromatic hydrocarbon compound with electron-poor atoms/units (B, C=O, P=O) and electron-rich atoms (O, N, S, Se) possessing antagonistic mesomeric effects,^6–9^ embodied in so-called multiresonant TADF (MR-TADF) emitters.^10,11^ MR-TADF materials are highly sought after as they show narrowband emission and high photoluminescence quantum yields (*Φ*_PL_) resulting from their rigid structure.^6–8^ Although being employed in many fields from time-resolved luminescence imaging reagents,^12–14^ as photosensitizers for solar fuels^14,15^ or as photocatalysts for organic transformations,^16,17^ TADF compounds are most prominently used as state-of-the-art emitters in organic light emitting diodes (OLEDs).^1–11^

Liquid crystals (LCs) and especially columnar LCs are highly sought after for their anisotropic charge mobility and have thus been heavily investigated in the context of organic electronics.^18–22^ In this context, numerous luminescent LCs have been developed as emitter materials for OLEDs.^22–25^

**Fig. 1:**
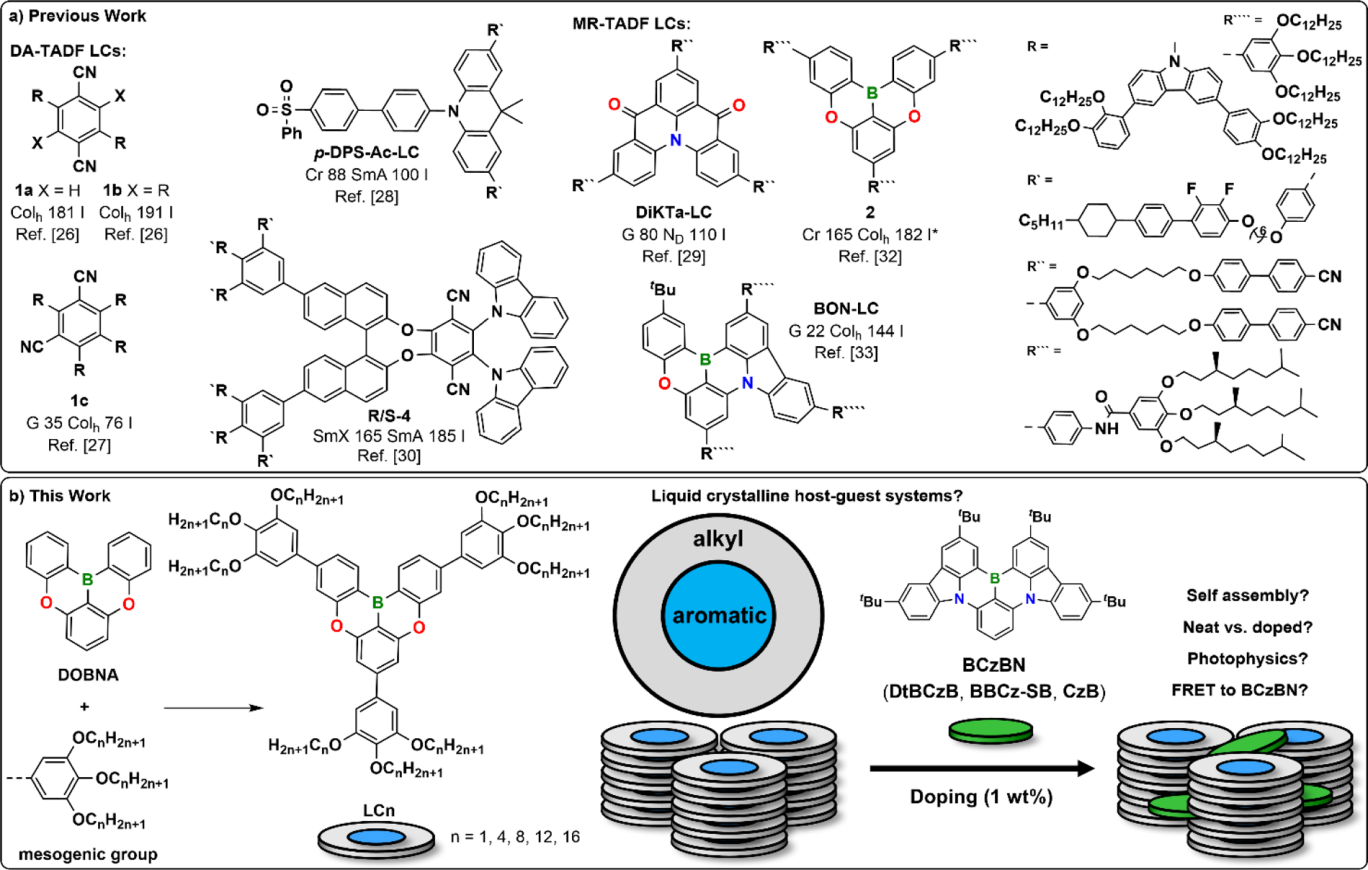
(a) Previously reported DA-TADF (1a–1c, *p*-DPS-Ac-LC, *R*/*S*-4) and MR-TADF LCs (DiKTa-LC, 2, BON-LC); (b) our strategy for development of a liquid crystalline host–guest system based on LCn doped with BCzBN.

Most recently, TADF liquid crystals (LCs) (TADF-LCs) have emerged as a new class of emitters.^26–31^ Several columnar hexagonal (Col_h_) TADF-LCs based on an archetypal carbazole-benzonitrile DA-TADF system^1^ functionalized with long alkoxy chains 1a–1c have been developed (Fig. 1).^26,27^ Furthermore, smectic derivatives *p*-DPS-Ac-LC^28^ and *R*/*S*-4 ^30^ unit have been reported.^28^ MR-TADF skeletons have been employed in the nematic discotic (*N*_D_) DiKTa-LC^29^ and boron-based Col_h_ LCs 2 ^32^ and BON-LC.^31^

The distinct self-assembly of TADF-LCs allows for increased charge mobility,^28^ improved solution processability,^26–29^ controlled alignment of their transition dipoles,^29^ and boosted circularly polarized luminescence.^30^ While the above-mentioned advantages of TADF-LCs stem from their distinct self-assembly, the strong intramolecular interactions required for LC formation adversely affect their photophysics. TADF-LCs usually display aggregation-caused quenching as well as red-shifted and broadened emission profiles as neat films.^26–31^ In other words, the desired self-assembly impairs the photophysical properties of the TADF-LCs. This is especially an issue for MR-TADF based TADF-LCs as both 2 and BON-LC display broad excimer emission without a delayed component in their mesophases.^31,32^ These effects can be partially suppressed in the low ordered, nematic discotic (*N*_D_) DiKTa-LC (Fig. 1).^29^ As a countermeasure, TADF-LCs are doped (1–20 wt%) into small molecular or polymeric host materials to suppress undesirable intramolecular interactions,^26–30,33^ just as most of their non-LC congeners.^34,35^ While this improves their photophysics, the doped systems do not show LC behaviour – undermining the initial effort to develop TADF-LCs. A columnar LC preserving the delicate photophysics of a (prone-to-excimer formation) MR-TADF emitter remains elusive to date.^36–38^

Inspired by reports demonstrating Förster Resonance Energy Transfer (FRET) from liquid crystalline hosts to guest molecules,^39–41^ we posited that a MR-TADF based host–guest system would address the outstanding issue of conserving the narrowband emission in the liquid crystalline phase.

Based on DOBNA,^10^ several high-energy hosts for MR-TADF materials such as DOBNA-Tol,^42^ have been developed.^42–47^ Thus we propose LCn, a potentially liquid crystalline host^32^ containing this MR-TADF core (Fig. 1b). Decoration of the DOBNA^10^ core with three mesogenic groups bearing alkoxy chains of different lengths (*n* = 1, 4, 8, 12, 16) was expected to induce the desired mesomorphic behaviour of LCn.^33,48^ By doping analogues of LCn with the MR-TADF emitter BCzBN (aka DtCzBN, BCz-BN, Cz-B),^38,49,50^ we demonstrate a liquid crystalline host–guest system that preserves the mesomorphic properties of the LCn host and the characteristic narrowband emissive properties of the BCzBN guest.

## Results and discussion

### Theoretical calculations

To understand the impact of the electron-rich mesogenic groups on the photophysical properties of the DOBNA core, we modelled all 5 envisioned derivatives of the LCn series using time-dependent density functional theory (TD-DFT) at the PBE0/6-31G(d,p) level with the Tamm–Dancoff-approximation (TDA). The highest occupied and lowest unoccupied molecular orbitals, HOMO and LUMO, of each of the LCn derivatives are similarly distributed across the MR-TADF cores (Fig. S2‡). While the methyl derivative LC1 has HOMO/LUMO energies of *E*_HOMO_ = −5.82 eV/*E*_LUMO_ = −1.73 eV, an increase in the alkyl chain length results in a small destabilization of these energies in LC000, with *E*_HOMO_ ranging between −5.64 and −5.66 eV while the LUMO remains essentially the same at *E*_LUMO_ = −1.60 eV (Fig. S2‡). These values are comparable to the ones reported for DOBNA (*E*_HOMO_ = −5.62 eV/*E*_LUMO_ = −1.66 eV) computed using B3LYP/6-31G(d), suggesting that the mesogenic units will only influence slightly the photophysics of the DOBNA core as monomolecular systems.^10^ Recognizing that state energies are poorly modelled using DFT,^51,52^ we modelled a representative example, LC1, using double hybrid density functional theory (DH-DFT) at the wPBEPP86/cc-pVDz level to obtain accurate S_1_ and T_1_ energies (Fig. S1‡).^31,53,54^ The difference density plots reveal the characteristic alternating pattern of increasing and decreasing electron density in the S_1_ and T_1_ states compared to the ground state, while the transition to the S_1_ state has a high oscillator strength, *f*, of 0.89. These results are typical for an S_1_ state of short-range charge transfer (SRCT) character that is emblematic of MR-TADF emitters.^10,55^ The computed S_1_ and T_1_ energies are S_1_ = 3.76 eV and T_1_ = 3.44 eV, resulting in a somewhat large singlet-triplet energy gap, Δ*E*_ST_ = 0.31 eV.

### Synthesis

**Scheme 1:**
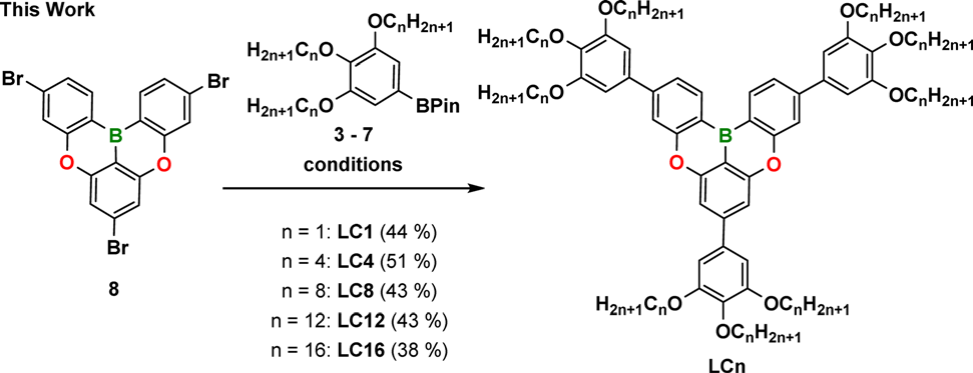
Synthetic procedure for the preparation of the library of LCn compounds possessing different chain lengths varying from –CH_3_ to C_16_H_33_. Conditions: Pd(PPh_3_)_4_ (0.15 equiv.), 3–7 (3.5 equiv.), Cs_2_CO_3_ (5 equiv.), toluene : H_2_O (10 : 1, v : v), 110 °C, 18 h.

Due to the promise of these calculations, we proceeded to prepare a family of LCn derivatives with chain lengths of *n* = 1, 4, 8, 12, 16 *via* a Suzuki–Miyaura cross-coupling of the pinacol borolanes 3–7 with a brominated DOBNA derivative 8 in 38–51% yield (Scheme 1). The identity and purity of the 5 derivatives were determined by a combination of ^1^H and ^13^C-NMR spectroscopy and HRMS. The preparation of the aromatic core 8,^32^ the pinacol borolanes 3–7,^56–59^ and the emitter BCzBN^38^ followed modified literature procedures that are outlined in Schemes S1–S3 and Table S1.‡

### Mesomorphic properties

**Fig. 2:**
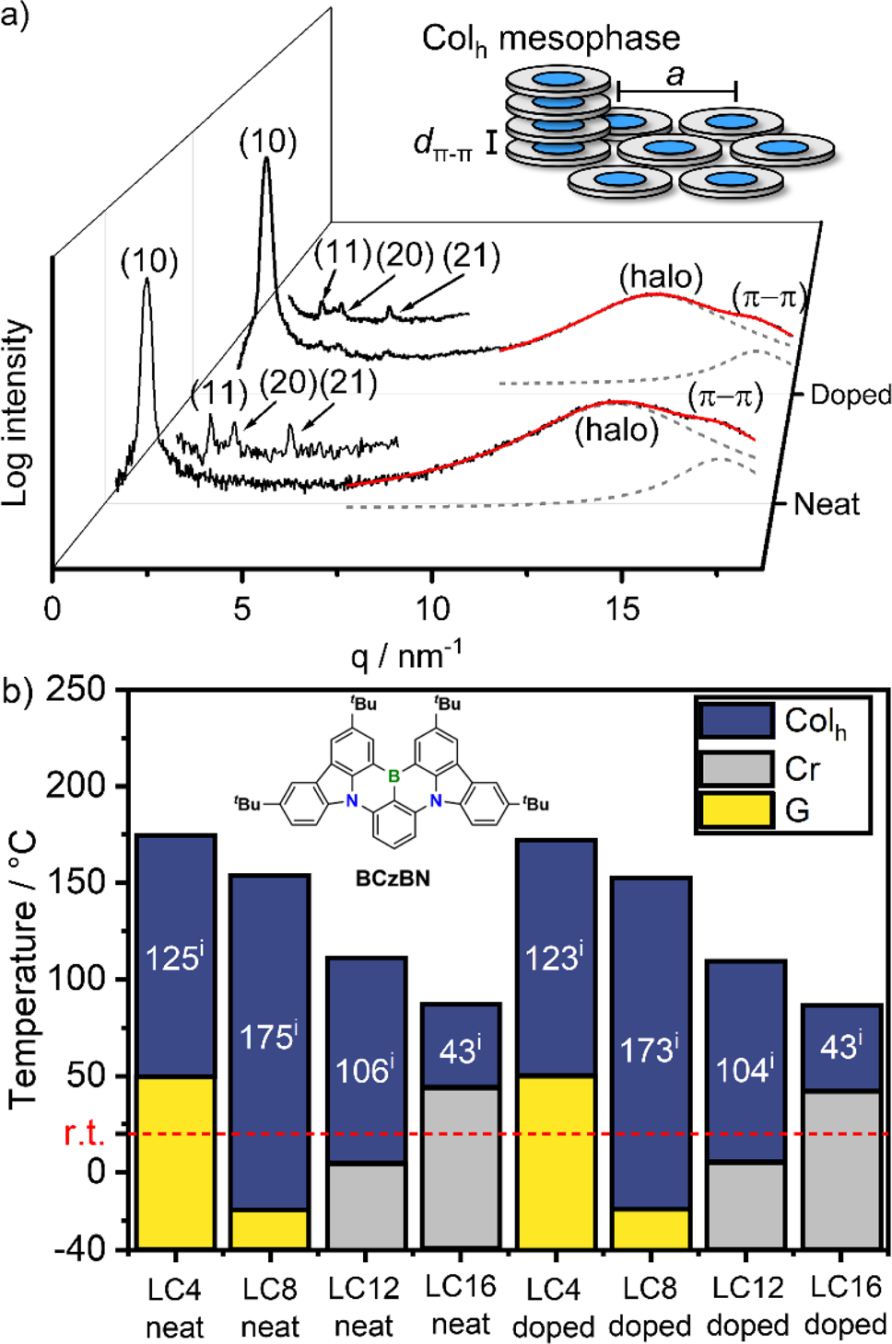
(a) WAXS diffractograms of LC8 in neat and doped form (1 wt% BCzBN) at 29 °C, also showing magnified SAXS regions. The wide-angle regimes have been fitted (red trace) with two Lorentzian functions (dashed grey traces) for halo- and π–π reflections, respectively, inset shows the schematic assembly of a Col_h_ mesophase with the π–π distance *d*_π–π_ and the lattice parameter *a*; (b) phase behaviour of the mesomorphic LCn derivatives in neat and doped (1 wt% BCzBN) form (i) phase widths of the Col_h_ mesophases. Conditions: DSC during cooling (10 K min^−1^), POM during cooling (1 K min^−1^), Cr = crystalline, G = glass. For details, please see ESI Tables S3 and S5‡ as well as Fig. S29 and S36.‡

With the materials LC1–LC16 in hand, we investigated their mesomorphic properties in neat and doped (1 wt% BCzBN) powders using polarized optical microscopy (POM), differential scanning calorimetry (DSC) and small angle (SAXS) as well as wide angle X-ray scattering (WAXS) measurements. To our delight, all derivatives except LC1 (melting point: 291 °C), exhibit mesomorphic behaviour in neat form exemplarily discussed for LC8. DSC investigation of LC8 revealed only a clearing point (*T*_c_) at *T*_c_ = 154.6/154.1 °C during heating/cooling, respectively (rate: 10 K min^−1^), and there were no other apparent phase transitions (Fig. S29c‡). POM measurements revealed that upon cooling from the isotropic phase, LC8 exhibited fan-shaped textures with line defects (Fig. S28b‡) that could be sheared and vitrified below −20 °C, indicating that it exists as a columnar mesophase. Further, SAXS investigation revealed the (10), (11), (20) and (21) reflections in characteristic 1 : 1/√3 : 1/2 : 1/√7 ratios of a hexagonal lattice (Fig. 2 and ESI, Table S4‡).^60^ The wide-angle regime consisted of the superposition of a broad halo (*d*_halo_ = 4.43 Å) and a π–π reflection (*d*_π–π_ = 3.59 Å), resulting from the molten alkyl chains and the tightly stacked aromatic cores of LC8, respectively. Accordingly, LC8 exists as an enantiotropic Col_h_ mesophase between −20 and 155 °C, with tightly stacked aromatic cores (*d*_π–π_ = 3.59 Å) and a lattice parameter (*i.e.*, distance of neighbouring molecules) of *a* = 31.4 Å (Fig. 2a).

The mesophases of the other derivatives LC4, LC12 and LC16 were analogously assigned as Col_h_ mesophases (Table S4 and Fig. S31–S34‡), albeit having different transition temperatures (Fig. 2b). Please note, LC4 did not show a (20) reflection (Fig. S31b‡) and LC12 only displayed the (10) reflection in the SAXS regime (Fig. S33b‡). The mesophases of both DLCs were nonetheless assigned as Col_h_: for LC4 this is due to its characteristic hexagon in the (10) reflection^57^ (Fig. S31a‡), while in the case of LC12 this is due to the characteristic POM textures (Fig. S28c‡). Within the series, the clearing temperatures decreased with increasing chain length from *T*_c_ = 175 °C (LC4) to *T*_c_ = 86 °C (LC16) (Fig. 2b). Both short chain derivatives LC4 (*T*_g_ = 50 °C) and LC8 (*T*_g_ = −20 °C) vitrified as glasses while the longer chain derivatives LC12 (*T*_m_ = 5 °C) and LC16 (*T*_m_ = 44 °C) crystallized upon cooling. Consequently, LC8 exhibited the widest Col_h_ mesophase with a phase range of 175 °C. Doping of LC4–LC16 with BCzBN (1 wt%) did not alter the Col_h_ assembly as evident from similar POM textures, DSC traces, and WAXS/SAXS diffractograms (Fig. S35–S40, Tables S5 and S6‡), exemplarily demonstrated from the WAXS pattern of LC8 (Fig. 2a). As shown in Fig. 2b, doping of each of the LCn series with BCzBN has no effect on the phase transition temperatures. These findings indicate no significant interaction between BCzBN and LCn hosts in the mesophase.^61,62^

### Optoelectronic properties of LCn

Cyclic voltammetry (CV) and differential pulse voltammetry (DPV) measurements were used to deduce the HOMO/LUMO levels of LC1–LC16 (Fig. S27‡). From the DPV, oxidation, *E*_ox_ = 1.25–1.36 V, and reduction potentials *versus* SCE in DCM, *E*_red_ = −1.81 to −1.73 V, for the LCn series were extracted, corresponding to orbital energies of *E*_HOMO_ = −6.16 to −6.05 eV and *E*_LUMO_ = −3.04 to −3.07 eV (Table S2‡). These are slightly stabilized compared to the calculated HOMO/LUMO levels (*E*_HOMO_ = −5.82 to −5.64 eV and *E*_LUMO_ = −1.73 to −1.60 eV). Compared to those of DOBNA (*E*_HOMO_ = −5.90 eV, *E*_LUMO_ = −2.80 eV, Table S2 and Fig. S26‡), HOMO and LUMO are slightly stabilized as observed in phenyl-substituted DOBNA derivatives.^10^

The photophysical properties of the family of LCn derivatives were unsurprisingly found to be very similar. Thus, we discuss the photophysics of LC8 as a model compound followed by identification of trends in the series.

The absorption spectrum of a toluene solution of LC8 exhibits two strong bands at *λ*_abs_ = 340 nm (*ε* = 3.9 × 10^4^ M^−1^ L^−1^) and *λ*_abs_ = 393 nm (*ε* = 3.0 × 10^4^ M^−1^ L^−1^). The low energy band at *λ*_abs_ = 393 nm is associated with the SRCT transition within the substituted DOBNA core (Fig. S2‡) and is red-shifted compared to DOBNA (*λ*_abs_ = 376 nm).^10^ The band at *λ*_abs_ = 340 nm is absent in DOBNA but is found in phenyl-substituted DOBNA derivatives and is thus assigned to a locally excited state of the mesogenic groups of LC8.^10^

**Fig. 3:**
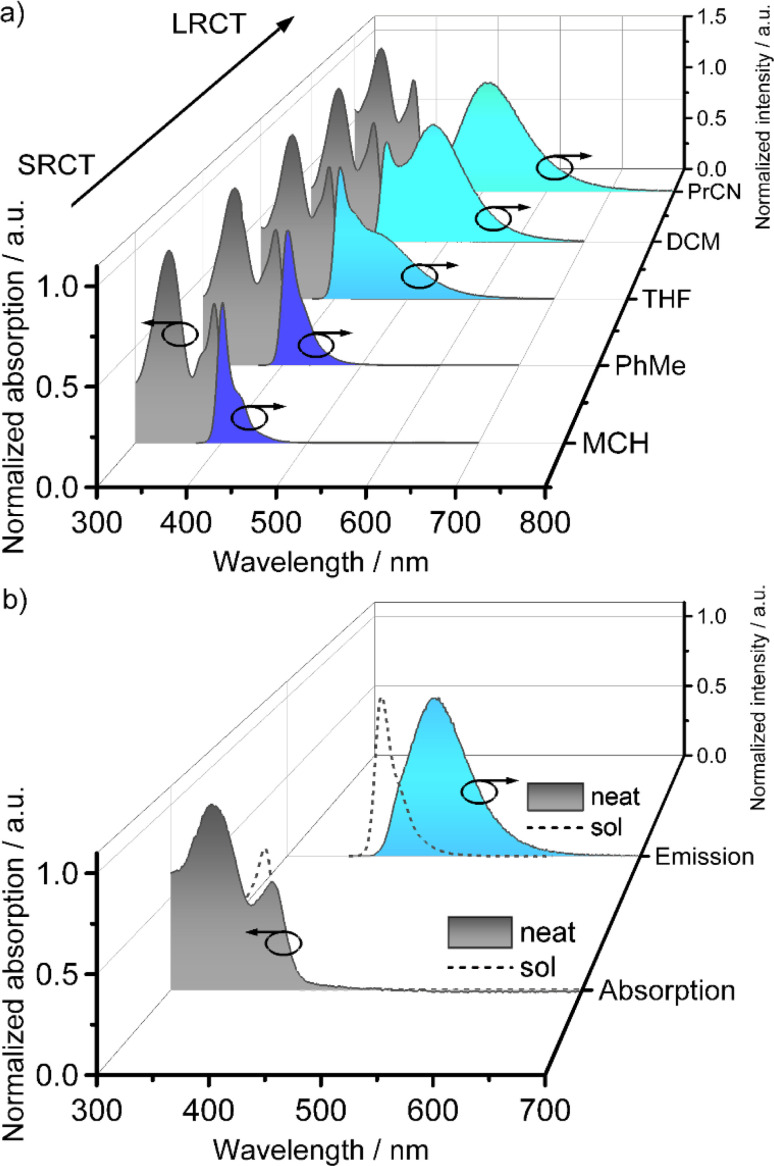
(a) Normalized absorption (black traces) and PL (blue traces) spectra of LC8 in solvents of different polarity (*c* = 0.02 mM); (b) absorption (black trace) and PL (blue trace) spectra of a spin-coated film of neat LC8 in comparison with solution-state spectra in toluene (dashed traces). *λ*_exc_ = 350 nm, MCH = methylcyclohexane, PhMe = toluene, THF = tetrahydrofurane, DCM = dichloromethane, PrCN = butyronitrile, SRCT = short-range charge transfer, LRCT = long-range charge transfer.

The photoluminescence (PL) spectrum shows deep blue emission at *λ*_PL_ = 408 nm (Fig. 3a). As expected, there is a small Stokes shift Δ*ν̄* = 1000 cm^−1^ (15 nm), a narrow full width at half maximum (FWHM) of 24 nm for the PL and a high photoluminescence quantum yield (*Φ*_PL_) of 63%, all characteristic of the short-range charge transfer (SRCT) emissive excited state of the DOBNA core in LC8. The PL band of LC8 is ∼10 nm bathochromically shifted compared to DOBNA (*λ*_PL_ = 398 nm, *Φ*_PL_ = 72%).^10^

The absorption spectra of LC8 do not show any shift when varying the solvents polarity (Fig. 3a). However, the PL band becomes broader and there is the emergence of a new shoulder peak in the range of 478–503 m with increasing solvent polarity. This reflects the presence of two emissive states, one the SRCT state and the other a long-range charge transfer (LRCT) state that becomes stabilized in more polar solvents, behaviour that has been similarly reported for donor-substituted MR-TADF molecules.^63–65^ As with most MR-TADF compounds, LC8 exhibits only prompt emission in toluene (Fig. S44b‡), with a lifetime (*τ*_P_) of 3.65 ns. From the onsets of the prompt fluorescence and delayed emission spectra in frozen toluene, LC8 had a S_1_ energy of 3.16 eV and a T_1_ energy of 2.70 eV, much more stabilized than the calculated values of S_1_ = 3.76 eV and T_1_ = 3.44 eV. The associated Δ*E*_ST_ of 460 meV (Fig. S44e‡) is likely too large for TADF and also larger than the calculated Δ*E*_ST_ = 0.31 eV. The large Δ*E*_ST_ of LC8 compared to DOBNA (S_1_ = 3.12 eV, T_1_ = 2.97 eV, Δ*E*_ST_ = 150 meV in frozen EtOH)^10^ can be rationalized by the mixed SRCT/LRCT character of the emissive excited state of LC8 induced through the presence of the electron-rich mesogenic groups.

As expected, the presence of different chain lengths did not influence the solution-state photophysics within the LCn series (Table S7‡). All members of the LCn series exhibited SRCT emission (*λ*_PL_ = 407–408 nm) in low polar solvents as well as LRCT emission of increasing intensity in solvents of increasing solvent polarity (Fig. S42–S46, Tables S7 and S8‡). The *Φ*_PL_ values in toluene ranged between 58–63%, while Δ*E*_ST_ ranged between 0.44 and 0.46 eV.

Next, we investigated the neat photophysics of the LCn series in spin-coated films compared to the dilute toluene solution, the absorption bands of a neat film of LC8 are slightly broadened and the absorption maxima bathochromically shifted to *λ*_abs_ = 341 and 399 nm (Fig. 3b). This indicates only a limited number of ground-state intermolecular interactions of the LC8 molecules despite their tight stacking in the Col_h_ mesophase as reported for the related LC 2 and BON-LC.^32,33^ The *λ*_PL_ of neat DOBNA-LC8 is bathochromically shifted to 472 nm and the PL spectrum is broadened (FWHM = 70 nm), attributed to excimer emission as also observed for the DOBNA based LC 2 and BON-LC (Fig. 3b).^32,33^ The time-resolved PL decay of the neat film of LC8 is complex, modelled as triple exponential decay (*τ*_p,avg_ = 16.62 ns) and no delayed emission was observed. The S_1_ and T_1_ energies of LC8 are 2.92 and 2.55 eV, respectively, resulting in a somewhat smaller Δ*E*_ST_ of 370 meV compared to the Δ*E*_ST_ of 460 meV in toluene. Expectedly for excimers, both the S_1_ and T_1_ energies of the neat film of LC8 (Fig. S49‡) are lower than those in dilute toluene solution. The *Φ*_PL_ of neat LC8 decreased to 19% due to strong aggregation-caused quenching (ACQ).

The photophysics of neat films of the other LCn derivatives do not differ much compared to those of LC8 (ESI, Table S9 and Fig. S47–S51‡). Overall, the photophysics of the neat films of the family of LCn compounds compared to those in toluene are dominated by a bathochromically shifted (*λ*_PL_ = 434–472 nm) and broadened (FWHM = 52–80 nm) excimer emission as discussed in detail for LC8. The *Φ*_PL_ values are lower at *Φ*_PL_ = 6–22%. None of these derivatives exhibited TADF due to their high Δ*E*_ST_ = 340–440 meV. The S_1_ energies range between 2.91–2.98 eV and the T_1_ energies range between 2.50–2.57 eV. These small differences within the neat films can be explained by differences in the individual packing at r.t. and thus the strength of the intermolecular interactions in the excimers.

### Optoelectronic properties of LCn doped with BCzBN

Inspired by DOBNA-based host materials such as DOBNA-Tol, we investigated the LCn series as a host materials due to their large S_1_ = 2.91–2.98 eV and T_1_ = 2.50–2.57 eV levels. We doped the LCn series with the MR-TADF emitter BCzBN (S_1_ = 2.49 eV, T_1_ = 2.38 eV in a frozen EtOH matrix^38^). BCzBN was chosen as the emitter as there is a large spectral overlap between its absorption with the emission of neat films of the LCn series (Fig. S57‡), which is a prerequisite for an efficient Förster Resonance Energy Transfer (FRET) from host (LCn) to guest (BCzBN). As previously reported, 1 wt% doped films of BCzBN in 3,3′-di(9*H*-carbazol-9-yl)-1,1′-biphenyl (*m*CBP) showed bright, sky-blue emission with *λ*_PL_ = 493 nm, FWHM = 35 nm and *Φ*_PL_ = 88%,^50^ but as a neat film suffers from excimer formation and severe ACQ, reflected in the much lower *Φ*_PL_ of 32%.^66^ Since all materials of the LCn series were found to behave similarly, we will discuss the photophysics of LC8 doped with BCzBN (BCzBN:LC8) in detail, followed by a discussion of trends within the doping series BCzBN:LCn. Excitation of the doped films was set to *λ*_exc_ = 350 nm, which is a wavelength with almost exclusive absorption through the respective LCn host to avoid direct excitation of BCzBN (see Fig. S52‡ for qualitative comparison of LCn and BCzBN absorption).

**Fig. 4:**
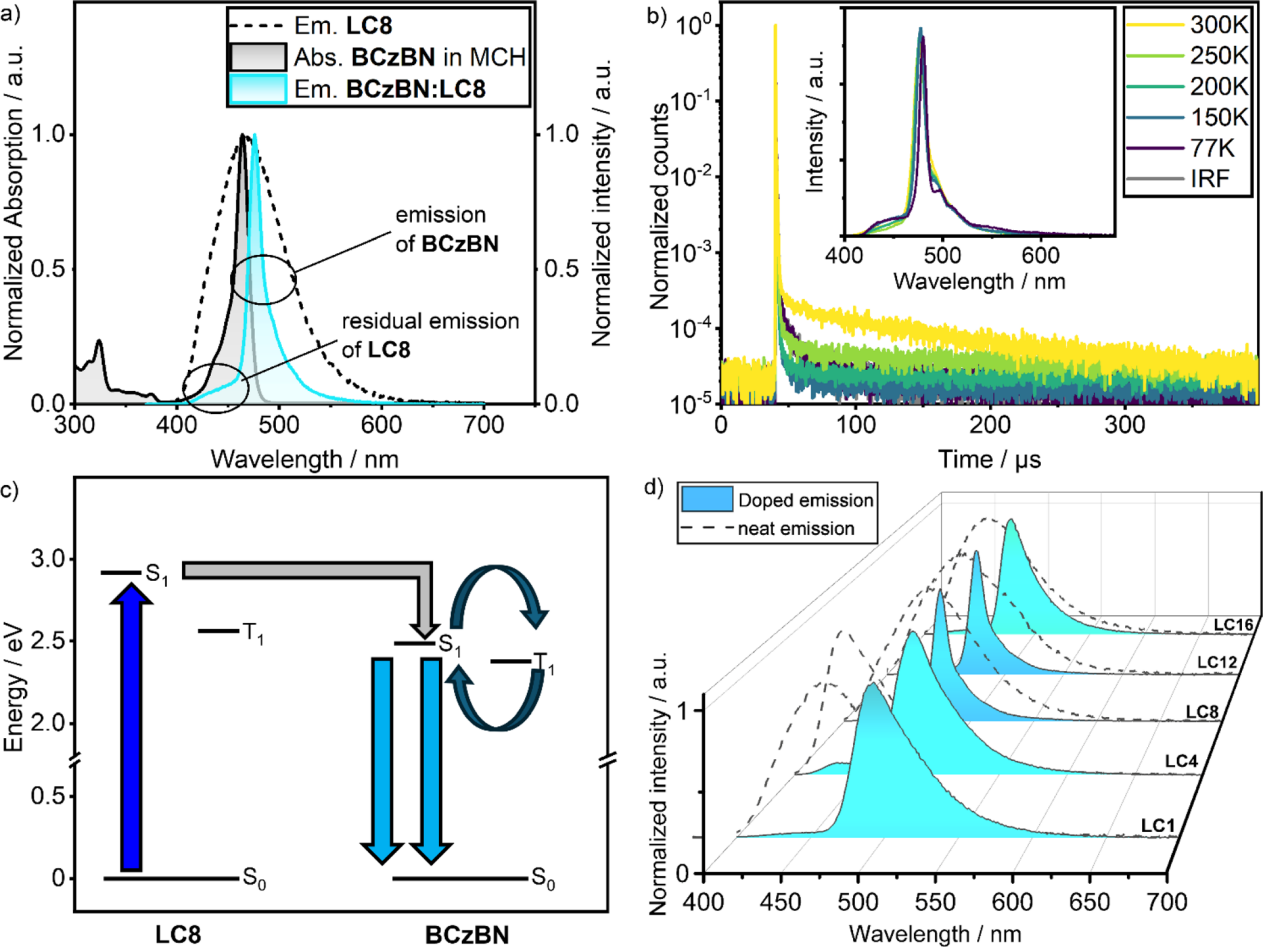
(a) Steady state emission of BCzBN doped in the LC8 series (1 wt%), neat emission of LC8 (black dashed trace) and absorption of BCzBN in methylcyclohexane (MCH, *c* = 0.02 mM) given for comparison (black trace); (b) temperature dependent lifetime measurements of LC8 doped with BCzBN, inset shows the corresponding steady state emission; (c) emission pathway of LC8 doped with BCzBN; (d) steady state emission of BCzBN doped in the LCn series (*c* = 1 wt%, blue traces), neat emission of the respective LCn derivative given for comparison (black dashed traces). Conditions for films: spin-coated from CHCl_3_, *c* = 1 wt%, steady state: *λ*_exc_ = 350 nm, time resolved: *λ*_exc_ = 379 nm.

The PL of BCzBN:LC8 is narrow (FWHM = 14 nm) and peaks at *λ*_PL_ = 476 nm, with *Φ*_PL_ = 41% (Fig. 4a). This band displays the PL characteristics of the BCzBN dopant (*λ*_PL_ = 472 nm, FWHM = 16 nm, 0.02 mM in methylcyclohexane, Fig. S55‡) in a low polarity environment. Compared to the PL characteristics of 1 wt% doped films of BCzBN in *m*CBP (*λ*_PL_ = 493 nm, FWHM = 35 nm, *Φ*_PL_ = 88%), the emission of 1 wt% BCzBN:LC8 is blue-shifted, significantly narrowed, and features a lower *Φ*_PL_.^50^ The excitation energy is thus efficiently transferred from the LC8 host to the BCzBN guest *via* FRET (Fig. 4c); we note that this energy transfer is not quantitative as residual emission from the LC8 host is apparent as a tiny shoulder at *λ*_PL_ ∼450 nm (Fig. 4a). From the integrals of the PL of the mixture BCzBN:LC8 and the integral of the scaled PL of the host LC8, an observed FRET efficiency, also known as a proximity ratio *E*_PR_ = 74% has been calculated (Fig. S60 and Table S13‡). We wish to note that the determination of the absolute FRET efficiencies is complicated and *E*_PR_ thus serves only as a rough estimation for the energy transfer efficiency in BCzBN:LC8 as it does not account for spectral narrowing (or broadening as is the case in BCzBN blends with LC1 and LC4) compared to that of BCzBN:LC8.^67^

TCSPC experiments on BCzBN:LC8 revealed a complex decay with an average prompt lifetime of *τ*_p_ = 8.19 ns (triple exponential fit, Fig. S55b‡), comparable to the *τ*_p_ = 8.50 ns reported for BCzBN in *m*CBP (1 wt%).^50^ In addition to the prompt luminescence of BCzBN:LC8, investigation *via* multi-channel scaling (MCS) allowed us to detect delayed emission with a delayed lifetime (*τ*_d_) of 108.5 μs under oxygen-free environment. The delayed lifetime component vanishes in the presence of air, hinting at involvement of oxygen-sensitive T_1_ states (ESI, Fig. S55c‡). Compared to the 1 wt% doped films of BCzBN in *m*CBP (*τ*_d_ = 68.8 μs), the *τ*_d_ of BCzBN:LC8 is longer.^50^ Temperature-dependent (77–300 K) time-resolved PL decay measurements confirmed the endothermic nature of the delayed emission (Fig. 4b), which is consistent with TADF.^1^

All films of the BCzBN:LCn series exhibit narrowband (FWHM = 14–47 nm) emission, originating mainly from the BCzBN guest (*λ*_PL_ = 476–493 nm, Fig. 4d and Table 1). The FRET efficiency *E*_PR_ = 84–94% was higher for the other mixtures of the BCzBN:LCn series (Fig. S60 and Table S13‡). This could result from a decreased distance between host and emitter in the short chain mixtures BCzBN:LC1 and BCzBN:LC4 as well as an increased molar concentration of BCzBN in the mixtures BCzBN:LC12 and BCzBN:LC16. The other r.t. LC host–guest system, BCzBN:LC12, have similar narrowband emission (*λ*_PL_ = 475 nm, FWHM = 15 nm) compared to BzCBN:LC8. While BCzBN:LC16 has a similar *λ*_PL_ of 479 nm, its spectrum is broader (FWHM = 31 nm). Both short-chain derivatives BCzBN:LC1 (*λ*_PL_ = 493 nm, FWHM = 47 nm) and BCzBN:LC4 (*λ*_PL_ = 487 nm, FWHM = 42 nm) show red-shifted and broadened emission compared to BCzBN:LC8 that are also comparable to the values reported for BCzBN in *m*CBP (*λ*_PL_ = 493 nm, FWHM = 35 nm). Notably, all films of the BCzBN:LCn series except for BCzBN:LC1 show delayed emission, with similar oxygen and temperature dependency as found for BCzBN:LC8 (Fig. S53–S57‡). The *τ*_d_ = 91.5–108.5 μs of the longer chain host–guest blends BCzBN:LC8, BCzBN:LC12 and BCzBN:LC16 are similar, while BCzBN:LC4 has a much shorter delayed lifetime of *τ*_d_ = 37.8 μs (Table 1). The *Φ*_PL_ = 68% of BCzBN:LC4 is higher than for the other mixtures (*Φ*_PL_ = 33–50%, Table 1). The moderate *Φ*_PL_ = 41–44% of the rt LC mixtures BCzBN:LC8 and BCzBN:LC12 might be explained by the flexible liquid crystalline matrix contributing to non-radiative decay and the non-quantitative FRET (*E*_PR_ = 74 or 84%). These issues could be addressed by designing a stiffer host LC and by increasing the BCzBN concentration to facilitate the energy transfer. Compared to the rt TADF-LCs 1a (*Φ*_PL_ = 4%) and 1b (*Φ*_PL_ = 20%), our host guest approach shows a markedly improved *Φ*_PL_ = 41–44%.^26^ More importantly, BCzBN:LC8 and BCzBN:LC12 conserve the delicate MR-TADF character of BCzBN despite the strong aggregation in the Col_h_ mesophase usually resulting in excimer emission as is the case in 2 and BON-LC. Our host–guest approach can thus act as a design principle for liquid crystalline TADF materials having narrowband emission and is not limited to the host LC/emitter combination used in this work.

**Table 1:** Key photophysical properties of the LCn series in neat and doped (1 wt% BCzBN) films

#	Conditions	*λ*_PL_ (FWHM)/nm	*Φ*_PL_/%	*τ*_P_ a/ns	*τ*_d_/μs
LC1	Neat	459 (74)	6	6.98	—
BCzBN:LC1	Doped	493 (47)	33^b^	5.51	—
LC4	Neat	434 (52)	22	6.66	—
BCzBN:LC4	Doped	487 (42)	68^b^	8.03	37.8
LC8	Neat	472 (70)	19	16.62	—
BCzBN:LC8	Doped	476 (14)	41^b^	8.19	108.5
LC12	Neat	463 (88)	19	11.87	—
BCzBN:LC12	Doped	475 (15)	44^b^	4.04	91.5
LC16	Neat	456 (80)	20	9.95	—
BCzBN:LC16	Doped	479 (31)	50^b^	6.46	103.1

a Given as amplitude average lifetime.

b Reflecting *Φ*_PL_ of BCzBN in the respective LCn matrix.

### Packing model of LCn doped with BCzBN

**Fig. 5:**
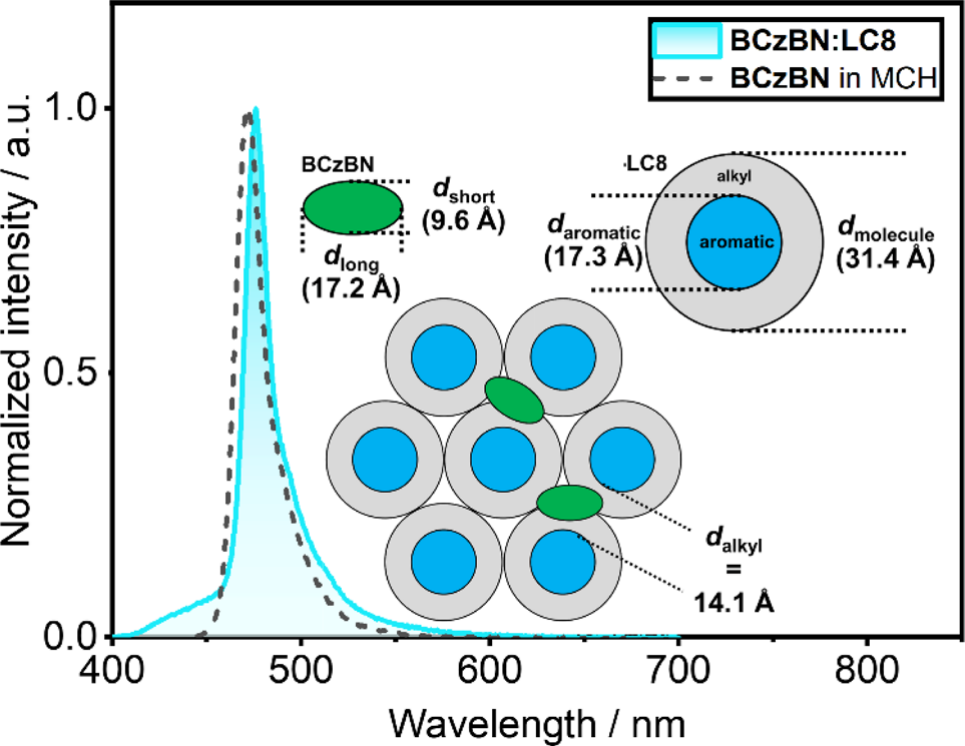
Normalized emission of BCzBN in methylcyclohexane (MCH, 0.02 mM, black dashed trace) and in the BCzBN:LC8 host–guest system (1 wt%, blue trace). Inset shows a proposed packing model for BCzBN:LC8 with BCzBN assembling in the alkyl chains of LC8. The model is derived from SAXS data (*d*_molecule_ = 31.4 Å), DFT calculations (*d*_aromatic_ = 17.3) and the single crystal X-ray structure (CCDC: 2032785)^49^ of BCzBN (*d*_long_ = 17.2 Å, *d*_short_ = 9.6 Å).

From our investigations of the mesomorphic properties of the BCzBN:LCn series, that reveal no influence from the BCzBN guest on the mesophase structure, we deduce BCzBN to be dispersed in the (liquid) crystalline phases of the respective DOBNA-LC host. This finding is supported by the photophysics of the BCzBN:LCn series, showing emission from isolated BCzBN molecules and no excimer emission from BCzBN aggregates or exciplex emission from DOBNA-BCzBN assemblies. However, starting from the shortest chain system BCzBN:LC1 (*λ*_PL_ = 493 nm, FWHM = 47 nm), the emission bands blue-shift and narrow until reaching a minimum for the r.t. LCs BCzBN:LC8 (*λ*_PL_ = 476 nm, FWHM = 14 nm) and BCzBN:LC12 (*λ*_PL_ = 475 nm, FWHM = 15 nm). A possible explanation for the blue-shift and narrowing of the emission bands could be that the greater density of the aliphatic chains (Fig. S41‡) leads to a less polar medium experienced by BCzBN.^36,68,69^ This is also consistent with the fact that the PL of BCzBN:LC8 (Fig. 5) and BCzBN:LC12 (Fig. S58b‡) strikingly resemble the emission of BCzBN in dilute methylcyclohexane, which implies BCzBN being dispersed in a low polar environment in these r.t. LC systems.^68^ Columnar liquid crystals such as LC8 offer such a low polarity environment in form of the alkyl chains (Fig. 5, grey), separating the stacked aromatic cores (Fig. 5, blue, *d*_aromatic_ = 17.3 Å, DFT calculations) in the mesophase.^19,21^ The narrowest distance within the alkyl domain, *d*_alkyl_ = *d*_molecule_ − *d*_aromatic_ = 14.1 Å, can be calculated from the discoid diameter *d*_molecule_ = 31.4 Å, extracted from the SAXS data (*d*_molecule_ = *a*). Assuming BCzBN as an ellipsoid (green, Fig. 5) with dimensions along the long (*d*_long_ = 17.2 Å) and short molecular axes (*d*_short_ = 9.6 Å) extracted from single crystal X-ray diffraction data (CCDC: 2032785),^49^BCzBN would geometrically fit into the alkyl domain of LC8 (Fig. 5).

We thus propose a packing model in which the BCzBN guest is dispersed in the alkyl domain of the Col_h_ mesophase of BCzBN:LC8.

A similar packing model is proposed for the BCzBN:LC12 system (ESI, Fig. S59b‡). The non r.t. LC systems BCzBN:LC1, BCzBN:LC4 and BCzBN:LC16 exhibit crystalline phases of unknown structure at r.t. and their photophysical behaviour thus cannot be explained by the packing model proposed for BCzBN:LC8 and BCzBN:LC12. The narrowband emission of the BCzBN:LC1, BCzBN:LC4 and BCzBN:LC16 mixtures identified as emission from the guest BCzBN, however, suggests a random dispersion of the BCzBN guest in the respective matrices.

## Conclusions

In summary, we prepared a series of liquid crystalline host materials, LCn, based on the MR-TADF DOBNA core functionalized with mesogenic groups bearing alkoxy chains of different chain length. Depending on the chain length, these derivatives exhibited Col_h_ mesophases with phase widths of greater than 100 °C and room temperature mesomorphism. Their neat photophysics is dominated by broad excimer emission due to the strong aggregation required for Col_h_ formation. When the guest MR-TADF emitter BCzBN is added at 1 wt% doping, efficient FRET is observed, allowing for the first time a r.t. LC system that shows narrowband TADF and high *Φ*_PL_. Since many of the favourable properties associated with LCs (*e.g.*, charge mobility and boosted chiroptical properties) depend on the presence of a LC phase, this work is an important step to demonstrating the utility of MR-TADF LCs in organic optoelectronics. In a general context, this work outlines a novel host–guest strategy for TADF-LCs that combines both the strong intramolecular interactions required for LC formation with the favourable TADF properties of a dilute emitter. With many host and emitter moieties available for the construction of liquid crystalline host–guest systems, we believe this work will inspire scientists to design new self-assembling materials for solution-processable optoelectronic applications.

## Author contributions

J. A. K. conceived and supervised the project, performed part of the synthesis as well as characterization and wrote the first draft of the manuscript. F. M. performed part of the synthesis and characterization. T. M. and A. K. G. helped with characterization computation and manuscript preparation and J. M. D. S. provided BCzBN as well as helped with manuscript preparation. E. Z.-C. and S. L. supervised the project and the manuscript preparation.

## Conflicts of interest

There are no conflicts to declare.

## Supplementary Material

SC-015-D4SC04429K-s001

## Data Availability

The research data supporting this publication can be accessed at https://doi.org/10.17630/7ee54fc1-2819-4d08-a726-439ceab8434c.
