# Supplementary material for: MR-TADF liquid crystals: towards self assembling host–guest mixtures showing narrowband emission from the mesophase
Source: Chem Sci. 2024 Oct 11;15(43):18022–30. doi: 10.1039/d4sc04429k (PMC11467995; doi:10.1039/d4sc04429k)
Supplement: SC-015-D4SC04429K-s001 [file SC-015-D4SC04429K-s001.pdf]

Supplementary Information (SI) for Chemical Science.
This journal is © The Royal Society of Chemistry 2024

# Electronic Supporting Information (ESI)

# MR-TADF Liquid Crystals: Towards Self Assembling Host-Guest Mixtures Showing Narrowband Emission from the Mesophase

Julius A. Knöller,*a Franziska Müller,a Tomas Matulaitis,b John M. Dos Santos,b Abhishek Kumar Gupta,b Eli Zysman-Colman*b and Sabine Laschat*a

[a] Institut für Organische Chemie, Universität Stuttgart, Pfaffenwaldring 55, D-70569, Stuttgart, Germany. Email: sabine.laschat@oc.uni-stuttgart.de.
[b] Organic Semiconductor Centre, EaStCHEM School of Chemistry, University of St Andrews, St Andrews, Fife, UK, KY16 9ST, Fax: +44-1334 463808; Tel: +44-1334 463826; E-mail: eli.zysman-colman@st-andrews.ac.uk.

## Table of Contents

1. General Methods ........ S2
 1.1. Quantum chemical calculations: ........ S2
 1.2. Synthesis ........ S2
 1.3. Photophysical properties ........ S3
 1.4. Mesomorphic properties ........ S5
2. Theoretical Calculations ........ S6
3. Synthesis ........ S7
 3.2.1. Compounds prepared via literature procedures ........ S7
 3.2.2. Synthesis of the aromatic core **8** ........ S8
 3.2.3. Synthesis of the pinacolborolanes **3** - **7** ........ S10
 3.2.4. Synthesis of **LC1** – **LC16**: ........ S14
 3.2.5. Synthesis of the emitter **BCzBN** ........ S20
4. NMR/ HRMS spectra ........ S22
5. Electrochemical Properties of the **LC** series ........ S45
6. Mesomorphic Properties ........ S47
 6.2. Mesomorphic Properties of the **LC** Series ........ S47
 6.3. Mesomorphic Properties of the **LC** series doped with **BCzBN** (1wt%) ........ S55
7. Photophysical Properties ........ S61
 7.2. Photophysical Properties of the **LC** Series in Solution ........ S61
 7.3. Photophysical Properties of the **LC** Series in Neat Films ........ S67
 7.4. Photophysical Properties of the **LC** Films doped with **BCzBN** (1wt%) ........ S73
8. Literature ........ S81

# 1. General Methods

## 1.1. Quantum chemical calculations:

The calculations were performed with the Gaussian 16 revision C.01 suite[1]for the density functional theory (DFT) calculations with the ORCA software package 5.0.3[2,3] for double-hybrid DFT (DH-DFT). DFT was performed with the PBE0 functional[4], 6-31G(d,p)[5] and GD3BJ empirical dispersion in the gas phase. Double hybrid DFT was performed using the wPBEPP86 functional[6] and cc-pVDZ basis set[7] in the gas phase. Molecular orbital energies and isocountour plots were obtained based on the gas-phase ground state-optimized structures using the PBE0 functional. Excited-state calculations were performed using the double hybrid (DH) wPBEPP86 functional and were we first optimized on the ground state at the wPBEPP86 level and vertical excitations to excited states were calculated based on this ground state-optimized structure. All calculations were submitted and processed using the Silico v5 software package[8,9], which incorporates a number of publicly available software libraries, including: cclib[10] for the parsing of result files, VMD[11]/Tachyon[12] for 3D rendering, Matplotlib[13] for plotting of graphs, Open Babel[14]/Pybel[15] for file interconversion and PySOC[16] for the calculation of spin-orbit coupling.

## 1.2. Synthesis

**Methods and Chemicals:**

If not indicated otherwise, all chemicals were commercially available and have been used without further purification. Moisture and/ or air sensitive reagents were manipulated by Schlenk technique in flame dried glassware and in a MBraun glovebox. Dry Toluene, $CH_2Cl_2$ and THF were distilled over sodium, $CaH_2$ and potassium, respectively. NMP, DMF, Xylene, $Et_2O$ were dried over 4Å molecular sieves and degassed by bubbling with $N_2$.[17] Petroleum ether, EtOAc and $CH_2Cl_2$ were distilled prior to use.

**Thin layer chromatography (TLC):**

Reaction monitoring and/or column chromatography was performed on TLC plates Alugram® Xtra SIL G/$UV_{254}$ from Macherey-Nagel as stationary phase with the indicated solvent mixture as mobile phase. Substance spots were identified by illuminating the plates with UV light (254 nm and 366 nm) or by immersing the plate in one of the following staining solutions followed by heating to 300 °C:

Cerium molybdate: $Ce(NH_4)_4(NO_3)_6$ (0.5 g) and $(NH_4)_6Mo_7O_{24} \cdot 4\ H_2O$ (12 g) were dissolved in deionized $H_2O$ (200 mL) and acidified with conc. $H_2SO_4$ (28 mL).

$KMnO_4$: $KMnO_4$ (0.375 g), $K_2CO_3$ (2.5 g) and NaOH (0.1 g) were dissolved in deionized $H_2O$ (50 mL).

**Column chromatography:**

Column chromatography was performed on Silica SiliFlash® F60 from Silicycle as the stationary phase using the indicated solvent mixture as mobile phase. The columns were dry packed with silica and flushed with the indicated mobile phase. The crude products were applied on the column as a solution in the mobile phase if not indicated otherwise. Diameters (d) and lengths (l) of the silica was indicated in each procedure.

Borylated silica for purification of boronic acids and boronic esters was prepared according to a literature procedure from silica gel and boric acid.[18]

**Nuclear magnetic resonance spectroscopy:**

$^{1}$H-NMR spectra were recorded at frequencies of 400/ 500/ 700 MHz, $^{11}$B-NMR spectra were recorded at frequencies of 128/ 160/ 224 MHz, broadband decoupled $^{13}$C-NMR spectra were recorded at frequencies of 100/ 125/ 176 MHz, $^{19}$F-NMR spectra were recorded at frequencies of 282/ 376 MHz on Bruker AVANCE 300/ ASCEND 400/ AVANCE 500/ AVANCE 700 spectrometers, respectively. Chemical shifts were given in parts per million (ppm) and referenced to the peak of the deuterated solvents if not indicated otherwise.[19] Multiplicities were given in Hz and abbreviated as following: singlet (s), doublet (d), triplet (t), quartet (q), multiplet (m). COSY, HSQC, HMBC and NOESY spectra were utilized to assign the signals to the corresponding atoms.

**Mass spectrometry:**

High resolution mass spectra (HRMS) were measured on a Micromass GCT TOF-EI or a Micromass ZQ Single Quad ESI spectrometer.

**Infrared Spectroscopy:**

Infrared spectra were measured in solution on a Vektor 22 spectrometer by Bruker with a MKII Golden Gate Single Reflection Diamant ATR-System. The intensities of the absorption bands were classified as strong (s) and weak (w).

### 1.3. Photophysical properties

**Sample preparation**

Solution samples were prepared by dissolving the sample in the appropriate volume of HPLC grade or spectral grade solvent and eventually diluting with micropipettes to the appropriate concentration (0.01 – 0.02 mM). Degassed samples were prepared by 3 freeze/pump/thaw cycles. Thin film samples were spin-coated from $CHCl_3$ solution (c = 10 - 40 mg mL$^{-1}$) on quartz or sapphire substrates on a Laurell WS-650-Mz-23NPPB spin coater at 1000 RPM and dried at 80 °C for 10 min. Thick film samples were drop-cast from $CHCl_3$ solution (c = 10 - 20 mg mL$^{-1}$) on preheated (50 °C) quartz or sapphire substrates and dried at 80 °C for 10 min.

**UV-Vis and Fluorescence spectroscopy:**

Absorption spectra were recorded on a Shimadzu UV-2600 spectrophotometer in the indicated solvent. An integration sphere was used for recording the absorption spectra of spin-coated or drop cast films. Extinction coefficients were determined by linear regression of the absorbance at five different concentrations (c = 0.01 – 0.03 mM). Steady-state emission spectra were recorded on a FS5 spectrofluorometer from Edinburgh instruments in the indicated solvent (c = 0.02 mM) or in a drop caste / spin-coated film.

**Steady-State and Time-Resolved Photoluminescence Measurements**

Solution samples were investigated on FS-5 instrument by Edinburgh Instruments equipped with a xenon lamp and a picosecond laser source (Edinburgh Instruments EPL-375, $\lambda_{exc}$ = 373 nm) for steady-state and time-resolved measurements, respectively. Film samples were investigated on a FLS-980 instrument by Edinburgh Instruments equipped with a xenon lamp and a laser diode (Picoquant LDH-D-C-375, $\lambda_{exc}$ = 379 nm) on an Picoquant PDL-800-D driver for steady state and time resolved measurements, respectively. The film samples were mounted onto a DN-V cold finger cryostat driven by a Mercury ITC controller from Oxford instruments (T = 77 – 310 K) for variable temperature measurements under air and vacuum. Time correlated single photon counting (TCSPC) and Multi-channel scaling (MCS) experiments were conducted to record photoluminescence decays in the ns and µs regime, respectively. The corresponding decay curves were fitted to (multi)exponential functions according to the least squares method to determine the lifetimes of the excited states. If applicable, amplitude average lifetimes $\tau_{avg}$ were calculated for multiexponential decays.[20]

**Time-gated measurements:**

Singlet ($S_1$) and triplet ($T_1$) energies were determined from the onsets of fluorescence and phosphorescence spectra at 77 K, respectively. For samples in a frozen toluene matrix and for the neat samples of **LC1** – **LC8**, a custom time-gated ICCD setup consisting of a femtosecond Nd:YAG laser (Orpheus-N, model: PN13F1, $\lambda_{exc}$ = 343 nm), a spectrograph (Chromex imaging, 250is spectrograph), and a sensitive gated iCCD camera (Stanford Computer Optics, 4Picos) having sub-nanosecond resolution was employed. Prompt fluorescence was measured 1 ns after excitation with an exposure time of 100 ns, phosphorescence was recorded 1 ms after excitation with an exposure time of 9 ms. For neat films of **LC12** and **LC16**, $S_1$ energies were extracted from steady state spectra at 77 K (samples were excited using a Xe lamp, $\lambda_{exc}$ = 343 nm). $T_1$ energies were extracted from phosphorescence spectra recorded 10 ms after excitation with an exposure time of 90 ms (samples were excited using a microsecond flashlamp operating at 10 Hz, $\lambda_{exc}$ = 343 nm) on an FS-5 instrument by Edinburgh Instruments.

**Photoluminescence Quantum Yields ($\Phi_{PL}$):**

Solution-state $\Phi_{PL}$s were determined utilizing the optical dilute method[21] in air saturated and degassed toluene solutions using quinine sulfate in 0.5 M $H_2SO_4$ ($\Phi_r$ = 54.6%[22]) as standard. Four samples with absorptions of ca. 0.05, 0.0375, 0.025 and 0.0125 were prepared and their $\Phi_{PL}$s were determined by the equation $\Phi_{PL}=\Phi_r(A_r/A_s)(I_s/I_r)(n_s/n_r)^2$, where $A_r$ and $A_s$ are absorption of reference and sample, $I_r$ and $I_s$ are emission intensity of reference and sample and $n_r$ and $n_s$ are the refractive indices of reference and sample solvent, respectively. Film $\Phi_{PL}$s were determined on a FS-5 spectrometer from Edinburgh Instruments equipped with a SC30 calibrated integrating sphere ($\Delta\Phi_{PL}$ = 5%) as average of 3 measurements under air or $N_2$ atmosphere.

**Electrochemical Measurements:**

Cyclic Voltammetry (CV) and Differential Pulse Voltammetry (DPV) analysis were performed on a CHIE620E potentiostat from CH Instruments. All measurements were performed in 0.1 M electrolytes of tetrabutylammonium hexafluorophosphate ($[^{n}Bu_4N]PF_6$) in $CH_2Cl_2$ degassed by sparging with $CH_2Cl_2$ saturated $N_2$ gas. The measurement cell consisted of an $Ag/Ag^+$ reference electrode, a glassy carbon working electrode and a platinum wire as counter electrode. The redox potentials are reported relative to a saturated calomel electrode (SCE) utilizing the ferrocene/ferrocenium ($Fc/Fc^+$) redox couple (sublimed, 0.46 V vs SCE) as internal standard.[23] The HOMO and LUMO values were extracted from the anodic / cathodic DPV peak potentials according to $E_{HOMO/LUMO} = -(E_{ox} / E_{red} + 4.8)$ eV.[24]

## 1.4. Mesomorphic properties

**Differential Scanning Calorimetry (DSC):**

DSC measurements were performed on a DSC822$^e$ by Mettler Toledo in standardized 40 µL aluminum crucibles and evaluated with the software STARe 14.0. Phase transition temperatures are given as onsets of the corresponding peaks.

**Polarizing Optical Microscopy (POM):**

A polarizing optical microscope Olympus BX50 equipped with a variable temperature sample holder LTS350 (control unit: TP39 and LNP, $\Delta T = \pm 1$ K) by Linkam Scientific was used to investigate the mesomorphic properties of our compounds. Micrographs were taken with a Zeiss Axiocam 105 color camera module and the software ZEN core. The samples were investigated on regular glass slides and in polyimide coated cells.

**Wide (WAXS) and small angle (SAXS) Xray diffraction:**

For Xray diffraction studies, the samples were sealed in glass capillaries supplied by Hilgenberg GmbH (external diameter of 0.7 mm, wall thickness 0.01 mm). Two dimensional Xray diffraction (2D-WAXS and 2D-SAXS) studies were carried out on a Bruker AXS Nanostar C equipped with a ceramic tube generator ($CuK_\alpha$ radiation, $\lambda = 1.5405$ Å, 1500 W) and a Bruker Vantec 500 detector. The diffractograms were processed using SAXS software and calibrated to the diffraction pattern of silver behenate at 25 °C.

## 2. Theoretical Calculations

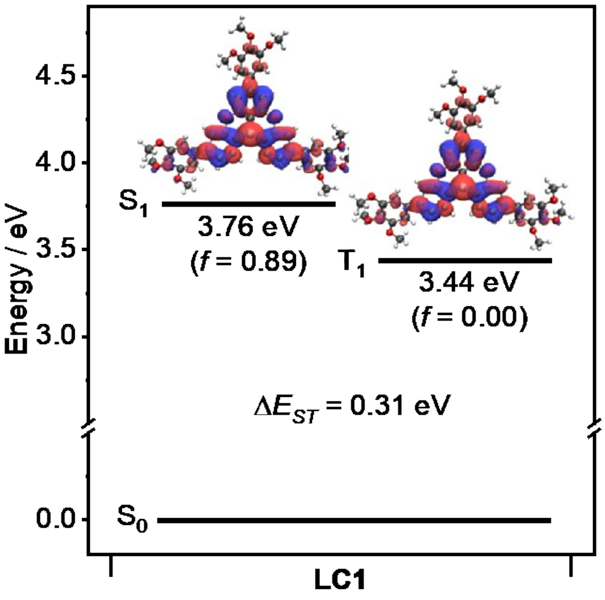


Figure **S1:** Energies of the lowest singlet and triplet excited states for **LC1** calculated via double hybrid DFT at wPBEPP86/cc-pVDZ level in the gas phase. Difference density plots for the lowest excited singlet and triplet states of **LC1** calculated at TDA-DFT-PBE0/6-31G(d,p) level in the gas phase (isovalue = 0.02, blue = holes, red = electrons).

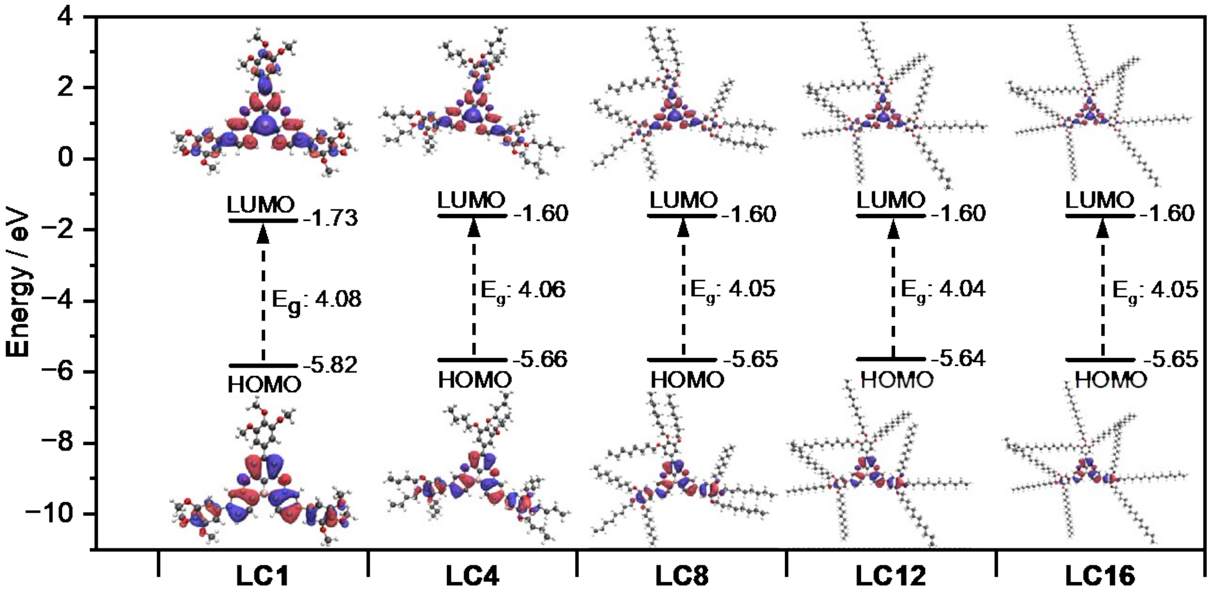


**Figure S2:** Frontier orbitals energy diagram and HOMO, LUMO plots for the **LC** series calculated at TDA-DFT-PBE0/6-31G(d,p) level in the gas phase (isovalue = 0.02, blue = holes, red = electrons). $E_g$ = E(HOMO) – E(LUMO).

# 3. Synthesis

### 3.2.1. Compounds prepared via literature procedures

The following compounds were prepared according to literature procedures from commercially available starting materials. For the synthetic pathway and individual procedures for preparation of the aromatic core **8** (Scheme S1), the mesogenic groups **3 - 7** ( Scheme S2) and the emitter **BCzBN** (Scheme 4), please refer to the following chapters :

Table **S1:** Literature known compound prepared for this study analog to the corresponding references.

| | | |
|---|---|---|
| 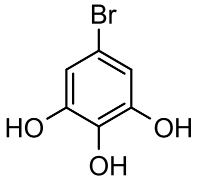 **9** | $C_6H_5BrO_3$ (205.01 g mol$^{-1}$) | Synthesis from 1-bromo-3,4,5-trimethoxybenzene according to Ref. [25].[25] |
| 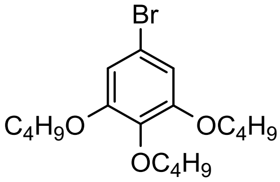 **10** | $C_{18}H_{29}BrO_3$ (373.33 g mol$^{-1}$) | Synthesis from **9** according to Ref. [26].[26] |
| 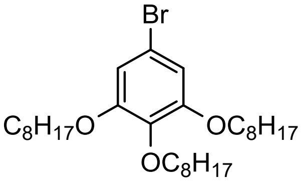 **11** | $C_{30}H_{53}BrO_3$ (541.66 g mol$^{-1}$) | Synthesis from **9** according to Ref. [26].[26] |
| 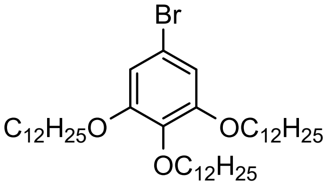 **12** | $C_{42}H_{77}BrO_3$ (709.98 g mol$^{-1}$) | Synthesis from **9** according to Ref. [27].[27] |
| 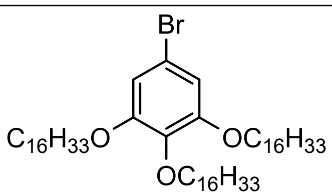 **13** | $C_{54}H_{101}BrO_3$ (878.30 g mol$^{-1}$) | Synthesis from **9** according to Ref. [27].[27] |

| | | |
|---|---|---|
| 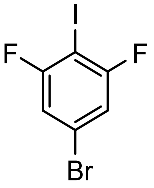 **14** | $C_6H_2BrF_2I$ (318.89 g mol$^{-1}$) | Synthesis from 1-bromo-3,5-difluorobenzene according to Ref. [28].[28] |
| 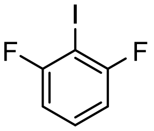 **15** | $C_6H_3F_2I$ (239.99 g mol$^{-1}$) | Synthesis from 1,3,-difluorobenzene according to Ref. [29].[29] |

### 3.2.2. Synthesis of the aromatic core 8

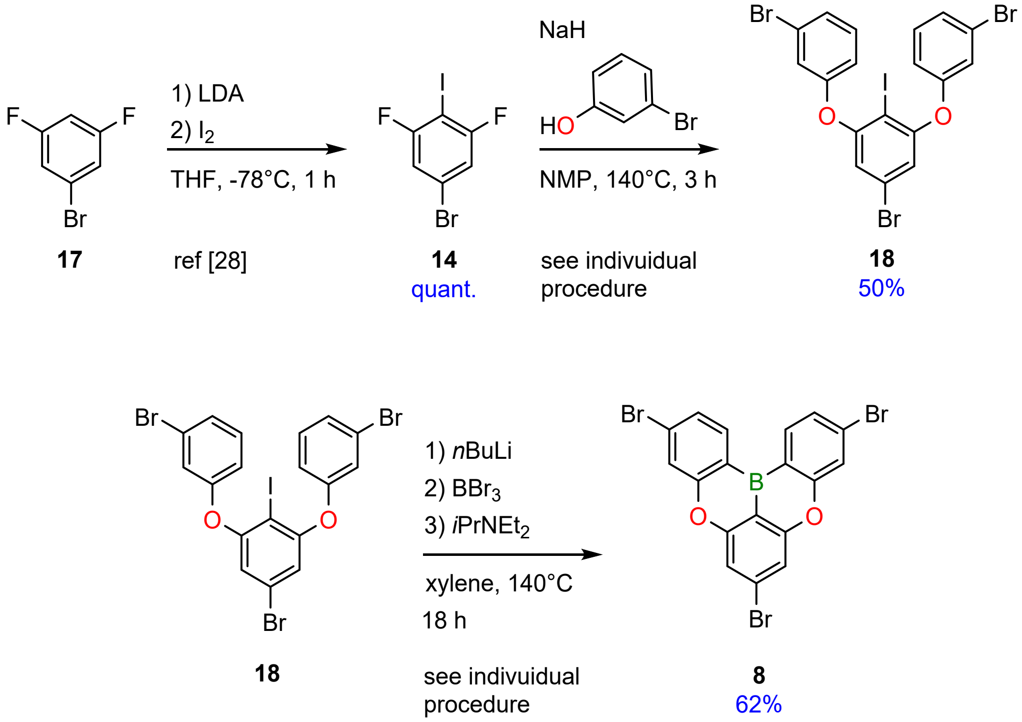


Scheme **S1:** Synthesis of the aromatic core **8**.

**3,3'-((5-Bromo-2-iodo-1,3-phenylene)bis(oxy))bis(bromobenzene) (18):**

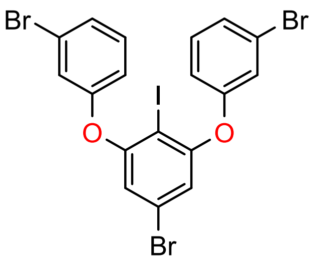

**18**

$C_{18}H_{10}Br_3IO_2$
(624.89 g mol$^{-1}$)

3-Bromophenole (2.7 mL, 25.7 mmol, 2.3 equiv.) was dissolved in NMP (40 mL), added to a flask containing a 60 wt% suspension of NaH in mineral oil (617 mg, 25.7 mmol, 2.3 equiv.) at 0 °C and stirred

at r.t. for 1 h. **14** (3.56 g, 11.2 mmol, 1.0 equiv.) was added as a solution in NMP (10 mL) and the resulting violet reaction mixture was stirred at 140 °C for 3 h. After cooling to r.t., deionized water (120 mL) was added, and the mixture was extracted with EtOAc (3 × 100 mL). The combined organic phase was washed with deionized water (1 × 50 mL), brine (1 × 50 mL) and dried over $MgSO_4$. Removal of the solvent under reduced pressure and column chromatography (silica, d = 3cm, l = 25 cm, gradient: PE : toluene = 40 : 1 to 5 : 1) afforded the desired product as colourless solid (3.5 g, 5.6 mmol, 50%).

$^1$H-NMR (300 MHz, $CDCl_3$): δ = 6.77 (s, 2Hz), 6.98 (ddd, *J* = 8.0 Hz, 2.4 Hz, 1.2 Hz, 2H, 8-H), 7.21 (t, *J* = 2.1 Hz, 2H, 6-H), 7.26 (t, *J* = 8.0 Hz, 2H, 9-H), 7.33 (dd, *J* = 8.2 Hz, 1.2 Hz, 2H, 10-H) ppm.

$^{13}$C-NMR (75 MHz, $CDCl_3$) δ = 82.6 (C-1), 117.5 (C-3), 117.8 (C-6), 122.5 (C-8), 123.33 (C-2), 123.36 (C-5), 127.8 (C-10), 131.3 (C-9), 156.7 (C-7), 158.7 (C-4) ppm.

FT-IR (ATR): $\tilde{\nu}$ = 439 (w), 527 (w), 673(w), 701 (w), 732 (w), 774 (w), 836 (w), 865 (w), 997 (w), 1025 (s), 1061 (w), 1084 (w), 1157 (w), 1205 (s), 1264 (w), 1328 (w), 1389 (s), 1422 (w), 1465 (s), 1557 (s), 1579 (w), 1708 (w), 1928 (w), 2410 (w), 2472 (w), 2672 (w), 2851 (w), 2923 (w), 3066 (w) $cm^{-1}$.

HRMS (EI): calculated for $C_{18}H_{10}Br_3IO_2^+$: 624.7289; found: 624.7296.

**3,7,11-Tribromo-5,9-dioxa-13b-boranaphtho[3,2,1-de]anthracene (8):**

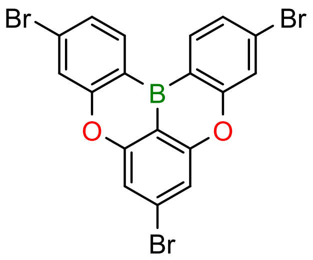


$C_{18}H_8BBr_3O_2$
($503.82\ g\ mol^{-1}$)

**8**

**18** (4.0 g, 6.4 mmol, 1.0 equiv.) was dissolved in xylene (35 mL) and *n*BuLi (2.82 mL, 7.04 mmol, 1.1 equiv., 2.5 M in hexanes) was quickly added at -20 °C. The reaction mixture was warmed up to ambient temperature and stirred for 2 h under formation of a colourless precipitate. $BBr_3$ (729 µL, 7.68 mmol, 1.2 equiv.) was added to the reaction at 0 °C and the precipitate redissolved. After stirring at ambient temperature for 1 h, *i*PrNEt$_2$ (2.39 mL, 14.08 mmol, 2.2 equiv.) was added and the reaction mixture was stirred at 140 °C for 18 h. Aqueous sodium acetate solution (10 mL) and PE (30 mL) were added to quench the reaction and the formed precipitate was treated in an ultrasonic bath (10 min) and filtered off. The filter cake was washed with deionized water, MeOH, EtOH and PE (50 mL each) to afford the product as pale yellow solid (2.0 g, 3.95 mmol, 62%).

$^1$H-NMR (700 MHz, $CDCl_3$): $^1$H-NMR (400 MHz, $CDCl_3$): δ = 7.42 (s, 2H), 7.53 (dd, *J* = 8.2 Hz, 1.8 Hz, 2H), 7.74 (d, *J* = 1.8 Hz, 2H), 8.42 (d, *J* = 8.2 Hz, 2H) ppm.

The spectral data match those reported in the literature. Please note: $^{13}$C-NMR and $^{11}$B-NMR spectra were not recorded due to the low solubility of **8**.[30]

### 3.2.3. Synthesis of the pinacolborolanes 3 - 7

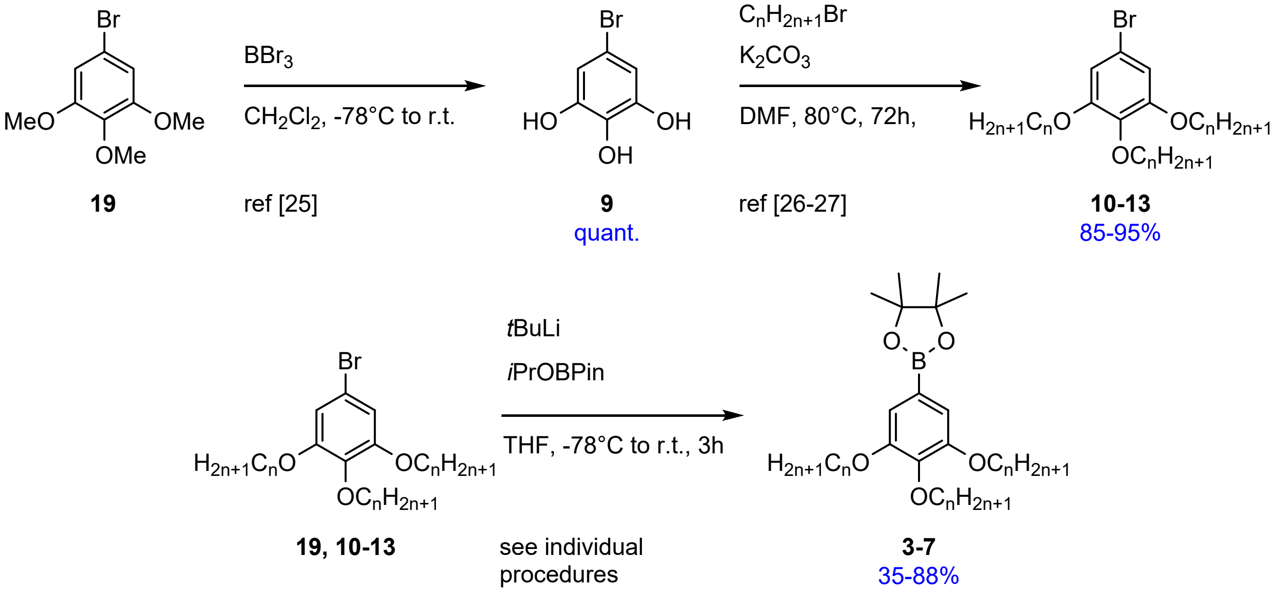


Scheme **S2:** Synthesis of the pinacolborolanes **3** – **7**.

**General procedure for preparation of the pinacolborolanes 3 – 7:**

The respective alkoxy bromobenzene **19**, **10 - 13** (1.0 equiv.) was dissolved in anhydrous THF (80 mL) and cooled to -78 °C before *t*BuLi (2.0 equiv.) was added dropwise. The mixture was stirred at -78 °C for 5 min, quickly warmed to r.t. (water bath) and cooled back to -78 °C before *i*PrOBPin (1.3 equiv.) was added. The reaction was stirred at r.t for 33 × 50 mL1 × 50 mLcolumn chromatography on silica gel (column dimensions and eluent specified in each procedure) and filtration over a silica gel plug (eluent specified in each procedure) yielded the products **3 - 7** as colorless solids.**4,4,5,5-Tetramethyl-2-(3,4,5-trimethoxyphenyl)-1,3,2-dioxaborolane (3):**

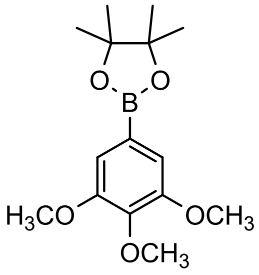


$C_{15}H_{23}BO_5$
(294.15 g $mol^{-1}$)

**3**

**Conditions:** According to general procedure: Bromobenzene **19** (2.17 g, 8.78 mmol, 1.0 equiv.), (10.33 mL, 17.56 mmol, 2.0 equiv., 1.7 M in pentane) and *i*PrOBPin (2.33 mL, 11.42 mmol, 1.3 equiv.) in anhydrous THF (80 mL).

**Purification:** Column chromatography on silica gel (d = 3 cm, l = 10 cm, gradient: PE : EtOAc = eluent: PE : EtOAc = 3 : 1) and filtration over a silica gel plug (eluent: PE : EtOAc = 3 : 1).

**Yield:** Colorless solid (2.27 g, 7.72 mmol, 88%).

$^{1}$H-NMR (400 MHz, $CDCl_3$): δ = 1.34 (s, 12H), 3.87 (s, 3H), 3.90 (s, 6H), 7.03 (s, 2H) ppm.

$^{13}$C-NMR (101 MHz, $CDCl_3$) δ = 25.0, 56.3, 60.9, 84.0, 105.4, 111.5, 141.0, 153.1 ppm.

$^{11}$B-NMR (128 MHz, $CDCl_3$): δ = 30.4 ppm.

The spectral data match those reported in the literature.[31]

**4,4,5,5-Tetramethyl-2-(3,4,5-tributoxyphenyl)-1,3,2-dioxaborolane (4):**

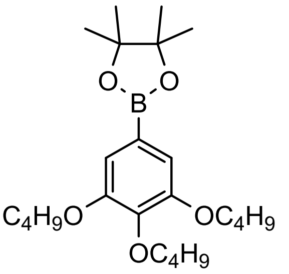


$C_{24}H_{41}BO_5$
(420.40 g $mol^{-1}$)

**4**

**Conditions:** According to general procedure: Bromobenzene **10** (3.40 g, 9.11 mmol, 1.0 equiv.), *t*BuLi (10.71 mL, 18.21 mmol, 2.0 equiv., 1.7 M in pentane) and *i*PrOBPin (2.42 mL, 11.84 mmol, 1.3 equiv.) in anhydrous THF (90 mL).

**Purification:** Column chromatography on silica gel (d = 3 cm, l = 15 cm, eluent: PE : EtOAc = 20 : 1) and filtration over a silica gel plug (eluent: PE : EtOAc = 10 : 1).

**Yield:** Colorless solid (2.03 g, 4.83 mmol, 53%).

$^1$H-NMR (400 MHz, $CDCl_3$): δ = 0.92–1.00 (m, 9H), 1.33 (s, 12H), 1.43–1.55 (m, 6H), 1.66–1.85 (m, 6H), 3.86–4.22 (m, 6H), 7.00 (s, 2H) ppm.

$^{13}$C-NMR (101 MHz, $CDCl_3$) δ = 14.0, 14.0, 19.3, 19.4, 25.0, 31.7, 32.5, 68.9, 73.1, 83.9, 113.0, 141.3, 153.1 ppm.

$^{11}$B-NMR (128 MHz, $CDCl_3$): δ = 31.4 ppm.

Please note: A $^{13}$C signal for the carbon atom *ipso* to the boron atom was not observed due to signal broadening. The spectral data match those reported in the literature.[32]

**4,4,5,5-Tetramethyl-2-(3,4,5-tris(octyloxy)phenyl)-1,3,2-dioxaborolane (5):**

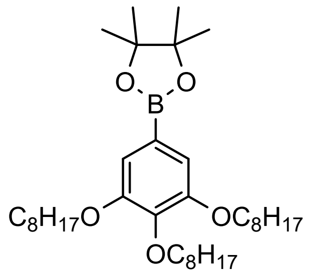


$C_{36}H_{65}BO_5$
(588.72 g $mol^{-1}$)

**5**

**Conditions:** According to general procedure: Bromobenzene **11** (5.53 g, 10.21 mmol, 1.0 equiv.), *t*BuLi (12.01 mL, 20.42 mmol, 2.0 equiv., 1.7 M in pentane) and *i*PrOBPin (2.71 mL, 13.27 mmol, 1.3 equiv.) in anhydrous THF (60 mL) and anhydrous $Et_2O$ (40 mL).

**Purification:** Column chromatography on silica gel (d = 3 cm, l = 15 cm, gradient: PE : EtOAc = 40 : 1 to 20 : 1) and filtration over a silica gel plug (eluent: PE : EtOAc = 10 : 1).

**Yield:** Colorless solid (3.32 g, 5.44 mmol, 55%).

$^1$H-NMR (400 MHz, $CDCl_3$): δ = 0.78–0.94 (m, 9H), 1.22–1.40 (m, 36H), 1.40–1.52 (m, 6H), 1.67–1.88 (m, 6H), 3.90–4.12 (m, 6H), 6.99 (s, 2H) ppm.

$^{13}$C-NMR (101 MHz, $CDCl_3$) δ = 14.2, 22.8, 22.8, 25.0, 26.3, 26.3, 29.4, 29.5, 29.6, 29.7, 30.5, 32.0, 32.1, 69.2, 73.5, 83.9, 113.0, 141.3, 153.0 ppm.

$^{11}$B-NMR (128 MHz, $CDCl_3$): δ = 32.5 ppm.

Please note: A $^{13}$C signal for the carbon atom *ipso* to the boron atom was not observed due to signal broadening. The spectral data match those reported in the literature.[25]

**4,4,5,5-Tetramethyl-2-(3,4,5-tris(dodecyloxy)phenyl)-1,3,2-dioxaborolane (6):**

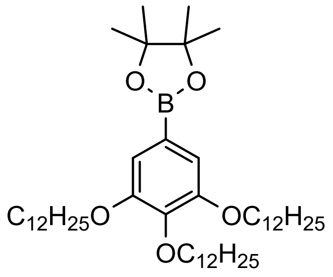

$C_{48}H_{89}BO_5$
(757.04 g mol$^{-1}$)

**6**

**Conditions:** According to general procedure: Bromobenzene **12** (7.45 g, 10.49 mmol, 1.0 equiv.), *t*BuLi (12.35 mL, 20.99 mmol, 2.0 equiv., 1.7 M in pentane) and *i*PrOBPin (2.79 mL, 13.64 mmol, 1.3 equiv.) in anhydrous THF (60 mL) and anhydrous $Et_2O$ (40 mL) with lithiation at -20 °C due to solubility issues.

**Purification:** Column chromatography on silica gel (d = 3 cm, l = 15 cm, gradient: PE : EtOAc = 40 : 1 to 20 : 1) and filtration over a silica gel plug (eluent: PE : EtOAc = 10 : 1).

**Yield:** Colorless solid (2.81 g, 3.71 mmol, 35%).

$^1$H-NMR (400 MHz, $CDCl_3$): δ = 0.78–0.97 (m, 9H), 1.21–1.39 (m, 60H), 1.42–1.52 (m, 6H), 1.65–1.93 (m, 6H), 3.50–4.81 (m, 6H), 6.99 (s, 2H) ppm.

$^{13}$C-NMR (101 MHz, $CDCl_3$) δ = 14.3, 22.8, 25.0, 26.3, 29.5, 29.5, 29.6, 29.7, 29.8, 29.8, 29.8, 29.9, 29.9, 29.9, 30.5, 32.1, 32.1, 69.2, 73.5, 83.9, 113.0, 141.3, 153.0 ppm.

$^{11}$B-NMR (128 MHz, $CDCl_3$): δ = 35.3 ppm.

Please note: A $^{13}$C signal for the carbon atom *ipso* to the boron atom was not observed due to signal broadening. The spectral data match those reported in the literature.[25]

**4,4,5,5-Tetramethyl-2-(3,4,5-tris(dodecyloxy)phenyl)-1,3,2-dioxaborolane (7):**

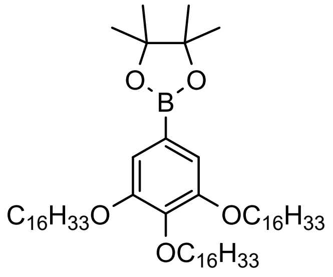

$C_{60}H_{113}BO_5$
(925.37 g mol$^{-1}$)

**7**

**Conditions:** According to general procedure: Bromobenzene **13** (1.40 g, 1.59 mmol, 1.0 equiv.), *t*BuLi (1.88 mL, 3.19 mmol, 2.0 equiv., 1.7 M in pentane) and *i*PrOBPin (0.34 mL, 2.07 mmol, 1.3 equiv.) in anhydrous THF (60 mL) with lithiation at -20 °C due to solubility issues.

**Purification:** Column chromatography on silica gel (d = 3 cm, l = 15 cm, gradient: PE : EtOAc = 20 : 1).

**Yield:** Colorless solid (670 mg, 724 µmol, 45%).

$^{1}$H-NMR (400 MHz, $CDCl_3$): δ = 0.73–1.05 (m, 9H), 1.17–1.58 (m, 84H), 1.68–1.84 (m, 6H), 3.63–4.55 (m, 6H), 7.00 (s, 2H) ppm.

$^{13}$C-NMR (101 MHz, $CDCl_3$): δ = 14.3, 22.8, 25.0, 26.3, 29.5, 29.6, 29.7, 29.8, 29.8, 29.9, 29.9, 29.9, 30.5, 32.1, 69.2, 73.5, 83.9, 112.9, 141.3, 153.0 ppm.

$^{11}$B-NMR (128 MHz, $CDCl_3$): δ = 30.5 ppm.

Please note: A $^{13}$C signal for the carbon atom *ipso* to the boron atom was not observed due to signal broadening. The spectral data match those reported in the literature.[33]

### 3.2.4. Synthesis of LC1 – LC16:

**Preparation of LC1 – LC16 (GP2):**

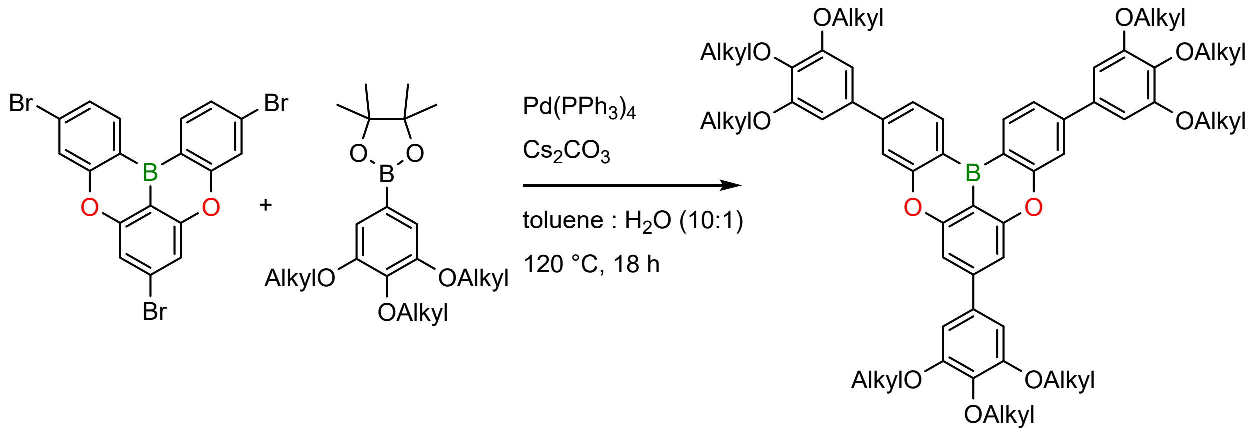


**8** **3-7** **LC1 - LC16**

38-51%

Scheme **S3:** Synthesis of the **LCn** series.

The aromatic core **8** (1.0 equiv), the respective pinacol borolane **3 - 7** (3.5 equiv.), $Pd(PPh_3)_4$ (0.15 equiv.) and $Cs_2CO_3$ (5.0 equiv.) were suspended in a mixture of degassed toluene and degassed water. The mixture was heated to 120 °C for 18 h, cooled to ambient temperature and filtered over a pad of borylated silica gel (d = 2 cm, l = 5 cm, eluent: specified in each procedure). Recrystallization (conditions specified in the individual procedures) yielded **LC1 – LC16** as pale-yellow solids or waxes.

**3,7,11-Tris(3,4,5-trimethoxyphenyl)-5,9-dioxa-13b-boranaphtho[3,2,1-de]anthracene (LC1):**

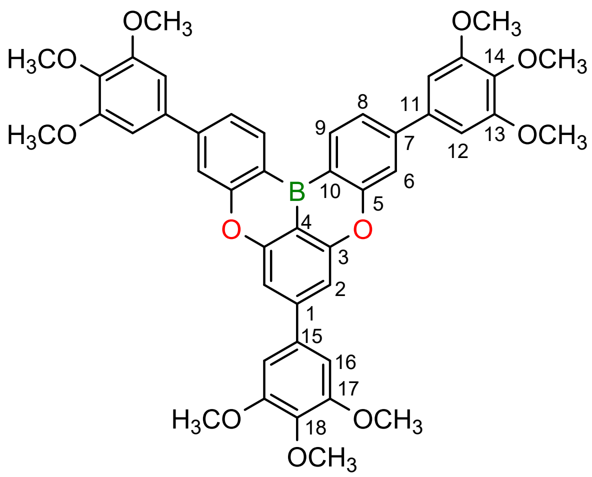


**LC1**

$C_{45}H_{41}BO_{11}$
(768.62 g $mol^{-1}$)

**Conditions:** According to **GP3**: Aromatic core **8** (150 mg, 296 µmol, 1.0 equiv.), pinacol borolane **3** (305 mg, 1.04 mmol, 3.5 equiv.), $Pd(PPh_3)_4$ (51 mg, 44 µmol, 0.15 equiv.) and $Cs_2CO_3$ (482 mg, 1.48 mmol, 5.0 equiv.) in degassed toluene (15 mL) and $H_2O$ (1 mL).

**Purification:** Filtration over a plug of borylated silica gel (eluent: $CHCl_3$) followed by recrystallization from toluene.

**Yield:** Pale yellow solid (99 mg, 129 µmol, 44%).

$^{1}$H-NMR (700 MHz, $CDCl_3$): δ = 3.93–3.96 (m, 9H, $OCH_3$), 3.98–4.01 (m, 18H, $OCH_3$), 6.91–7.07 (m, 6H, 12-H, 16-H), 7.47 (s, 2H, 2-H), 7.63 (dd, *J* = 7.9 Hz, 1.8 Hz, 2H, 8-H), 7.76 (s, 2H, 6-H), 8.74 (d, *J* = 7.9 Hz, 2H, 9-H) ppm.

$^{13}$C-NMR (176 MHz, $CDCl_3$) δ = 56.40, 56.43, 61.2($OCH_3$), 104.7 (C-16), 104.9 (C-12), 107.4 (C-2), 113.9 (C-4), 116.5 (C-6), 121.4 (C-10), 122.0 (C-8), 135.1 (C-9), 136.0 (C-7 or C-11), 136.3 (C-1 or C-15), 138.6 (C-18), 138.7 (C-14), 146.7 (C-7 or C-11), 148.2 (C-1 or C-15), 153.8 (C-17), 153.8 (C-13), 157.9 (C-3), 161.2 (C-5) ppm.

FT-IR (ATR): $\tilde{\nu}$ = 3074 (w), 2995 (w), 2936 (w), 1630 (s), 1610 (s), 1586 (s), 1566 (s), 1543 (s), 1510 (s), 1463 (s), 1399 (s), 1329 (w), 1292 (w), 1242 (w), 1184 (w), 1162 (w), 1127 (s), 1069 (w), 1007 (w), 891 (w), 818 (w), 769 (w), 730 (w), 714 (w), 688 (w), 612 (w), 529 (w), 463 (w), 441 (w), 426 (w), 409 (w) $cm^{-1}$.

HRMS (ESI, $[M+H]^+$): calculated for $C_{75}H_{97}BO_{11}^{+}$: 769.2822; found: 769.2820.

Please note: A $^{11}$B NMR signal was not observed presumably due to signal broadening resulting from the chemical environment of boron embedded aromatics.

**3,7,11-Tris(3,4,5-tributoxyphenyl)-5,9-dioxa-13b-boranaphtho[3,2,1-de]anthracene (LC4):**

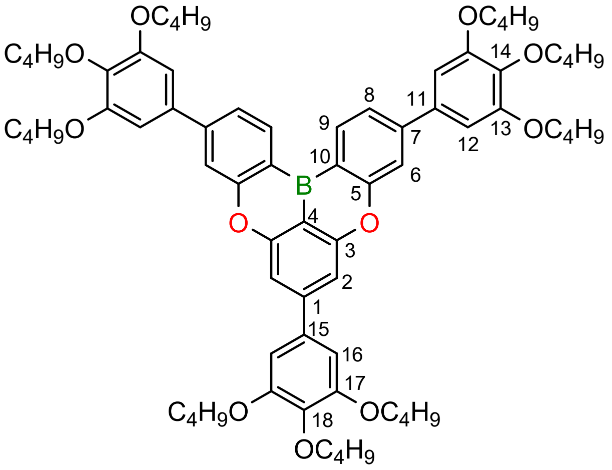


**LC4**

$C_{72}H_{95}BO_{11}$
(1147.35 g mol$^{-1}$)

**Conditions:** According to **GP3**: Aromatic core **8** (275 mg, 543 µmol, 1.0 equiv.), pinacol borolane **4** (798 mg, 1.90 mmol, 3.5 equiv.), $Pd(PPh_3)_4$ (94 mg, 82 µmol, 0.15 equiv.) and $Cs_2CO_3$ (884 mg, 2.71 mmol, 5.0 equiv.) in degassed toluene (18 mL) and $H_2O$ (2 mL).

**Purification:** Filtration over a plug of borylated silica gel (eluent: toluene : $CHCl_3$ = 1 : 2) followed by recrystallization from a mixture of EtOH : acetone (2:1) and EtOH : acetone (1:1).

**Yield:** Pale yellow solid (320 mg, 279 µmol, 51%).

$^1$H-NMR (700 MHz, $CDCl_3$): δ = 0.92–1.18 (m, 27H, $CH_3$), 1.51–1.64 (m, 18H, CH2), 1.74–1.82 (m, 6H, $OCH_2CH_2$), 1.82–1.93 (m, 12H, $OCH_2CH_2$), 4.02–4.09 (m, 6H, 19-$OCH_2$, 20-$OCH_2$), 4.09–4.14 (m, 12H, 13-$OCH_2$, 14-$OCH_2$), 6.94–7.07 (m, 6H, 16-H), 7.46 (s, 2H, 2-H), 7.62 (dd, *J* = 8.2 Hz, 1.8 Hz, 2H, 8-H), 7.75 (d, *J* = 1.8 Hz, 2H, 6-H),), 8.72 (d, *J* = 8.2 Hz, 2H, 9-H) ppm.

$^{13}$C-NMR (176 MHz, $CDCl_3$) δ = 13.86, 13.89, 19.2, 19.3, 31.5, 32.3 ($CH_2$), 68.9, 69.0, 73.2 ($OCH_2$), 106.0 (C-16), 106.1 (C-12), 107.1 (C-2), 113.6 (C-4), 116.1 (C-6), 121.1 (C-10), 121.8 (C-8), 134.9 (C-9), 135.2 (C-7 or C-11), 135.5 (C-1 or C-15), 138.71 (C-18), 138.74 (C-14), 146.6 (C-7 or C-11), 148.2 (C-1 or C-15), 153.5 (C-17), 153.5 (C-13), 157.7 (C-3), 161.0 (C-5) ppm.

FT-IR (ATR): $\tilde{\nu}$ = 2956 (s), 2932 (s), 2871 (s), 1630 (w), 1609 (w), 1585 (w), 1563 (w), 1542 (w), 1506 (w), 1466 (w), 1401 (s), 1380 (w), 1327 (w), 1293 (w), 1197 (ws), 1179 (w), 1165 (w), 1108 (s), 1066 (w), 1023 (w), 1000 (w), 956 (w), 898 (w), 827 (w), 811 (w), 763 (w), 740 (w), 720 (w), 685 (w), 611 (w), 450 (w) cm$^{-1}$.

HRMS (ESI, $[M+H]^+$): calculated for $C_{75}H_{96}BO_{11}^+$: 1147.7052; found: 1147.7057.

Please note: A $^{11}$B NMR signal was not observed presumably due to signal broadening resulting from the chemical environment of boron embedded aromatics.

**3,7,11-Tris(3,4,5-tris(octyloxy)phenyl)-5,9-dioxa-13b-boranaphtho[3,2,1-de]anthracen (LC8):**

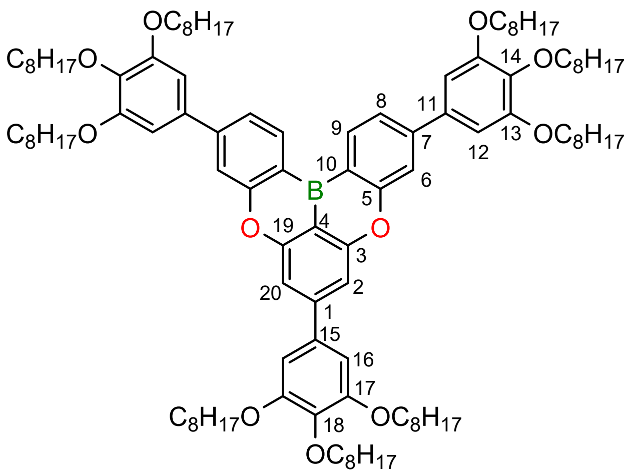


**LC8**

**Conditions:** According to **GP3**: Aromatic core **8** (70 mg, 138 µmol, 1.0 equiv.), pinacol borolane **5** (285 mg, 483 µmol, 3.5 equiv.), $Pd(PPh_3)_4$ (24 mg, 21 µmol, 0.15 equiv.) and $Cs_2CO_3$ (225 mg, 691 µmol, 5.0 equiv.) in degassed toluene (15 mL) and $H_2O$ (1 mL).

**Purification:** Filtration over a plug of borylated silica gel (eluent: toluene) followed by recrystallization from acetone.

**Yield:** Pale yellow wax (100 mg, 60 µmol, 43%).

$^{1}$H-NMR (700 MHz, $CDCl_3$): δ = 0.78–1.06 (m, 27H, $CH_3$), 1.24–1.47 (m, 72H, $CH_2$), 1.48–1.58 (m, 18H, $CH_2$), 1.76–1.85 (m, 6H, $OCH_2CH_2$), 1.85–1.91 (m, 12H, $OCH_2CH_2$), 4.02–4.07 (m, 6H, 19-$OCH_2$, 20-$OCH_2$), 4.08–4.14 (m, 12H, 13-$OCH_2$, 14-$OCH_2$), 6.87–7.14 (m, 6H, 12-H, 16-H), 7.47 (s, 2H, 2-H), 7.62 (d, $J$ = 8.0 Hz, 2H, 8-H), 7.75 (s, 2H, 6-H), 8.73 (d, $J$ = 8.0 Hz, 2H, 9-H) ppm.

$^{13}$C-NMR (176 MHz, $CDCl_3$) δ = 14.26, 14.27 ($CH_3$), 22.8, 22.9, 26.3, 29.46, 29.54, 29.55, 29.61, 29.7, 30.5, 32.0, 32.1 ($CH_2$),69.4, 69.5, 73.8 ($OCH_2$), 106.2 (C-16), 106.3 (C-12), 107.2 (C-2), 113.8 (C-4), 116.3 (C-6), 121.2 (C-10), 121.9 (C-8), 135.0 (C-9), 135.3 (C-7 or C-11), 135.7 (C-1 or C-15), 138.90 (C-18), 138.92 (C-14), 146.8 (C-7 or C-11), 148.3 (C-1 or C-15), 153.7 (C-17), 153.7 (C-13), 157.9 (C-3), 161.2 (C-5) ppm.

FT-IR (ATR): $\nu$ = 2954 (w), 2921 (s), 2853 (s), 1630 (w), 1609 (s), 1584 (w), 1564 (w), 1542 (w), 1507 (w), 1466 (w), 1401 (s), 1380 (s), 1328 (s), 1293 (w), 1241 (w), 1197 (w), 1165 (w), 1112 (s), 1067 (w), 1013 (w), 955 (w), 893 (w), 811 (w), 763 (w), 722 (w), 689 (w), 609 (w), 446 (w), 421 (w) cm$^{1}$.

HRMS (ESI, $[M+H]^+$): calculated for $C_{108}H_{168}BO_{11}^+$: 1653.2703; found: 1653.2717.

Please note: A $^{11}$B NMR signal was not observed presumably due to signal broadening resulting from the chemical environment of boron embedded aromatics.

**3,7,11-Tris(3,4,5-tris(dodecyloxy)phenyl)-5,9-dioxa-13b-boranaphtho[3,2,1-de]anthracene (LC12):**

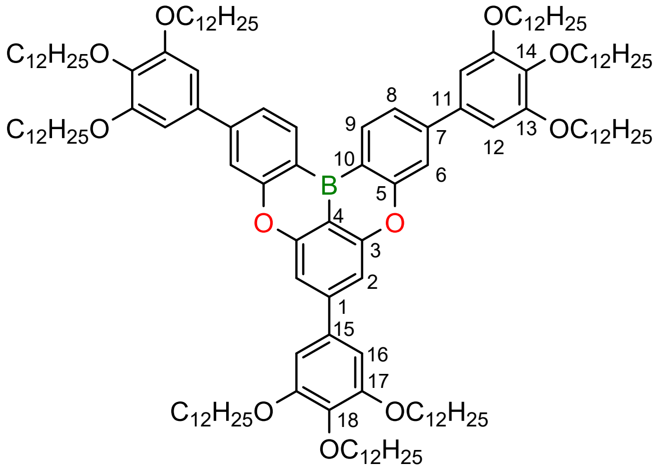


LC12

$C_{144}H_{239}BO_{11}$
(2157.30 g mol$^{-1}$)

**Conditions:** According to **GP3**: Aromatic core **8** (60 mg, 118 µmol, 1.0 equiv.), pinacol borolane **6** (284 mg, 414 µmol, 3.5 equiv.), $Pd(PPh_3)_4$ (21 mg, 18 µmol, 0.15 equiv.) and $Cs_2CO_3$ (193 mg, 592 µmol, 5.0 equiv.) in degassed toluene (15 mL) and $H_2O$ (1 mL).

**Purification:** Filtration over a plug of borylated silica gel (eluent: toluene) followed by recrystallization from a mixture of acetone and EtOAc (v : v = 1 : 1).

**Yield:** Pale yellow wax (110 mg, 51 µmol, 43%).

$^1$H-NMR (700 MHz, $CDCl_3$): δ = 0.80–1.01 (m, 33H), 1.13–1.46 (m, 101H), 1.48–1.56 (m, 19H), 1.74–1.84 (m, 7H), 1.84–1.92 (m, 13H), 4.00–4.07 (m, 7H), 4.08–4.14 (m, 15H), 6.92–7.08 (m, 7H), 7.46 (s, 2H), 7.62 (d, $J$ = 8.0 Hz, 2H), 7.75 (s, 2H), 8.73 (d, $J$ = 8.0 Hz, 2H) ppm.

$^{13}$C-NMR (176 MHz, $CDCl_3$) δ = 14.26, 14.27 ($CH_3$), 22.8, 22.9, 26.29, 26.31, 29.52, 29.56, 29.59, 29.62, 29.79, 29.81, 29.82, 29.87, 29.91, 29.93, 30.5, 32.07, 32.10 ($CH_2$), 69.43, 69.47, 73.8 ($OCH_2$), 106.2 (C-16), 106.3 (C-12), 107.2 (C-2), 113.8 (C-4), 116.3 (C-6), 121.2 (C-10), 122.0 (C-8), 135.0 (C-9), 135.4 (C-7 or C-11), 135.7 (C-1 or C-15), 138.9 (C-18), 138.9 (C-14), 146.8 (C-7 or C-11), 148.4 (C-1 or C-15), 153.7 (C-17), 153.7 (C-13), 157.9 (C-3), 161.2 (C-5) ppm.

FT-IR (ATR): $\nu$ = 2919 (s), 2851 (s), 1630 (w), 1610 (w), 1585 (w), 1564 (w), 1542 (w), 1507 (w), 1466 (w), 1401 (s), 1381 (w), 1328 (s), 1293 (w), 1242 (w), 1197 (w), 1164 (w), 1114 (s), 1068 (w), 1006 (w), 894 (w), 811 (w), 763 (w), 720 (w), 610 (w) cm$^{-1}$.

HRMS (ESI, [M+H]$^+$): calculated for $C_{144}H_{23110b}O_{11}^+$: 2158.8387; found: 2157.8354.

Please note: A $^{11}$B NMR signal was not observed presumably due to signal broadening resulting from the chemical environment of boron embedded aromatics.

**3,7,11-Tris(3,4,5-tris(hexadecyloxy)phenyl)-5,9-dioxa-13b-boranaphtho[3,2,1-de]anthracene (LC16):**

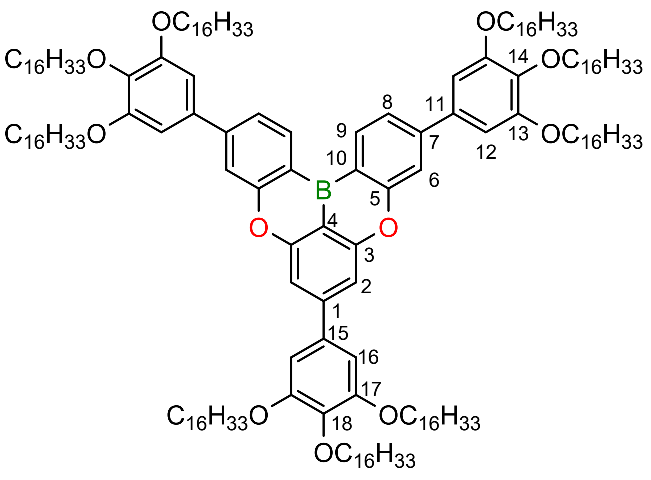


$C_{180}H_{311}BO_{11}$
(2662.27 g mol$^{-1}$)

**Conditions:** According to **GP3**: Aromatic core **8** (50 mg, 99 µmol, 1.0 equiv.), pinacol borolane **7** (320 mg, 345 µmol, 3.5 equiv.), $Pd(PPh_3)_4$ (17 mg, 15 µmol, 0.15 equiv.) and $Cs_2CO_3$ (161 mg, 493 µmol, 5.0 equiv.) in degassed toluene (10 mL) and $H_2O$ (1 mL).

**Purification:** Filtration over a plug of silica gel (eluent: toluene) followed by twofold recrystallization from EtOAc.

**Yield:** Pale yellow solid (100 mg, 38 µmol, 38%).

$^{1}$H-NMR (700 MHz, $CDCl_3$): δ = 0.81–0.94 (m, 27H, $CH_3$), 1.19–1.45 (m, 216H, $CH_2$), 1.48–1.57 (m, 18H, $CH_2$), 1.76–1.83 (m, 6H, $OCH_2C\mathit{H}_2$), 1.84–1.92 (m, 12H, $OCH_2C\mathit{H}_2$), 4.02–4.07 (m, 6H, 19-$OCH_2$, 20-$OCH_2$), 4.08–4.14 (m, 16H, 13-$OCH_2$, 14-$OCH_2$), 6.92–6.99 (m, 6H, 12-H, 16-H), 7.46 (s, 2H, 2-H), 7.62 (d, $J$ = 8.2 Hz, 2H, 8-H), 7.75 (s, 2H, 6-H), 8.73 (d, $J$ = 8.2 Hz, 2H, 9-H) ppm.

$^{13}$C-NMR (176 MHz, $CDCl_3$) δ = 14.3 ($CH_3$), 22.84, 22.85, 26.30, 26.31, 29.52, 29.54, 29.60, 29.62, 29.79, 29.82, 29.84, 29.87, 29.88, 29.90, 29.90, 29.92, 29.94, 30.5, 32.07, 32.09 ($CH_2$), 69.4, 69.5, 73.8 ($OCH_2$), 106.2 (C-16), 106.3 (C-12), 107.2 (C-2), 113.8 (C-4), 116.3 (C-6), 121.2 (C-10), 122.0 (C-8), 135.0 (C-9), 135.4 (C-7 or C-11), 135.7 (C-1 or C-15), 138.90 (C-18), 138.93 (C-14), 146.8 (C-7 or C-11), 148.4 (C-1 or C-15), 153.68 (C-17), 153.70 (C-13), 157.9 (C-3), 161.2 (C-5) ppm.

FT-IR (ATR): $\tilde{\nu}$ =2916 (s), 2849 (s), 1631 (w), 1611 (w), 1585 (w), 1566 (w), 1542 (w), 1509 (w), 1467 (w), 1403 (w), 1383 (w), 1331 (w), 1295 (w), 1245 (w), 1199 (w), 1165 (w), 1118 (w), 1070 (w), 1014 (w), 908 (w), 812 (w), 721 (w), 610 (w) cm$^{-1}$.

HRMS (LIFDI, $[M]^+$): calculated for $C_{180}H_{311}BO_{11}^{+}$: 2661.3912, found: 2661.3898.

Please note: A $^{11}$B NMR signal was not observed presumably due to signal broadening resulting from the chemical environment of boron embedded aromatics.

### 3.2.5. Synthesis of the emitter BCzBN

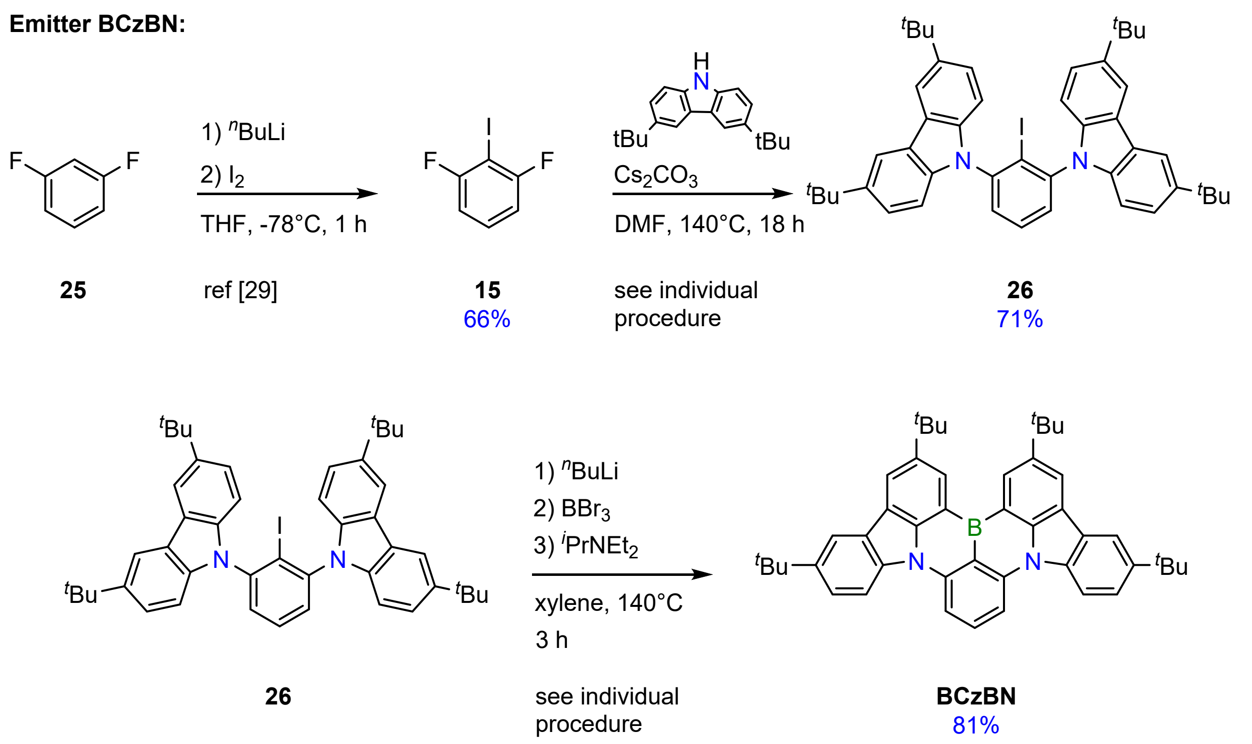


Scheme **4:** Synthesis of the emitter **BCzBN** starting from commercially available difluorobenzene **25**.

**9,9'-(2-Iodo-1,3-phenylene)bis(3,6-di-tert-butyl-9H-carbazole) (26):**

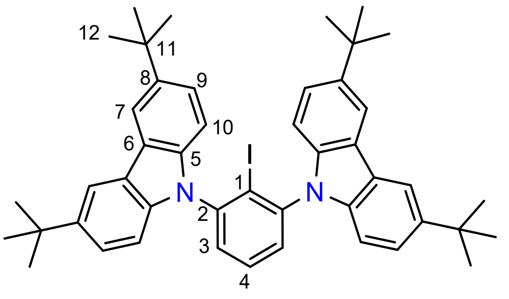

$C_{22}H_{37}BO_4$
(376.34 g mol$^{-1}$)

**JKN_266**

The fluoro precursor **15** (490 mg, 2.04 mmol, 1.0 equiv.), the di-*tert*butylcarbazole (1.2 g, 4.29 mmol, 2.1 equiv.) and oven dried (80 °C) $Cs_2CO_3$ (2.0 g, 6.13 mmol, 3.0 equiv.) were stirred in anhydrous DMF (20 mL) at 140 °C for 18 h. The reaction was quenched by addition of deion. $H_2O$ (50 mL) and the mixture was extracted with EtOAc (3 × 75 mL). The combined extracts were washed with brine (1 × 50 mL) and dried over $MgSO_4$. Removal of the volatiles under reduced pressure and subsequent column chromatography on silica gel (d = 2 cm, l = 15 cm, Eluent = PE : EtOAc = 50 : 1) afforded the desired product **26** as colorless solid (1.1 g, 1.45 mmol, 71%).

$^1$H-NMR (500 MHz, $CDCl_3$): δ = 1.48 (s, 36H, 7-H), 7.09 (d, *J* = 8.5 Hz, 4H, 10-H), 7.51 (dd, *J* = 8.5 Hz, 1.7 Hz, 4H, 9-H), 7.55 (d, *J* = 7.8 Hz, 2H, 3-H), 7.70 (t, *J* = 7.8 Hz, 1H, 4-H), 8.17 (d, *J* = 1.7 Hz, 4H, 7-H) ppm.

$^{13}$C-NMR (126 MHz, $CDCl_3$) δ = 32.2 (C-12), 34.9 (C-11), 105.6 (C-1), 109.6 (C-10), 116.6 (C-7), 123.4 (C-6), 123.9 (C-9), 130.6 (C-3), 130.7 (C-4), 139.3 (C-5), 143.2 (C-8), 143.7 (C-2) ppm.

FT-IR (ATR) $\tilde{\nu}$ = 3047.23 (w), 2959.58 (w), 2903.20 (w), 2866.26 (w), 1609.80 (w), 1569.59 (w), 1477.63 (w), 1392.72 (w), 1363.04 (w), 1323.61 (w), 1294.25 (w), 1262.59 (w), 1236.40 (w), 1201.62 (w), 1166.63 (w), 1137.26 (w), 1104.00 (w), 1033.82 (w), 908.51 (w), 877.71 (w), 840.22 (w), 806.79 (w), 726.93 (w), 694.57 (w), 679.52 (w), 648.79 (w), 612.35 (w) $cm^{-1}$.

HRMS (EI) *m/z* for $C_{46}H_{51}IN_2$ calculated: 758.3091 $[MH]^+$, found: 758.3096

**BCzBN:**

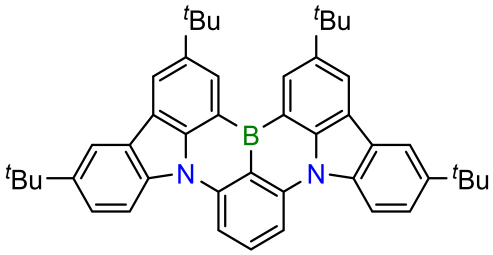


$C_{46}H_{49}BN_2$
(640.72 g $mol^{-1}$)

**BCzBN**

The iodo precursor **26** (190 mg, 250 µmol, 1.0 equiv.) was dissolved in anhydrous Xylene (5 mL) and *n*BuLi (120 µL, 300 µmol, 1.2 equiv., 2.5 M in hexanes) were quickly added at 0 °C. The mixture was stirred at r.t. for 2 h before $BBr_3$ (32 µL, 328 µmol, 1.3 equiv.) was added. After stirring at r.t., for 15 min diisopropylethylamine (91 µL, 525 µmol, 2.1 equiv.) was added and the mixture was stirred at 140 °C for 3 h. Saturated aqueous NaOAc solution (20 mL) was added to stop the reaction and the mixture was extracted with $CH_2Cl_2$ (3 × 15 mL). The combined extracts were washed with brine (1 × 20 mL), dried over $MgSO_4$ and concentrated under reduced pressure to yield the crude product as yellow solid. Dissolution of the crude product in $CHCl_3$ (2 mL), precipitation by addition of EtOH (5 mL) and subsequent filtration yielded the final product **BCzBN** as bright yellow powder (130 mg, 203 µmol, 81%).

$^1$H-NMR (400 MHz, $CDCl_3$): δ = 1.54 (s, 18H), 1.68 (s, 18H), 7.66 (dd, *J* = 8.8 Hz, 2.1 Hz, 2H), 8.00 (t, *J* = 8.3 Hz, 1H), 8.27 (d, *J* = 2.1 Hz, 2H), 8.32 (d, *J* = 8.4 Hz, 2H), 8.39 (d, *J* = 8.8 Hz, 2H), 8.47 (d, *J* = 1.8 Hz, 2H), 9.13 (d, *J* = 2.0 Hz, 2H) ppm.

$^{13}$C-NMR (101 MHz, $CDCl_3$): δ = 32.0, 32.4, 34.9, 35.3, 108.1, 114.2, 117.4, 120.8, 123.8, 124.5, 127.2, 130.0, 133.2, 138.5, 141.7, 144.4, 144.8, 145.4 ppm.

The spectroscopic data were in good agreement with the literature.[34]

Please note: A $^{11}$B NMR signal and the $^{13}$C signals of carbons ipso to the B atom were not observed presumably due to signal broadening resulting from the chemical environment of boron embedded aromatics.

# 4. NMR/ HRMS spectra

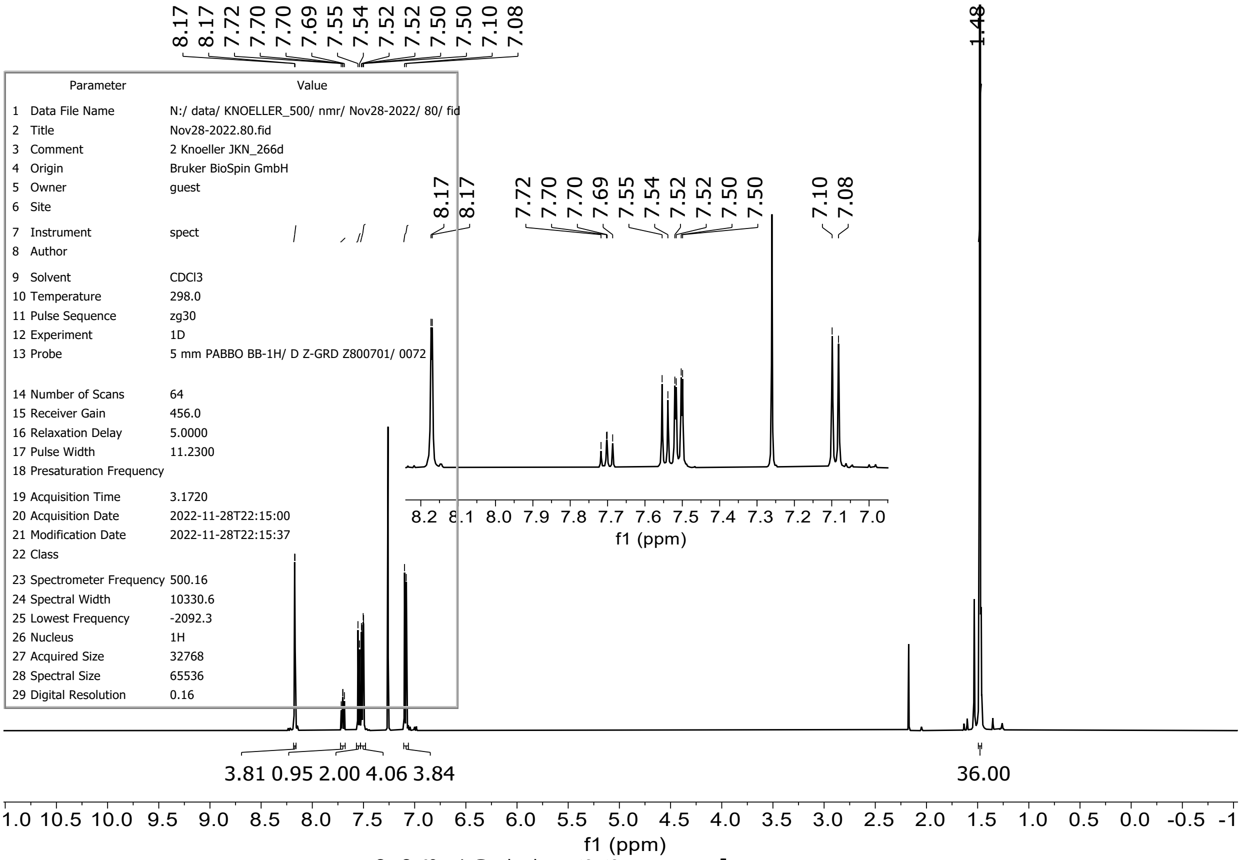


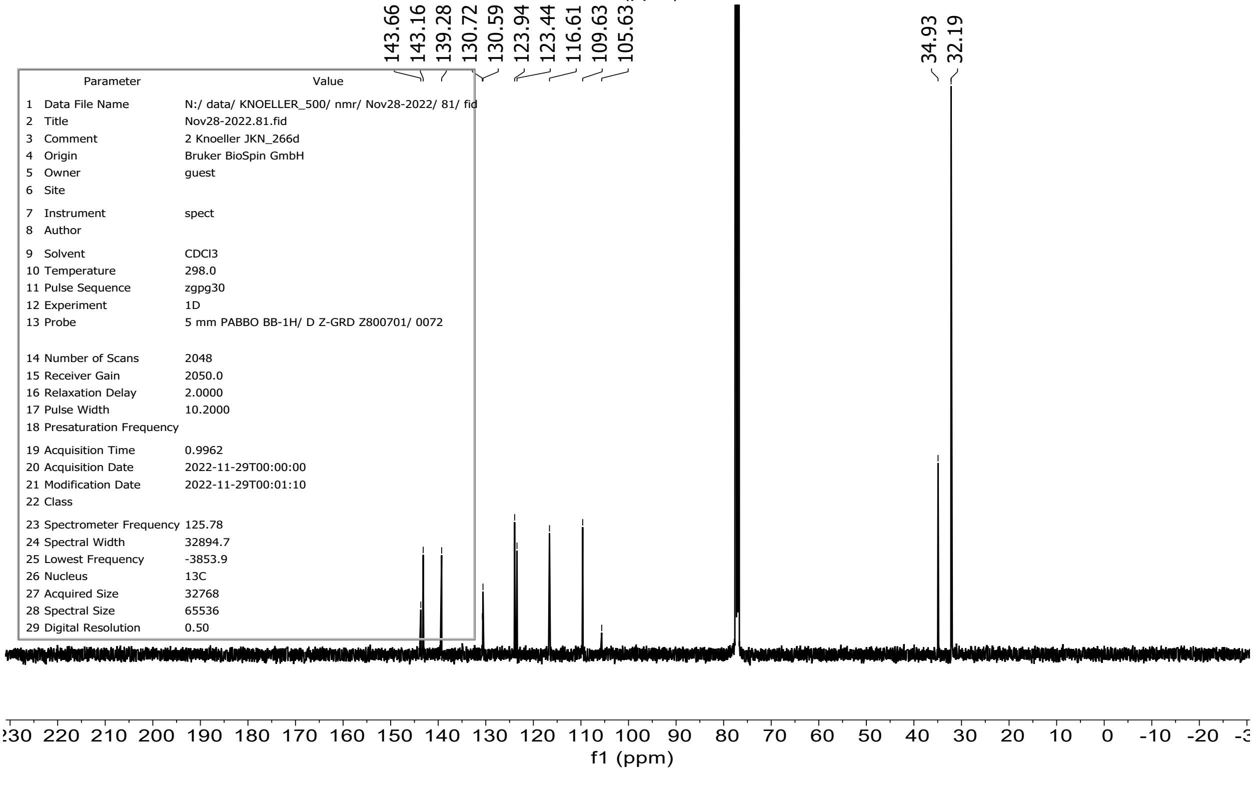


Figure **S3:** $^{1}H$ NMR spectrum (top, 500 MHz, inset shows aromatic region in detail) and $^{13}C$ NMR spectrum (bottom, 126 MHz) of **26** in $CDCl_3$.

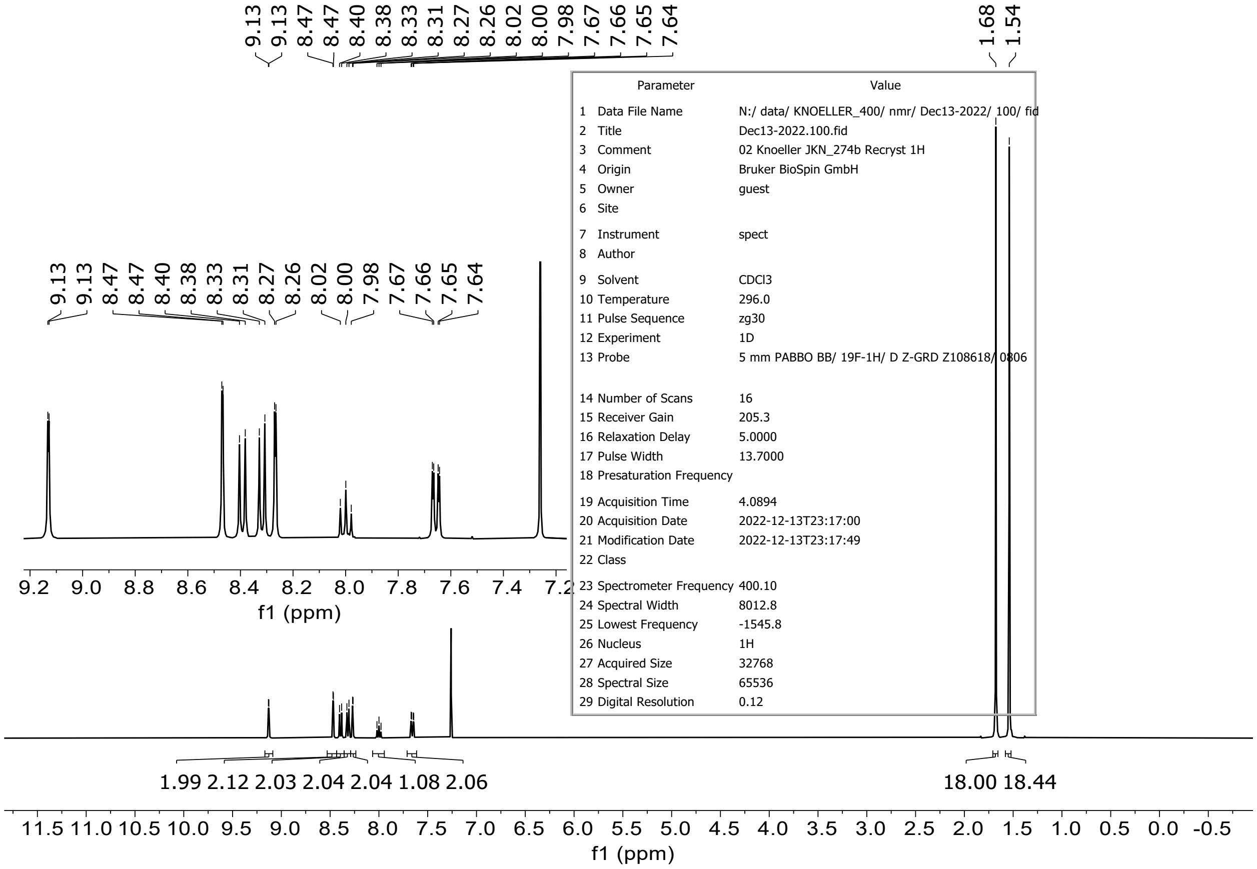

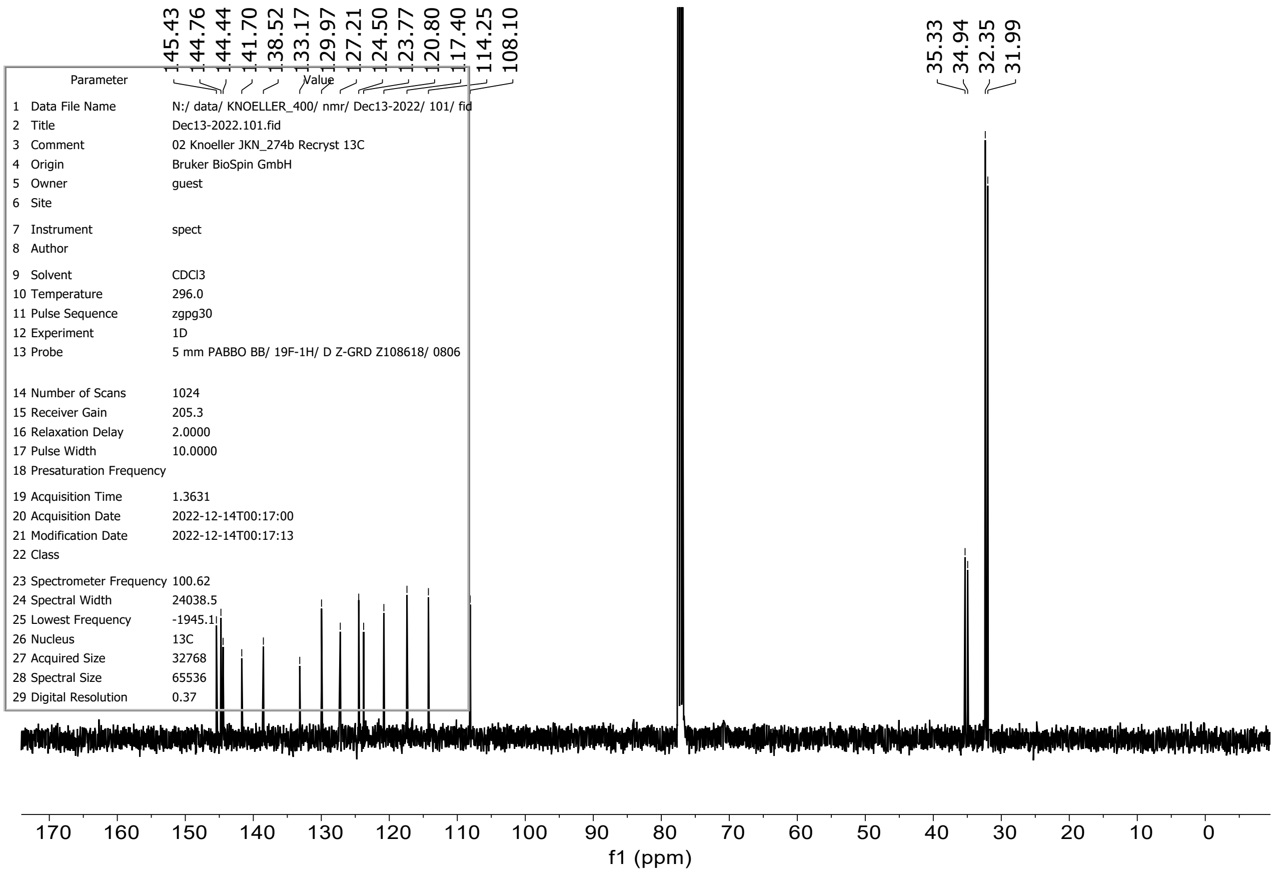

Figure **S4:** $^{1}$H NMR spectrum (top, 500 MHz, inset shows aromatic region in detail) and $^{13}$C NMR spectrum (bottom, 126 MHz) of **BCzBN** in $CDCl_3$.

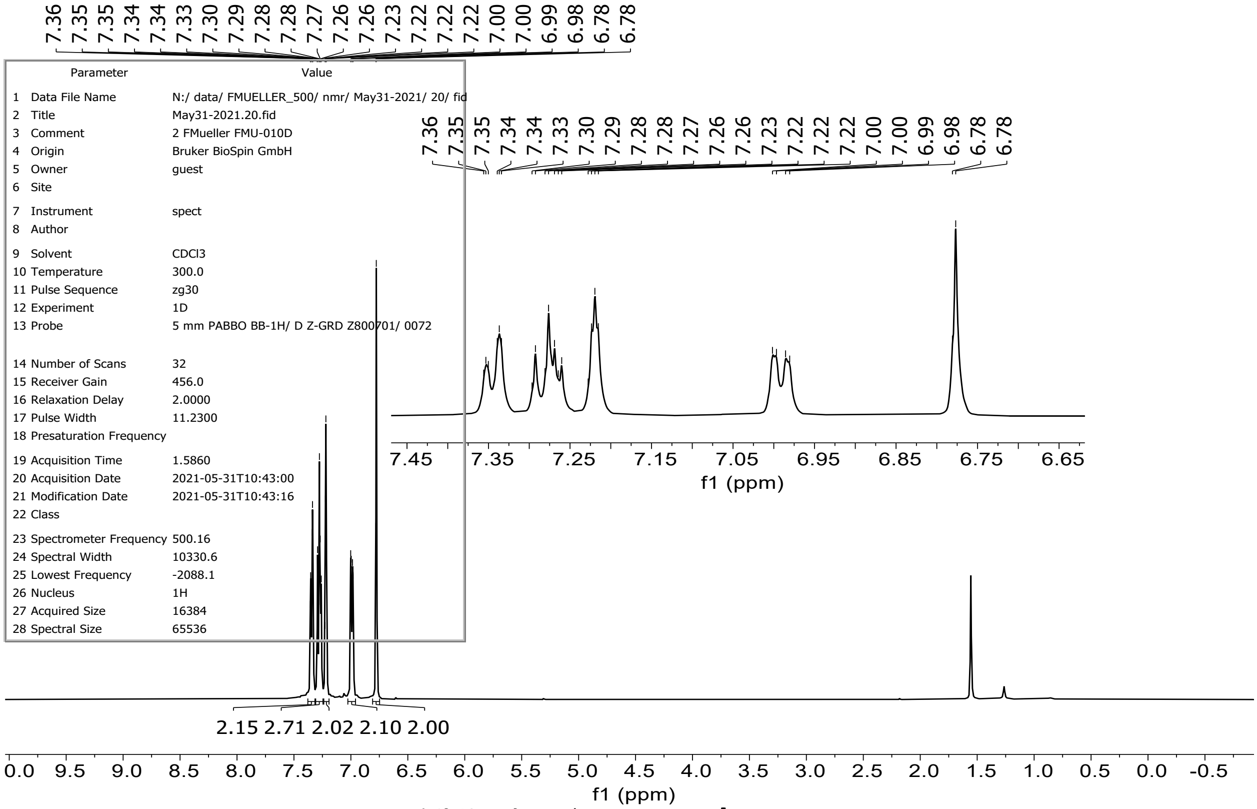


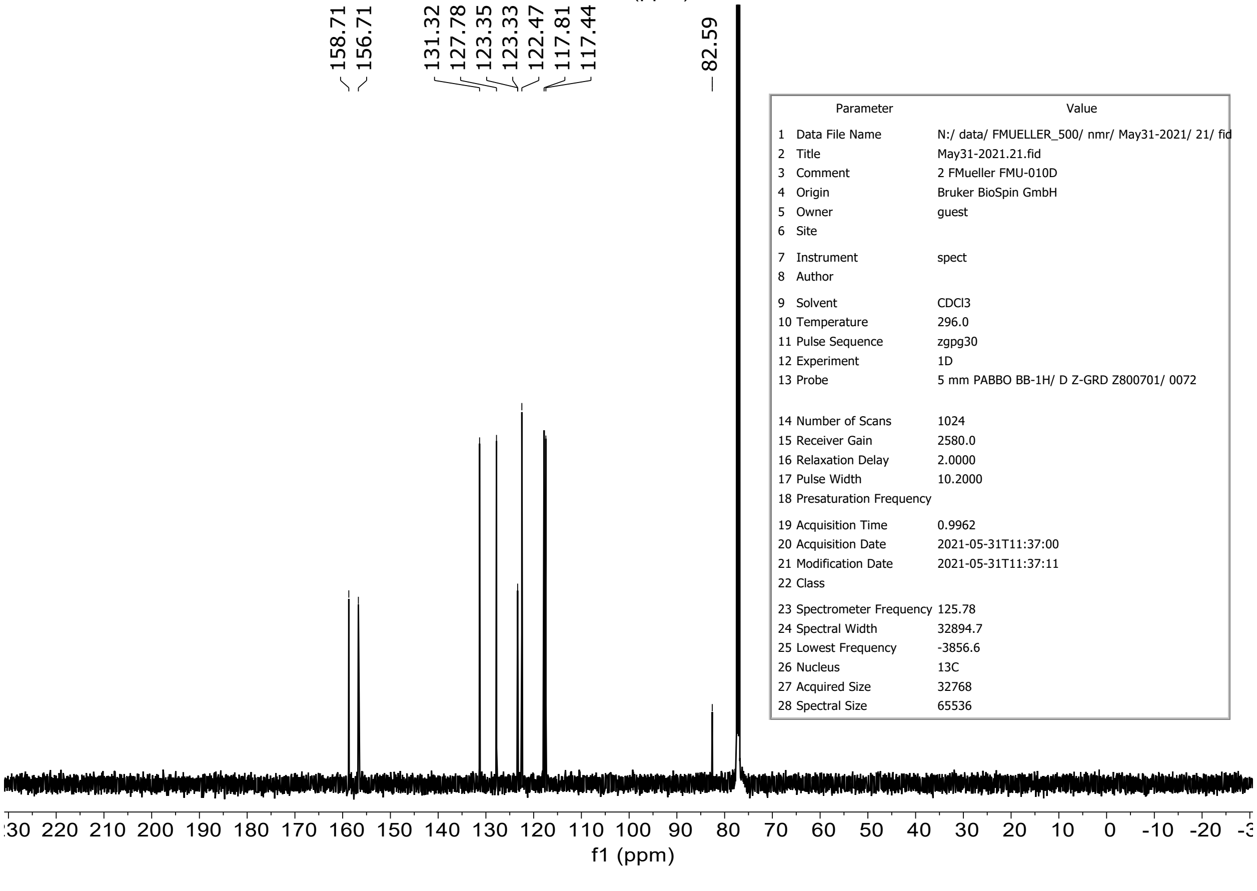


Figure **S5:** $^{1}$H NMR spectrum (top, 500 MHz, inset shows aromatic region in detail) and $^{13}$C NMR spectrum (bottom, 126 MHz) of **18** in $CDCl_3$.

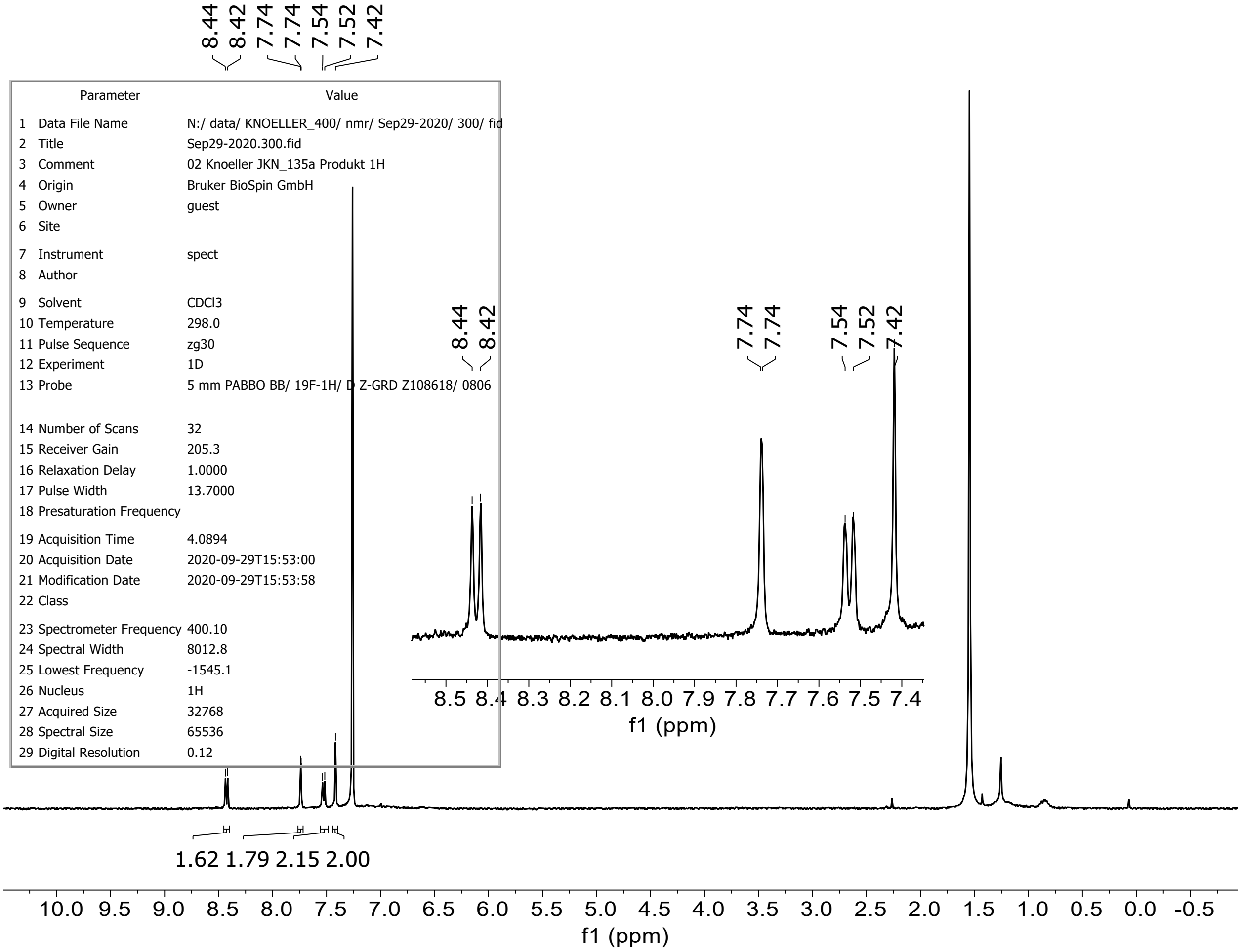


Figure **S6:** $^{1}$H NMR spectrum (700 MHz, inset shows aromatic region in detail) of **8** in $CDCl_3$.

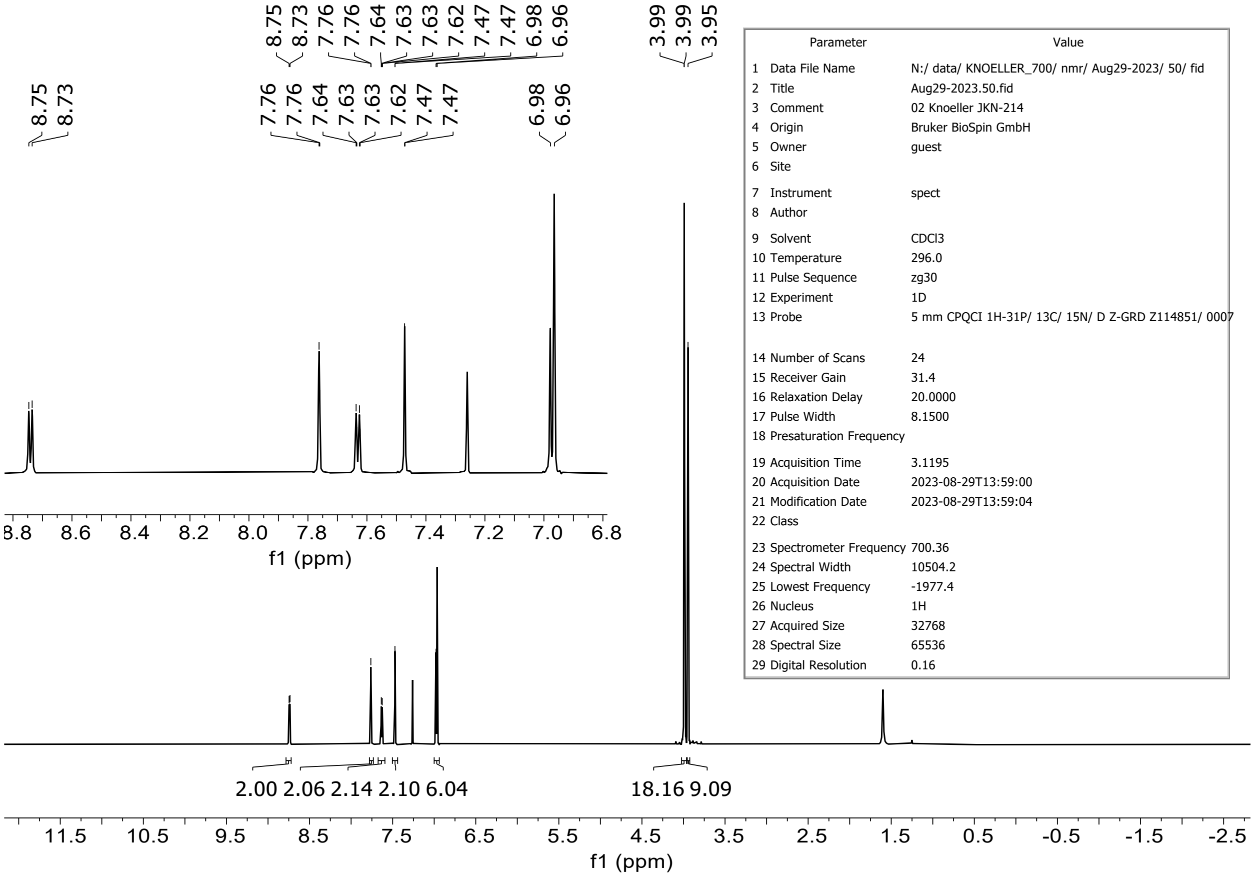


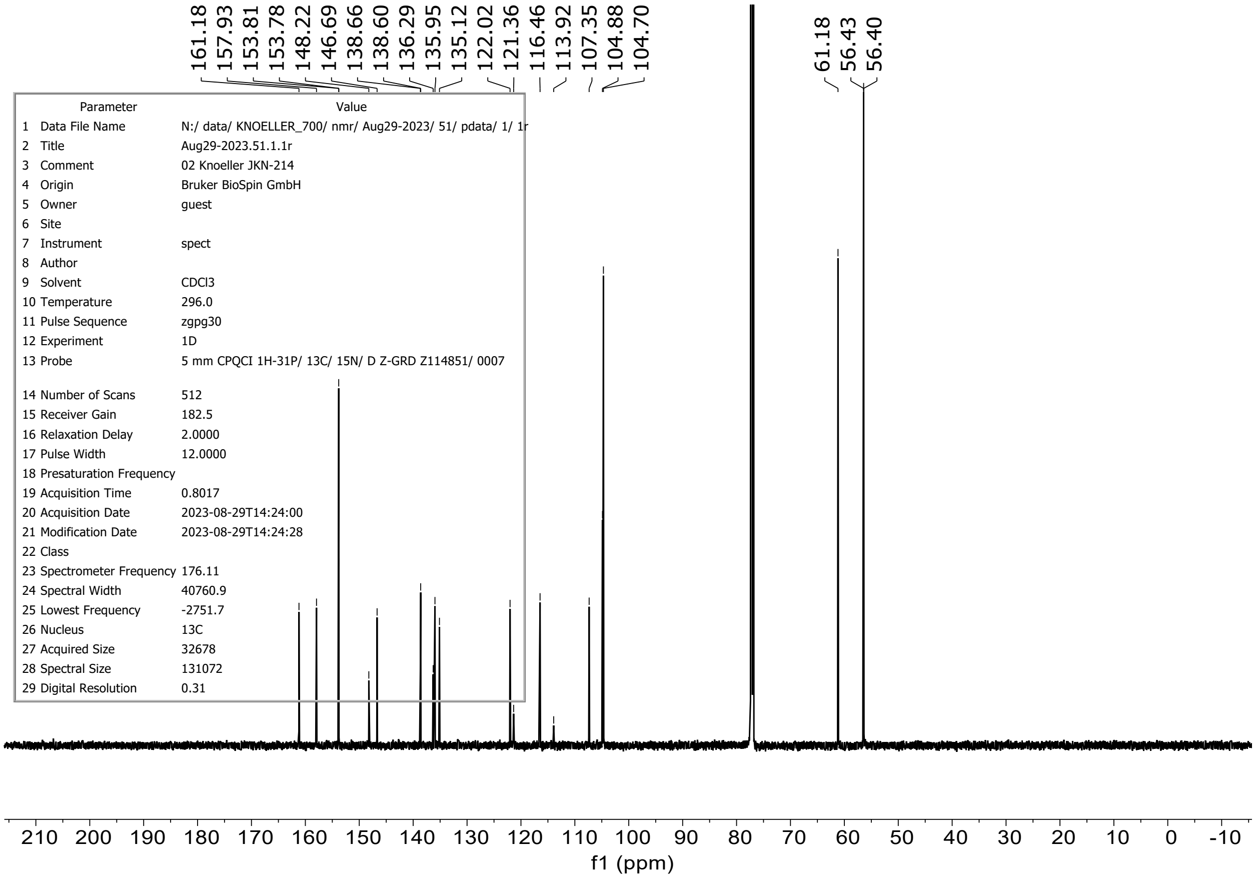


Figure **S7:** $^{1}H$ NMR spectrum (top, 700 MHz, inset shows aromatic region in detail) and $^{13}C$ NMR spectrum (bottom, 176 MHz) of **LC1** in $CDCl_3$.

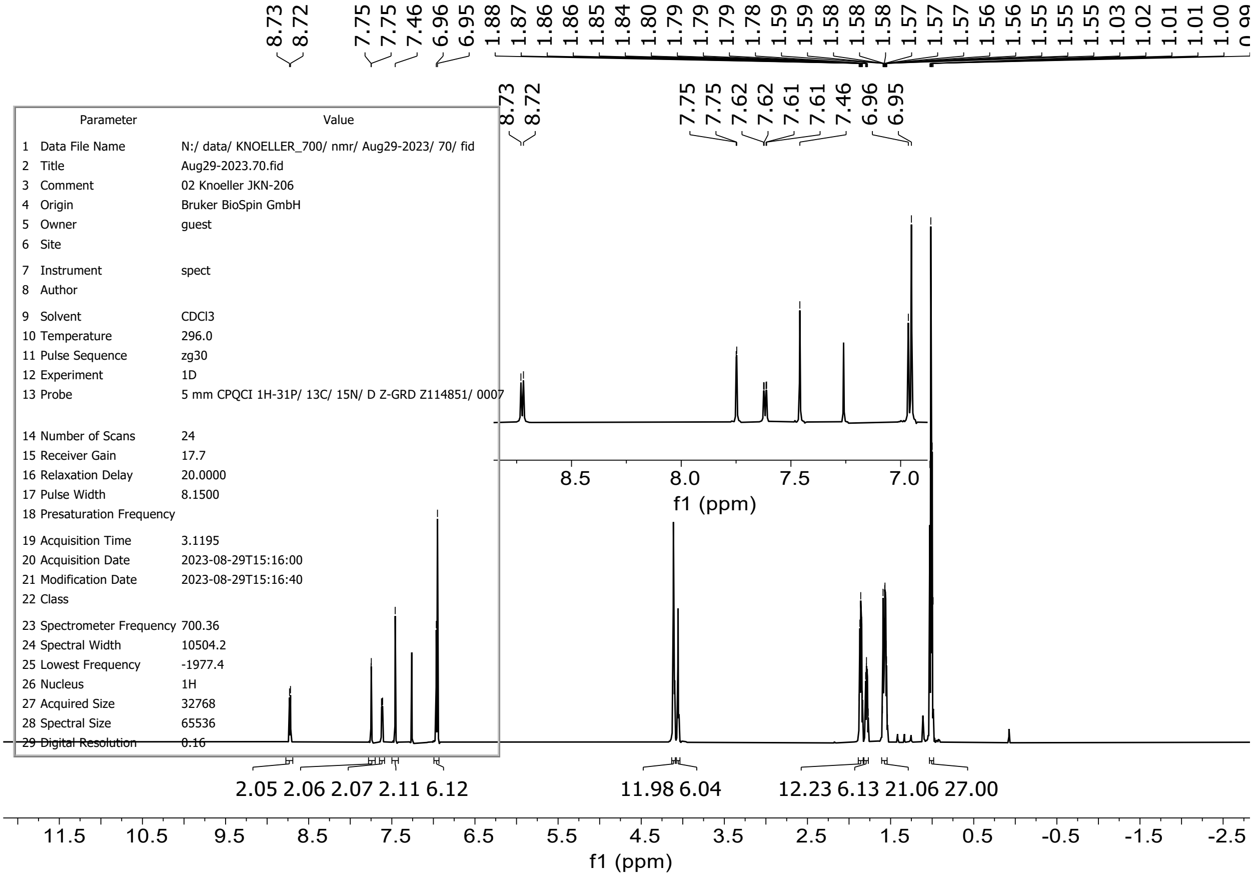


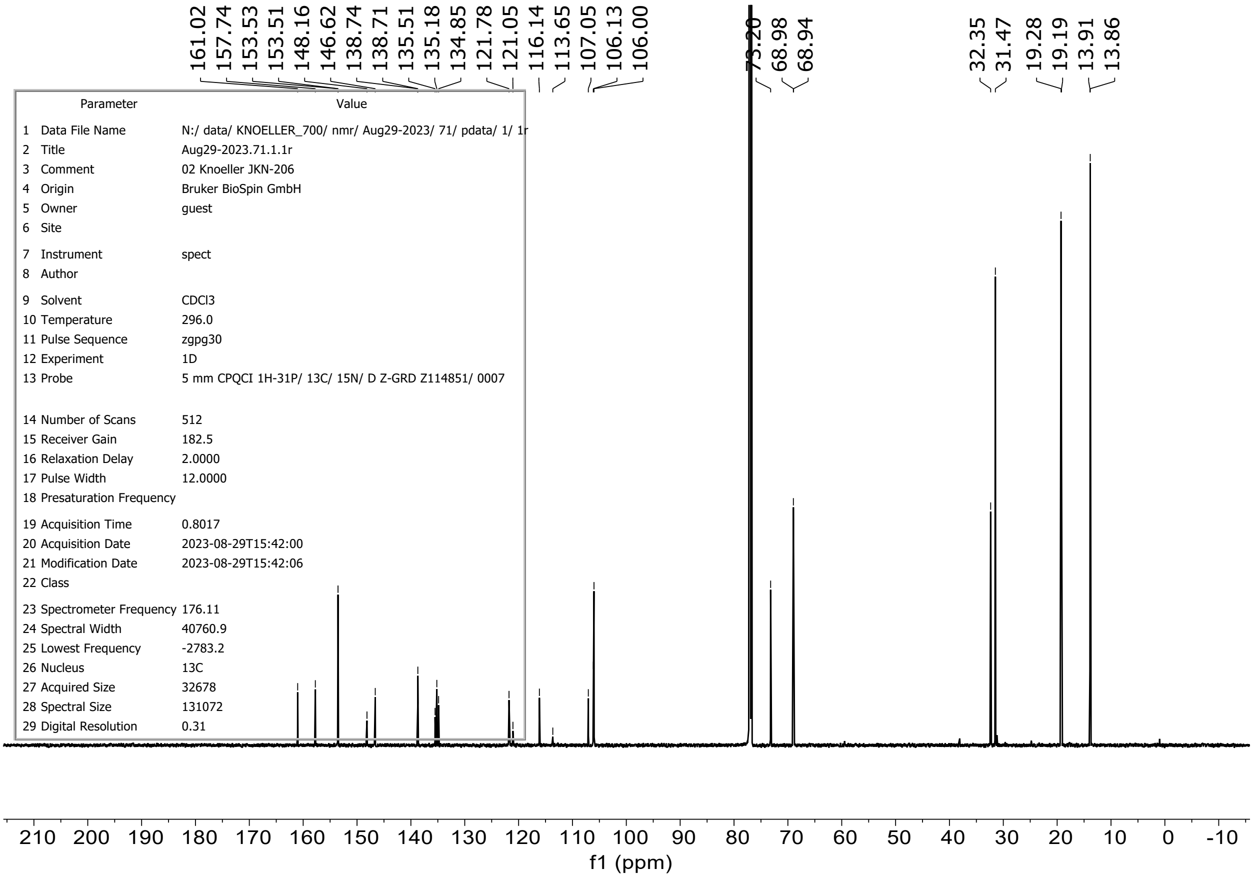


Figure **S8:** $^{1}$H NMR spectrum (top, 700 MHz, inset shows aromatic region in detail) and $^{13}$C NMR spectrum (bottom, 176 MHz) of **LC4** in $CDCl_3$.

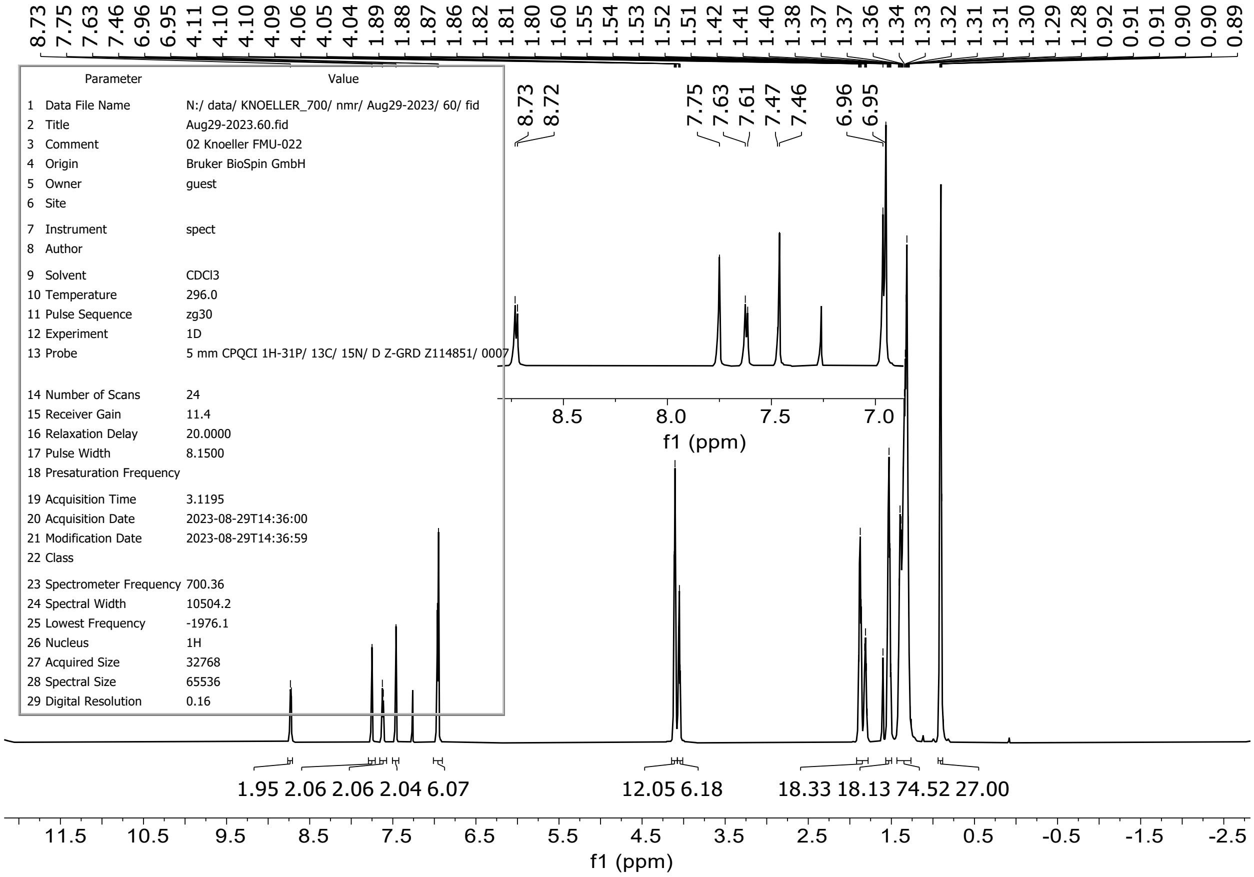


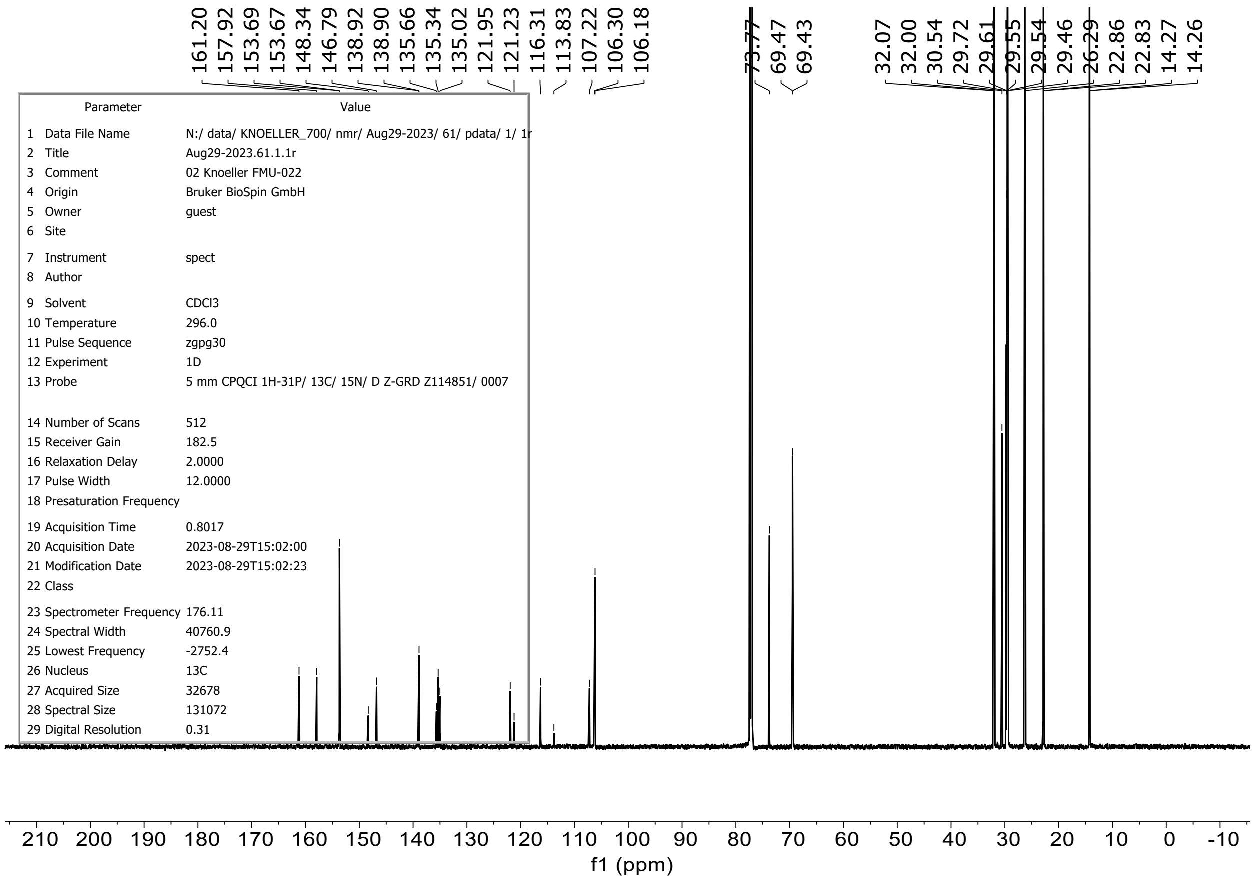


Figure **S9:** $^{1}H$ NMR spectrum (top, 700 MHz, inset shows aromatic region in detail) and $^{13}C$ NMR spectrum (bottom, 176 MHz) of **LC8** in $CDCl_3$.

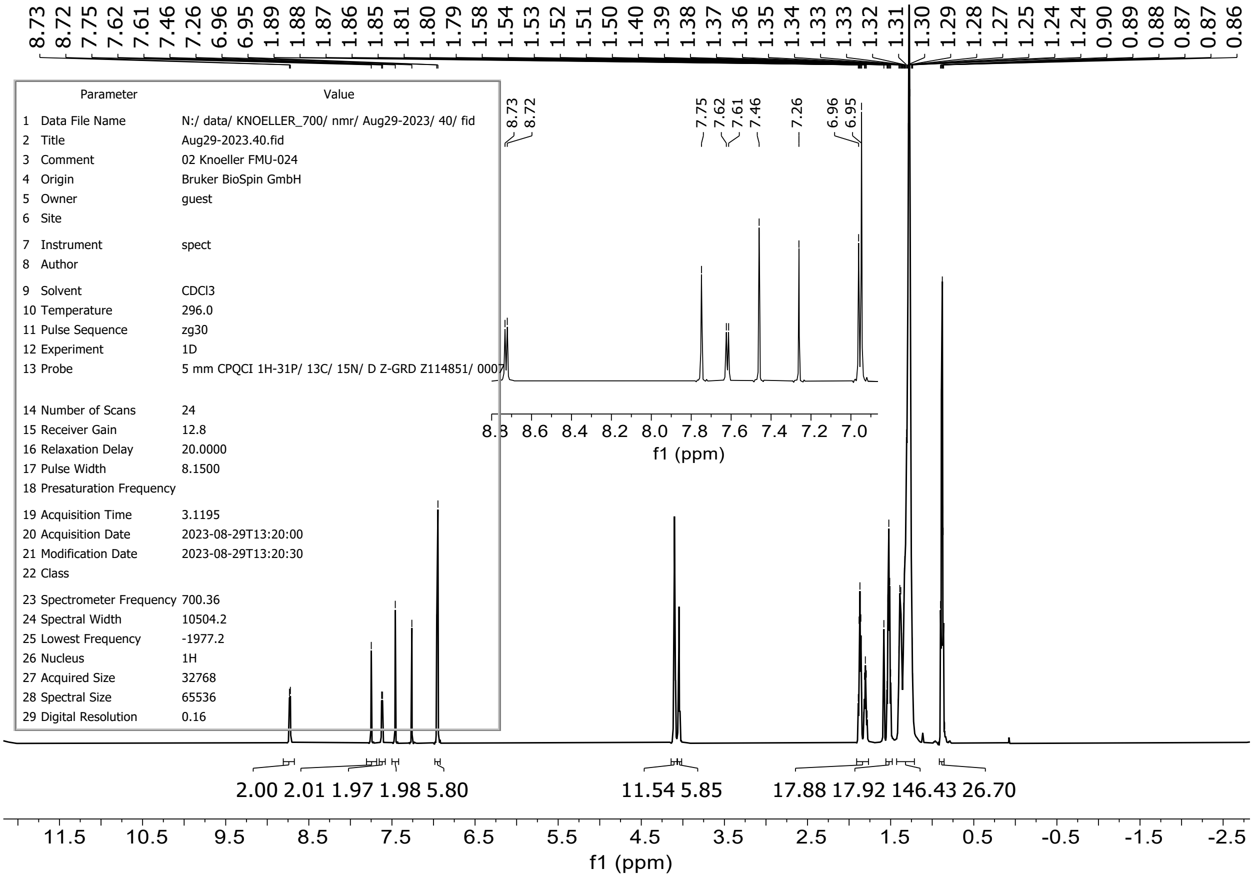


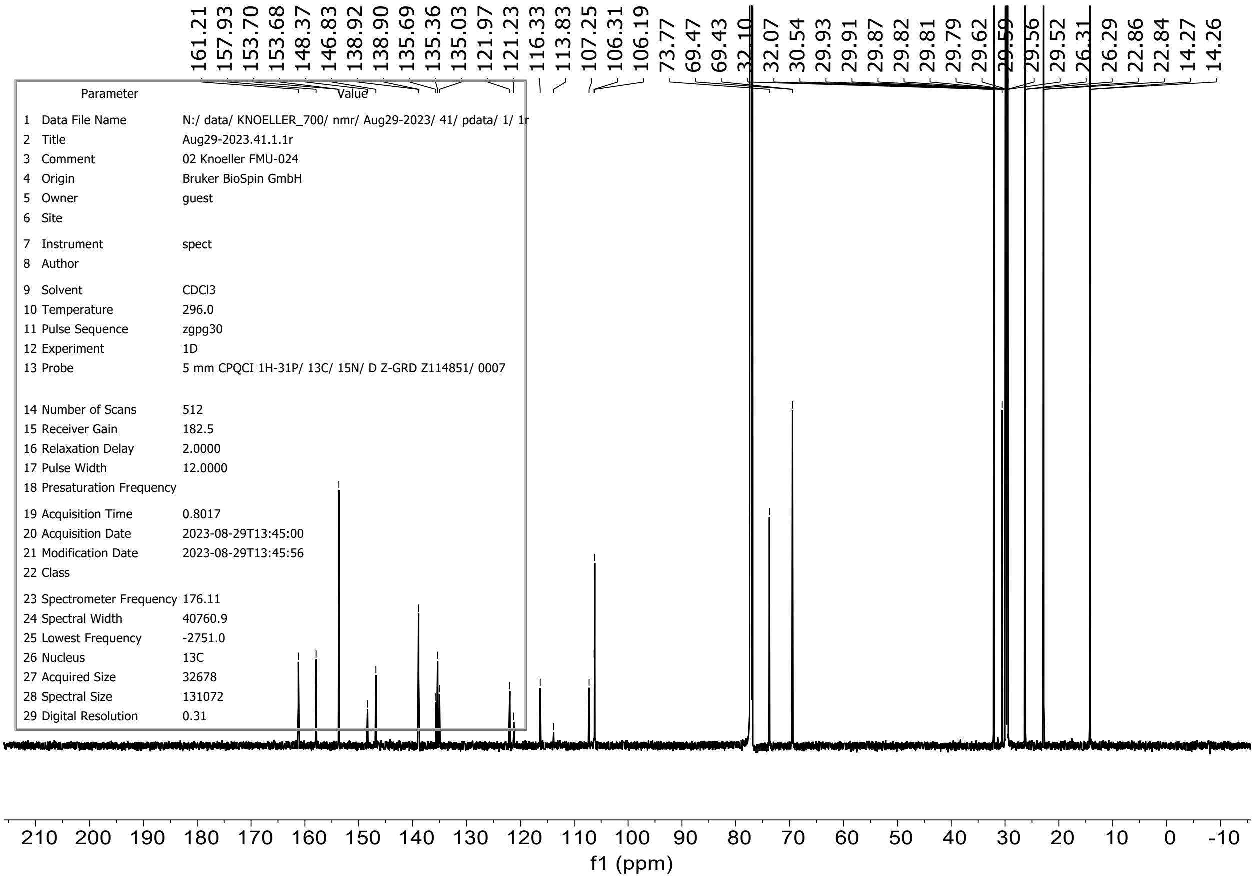


Figure **S10:** : $^{1}$H NMR spectrum (top, 700 MHz, inset shows aromatic region in detail) and $^{13}$C NMR spectrum (bottom, 176 MHz) of **LC12** in $CDCl_3$.

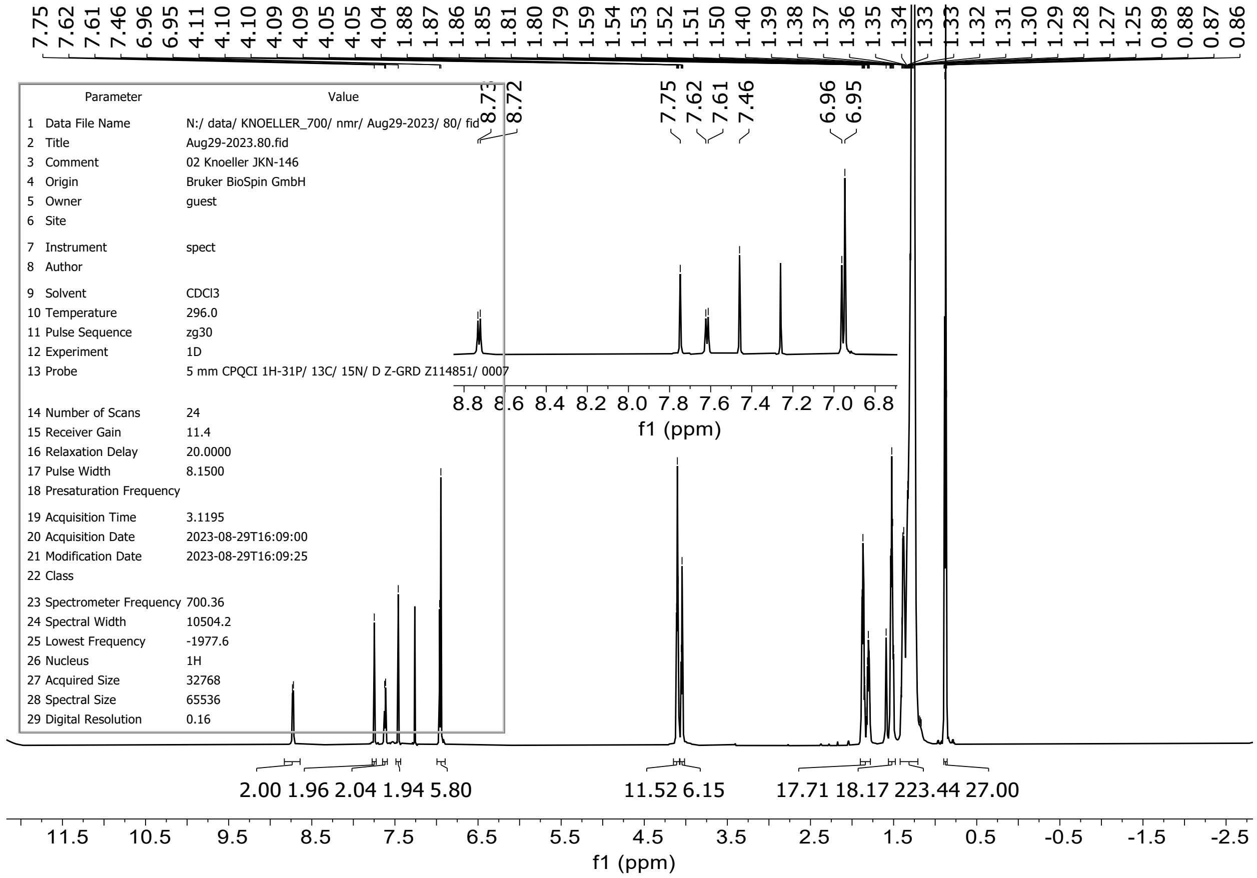

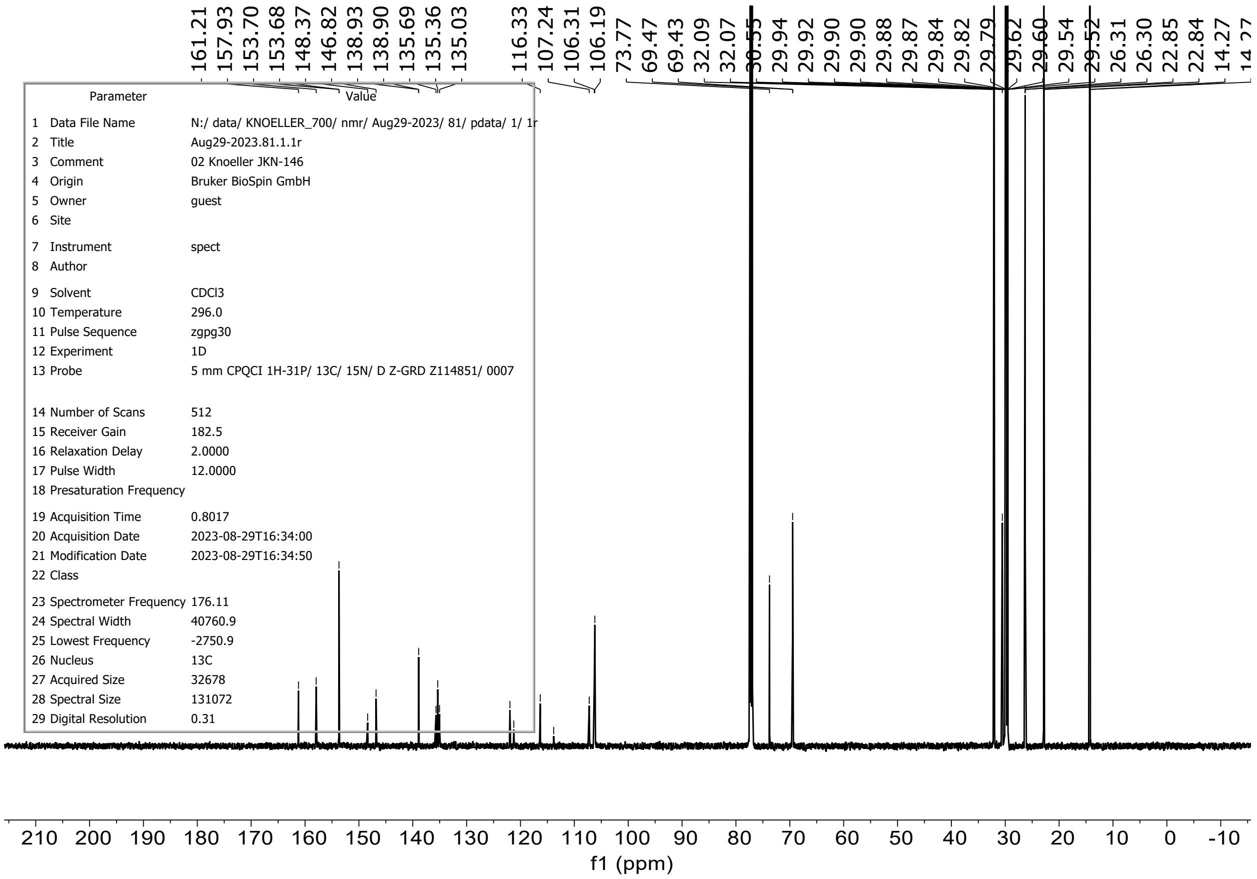

Figure **S11:** $^{1}$H NMR spectrum (top, 700 MHz, inset shows aromatic region in detail) and $^{13}$C NMR spectrum (bottom, 176 MHz) of **LC16** in $CDCl_3$.

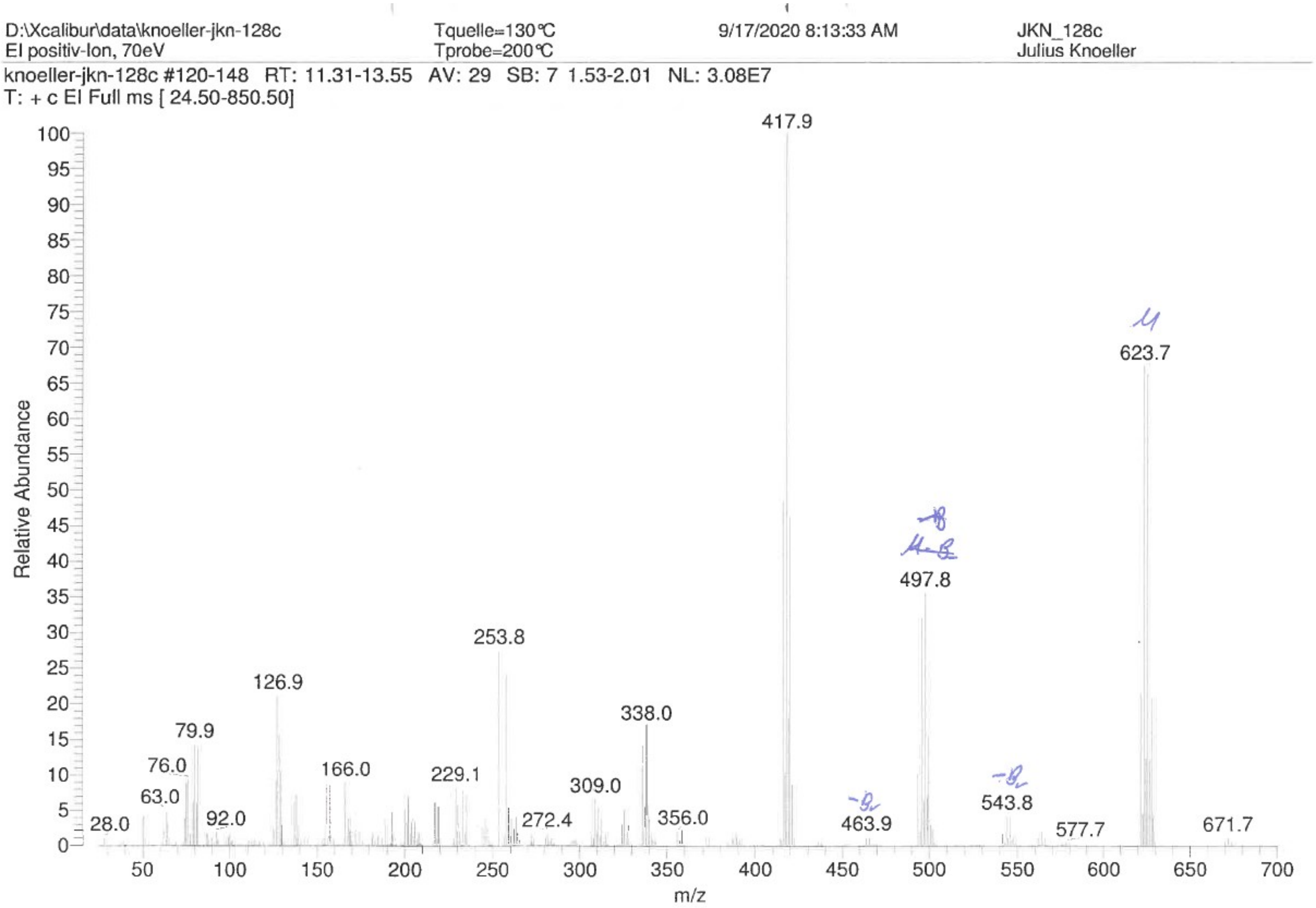


Figure **S12:** LRMS trace of **18**.

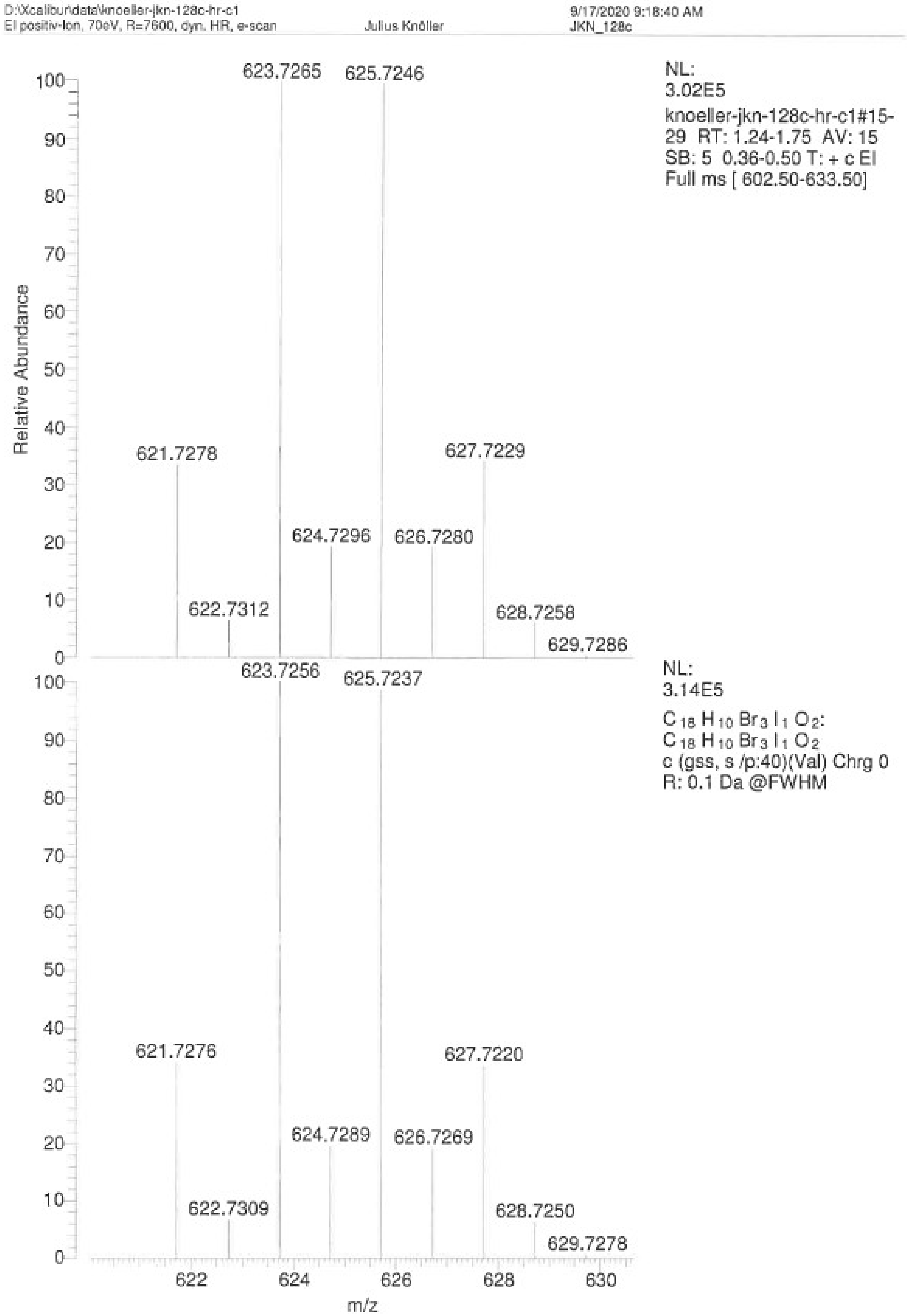


Figure **S13:** HRMS trace of **18**.

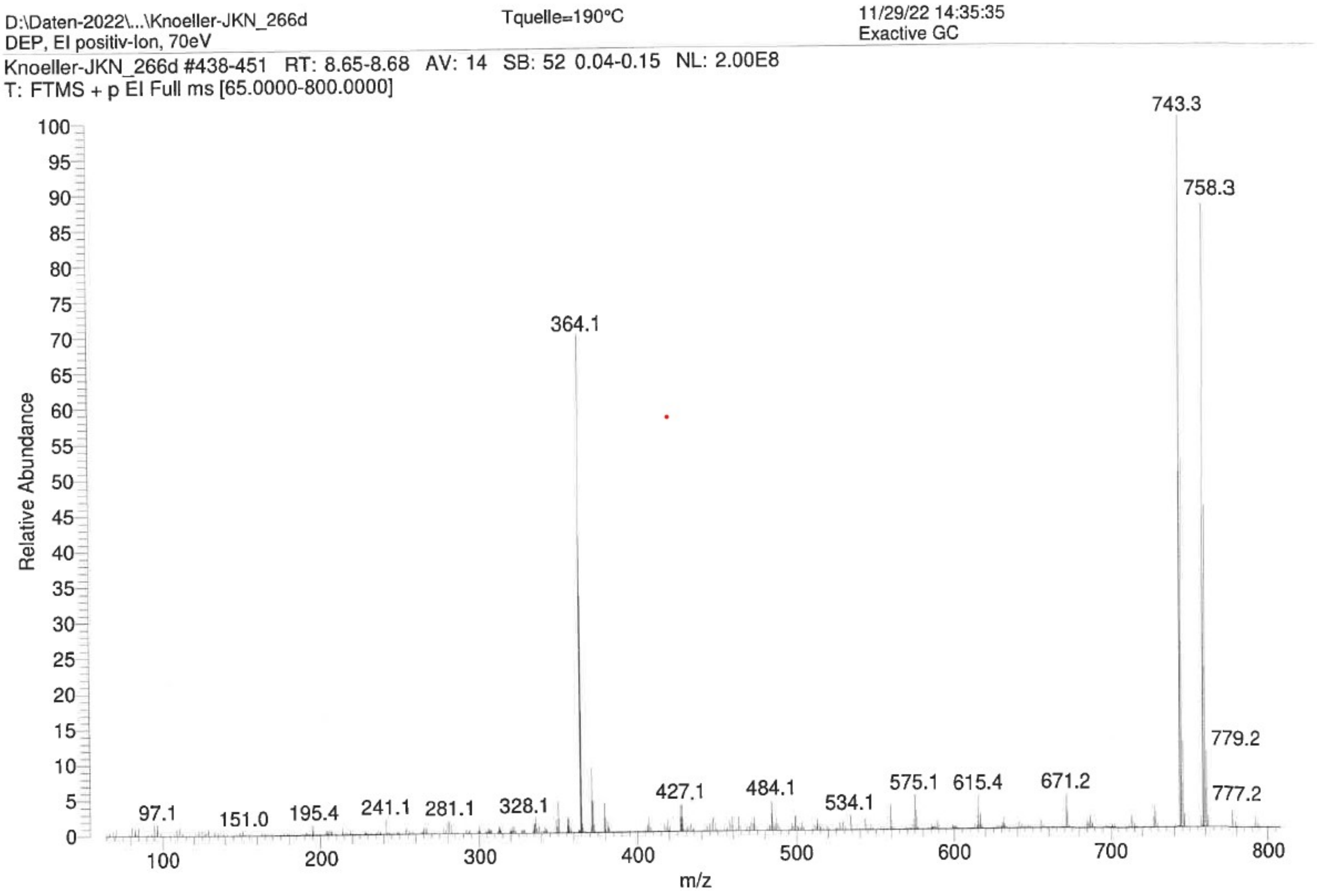


Figure **S14:** LRMS trace of **26**.

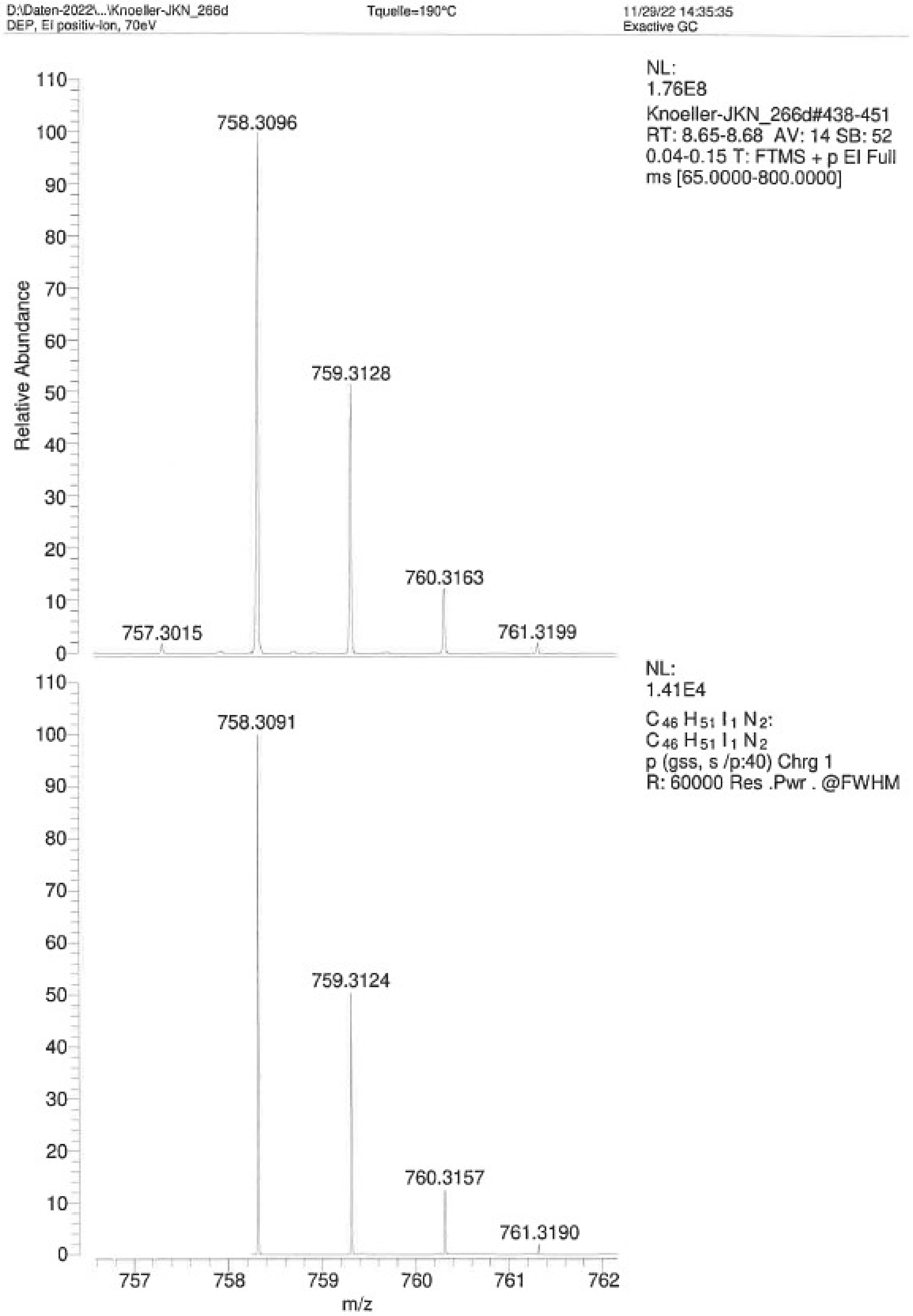


Figure **S15:** HRMS trace of **26**.

Knöller

# Massenspektrometrie - Universität Stuttgart

**Analysis Info**

| | | | |
|---|---|---|---|
| Analysis Name | Knöller-JKN_214b_3_01_35252.d | Acquisition Date | 10/14/2021 4:08:58 PM |
| Method | tune-wide-oktober-2019.m | | |
| Sample Name | Knöller-JKN_214b | Operator | BDAL@DE |
| Comment | | Instrument | micrOTOF-Q 228888.00043 |

**Acquisition Parameter**

| | | | | | |
|---|---|---|---|---|---|
| Source Type | ESI | Ion Polarity | Positive | Set Nebulizer | 0.4 Bar |
| Focus | Not active | Set Capillary | 4500 V | Set Dry Heater | 200 °C |
| Scan Begin | 250 m/z | Set End Plate Offset | -500 V | Set Dry Gas | 4.0 l/min |
| Scan End | 3000 m/z | Set Collision Cell RF | 500.0 Vpp | Set Divert Valve | Waste |

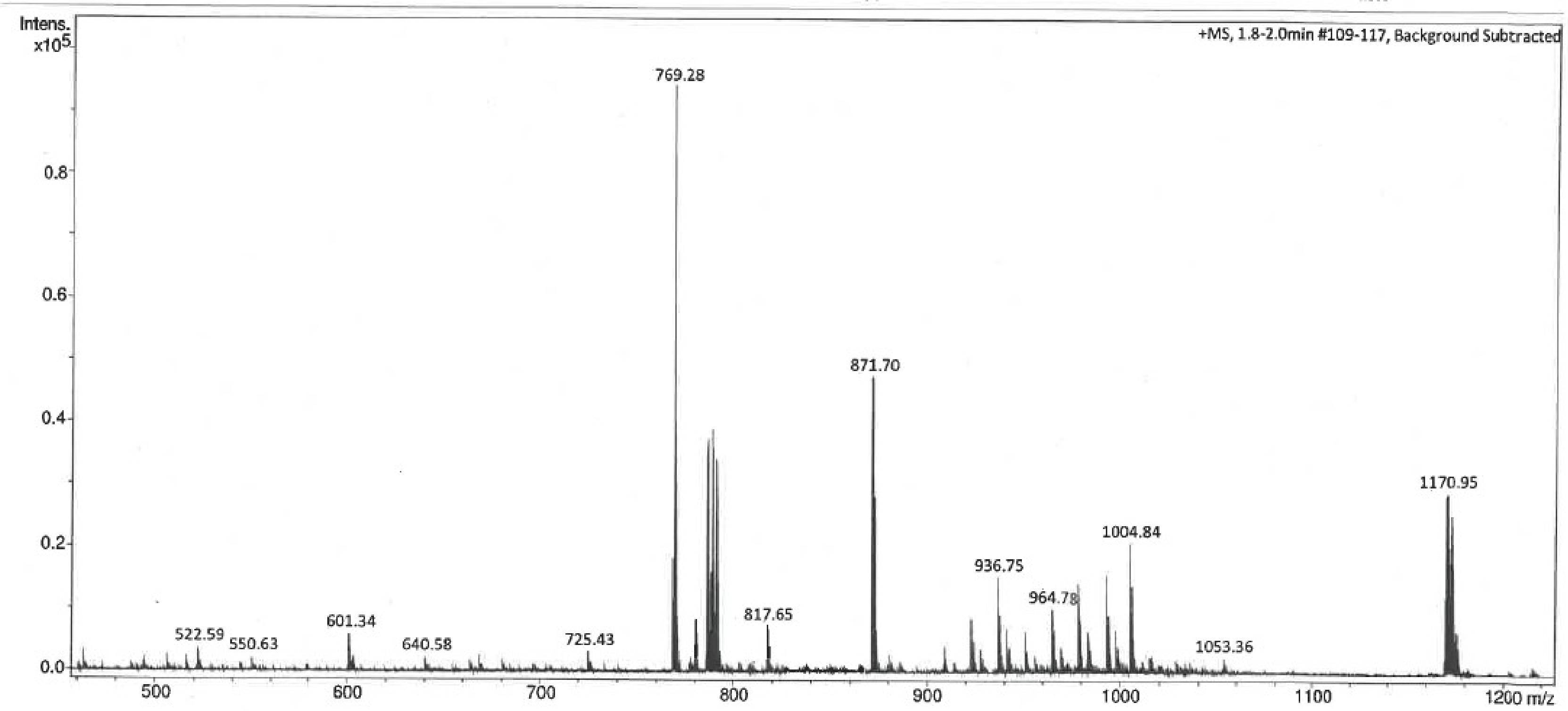


Bruker Compass DataAnalysis 4.2 printed: 10/14/2021 4:20:39 PM Page 1 of 1

Figure **S16:** LRMS trace of **LC1**.

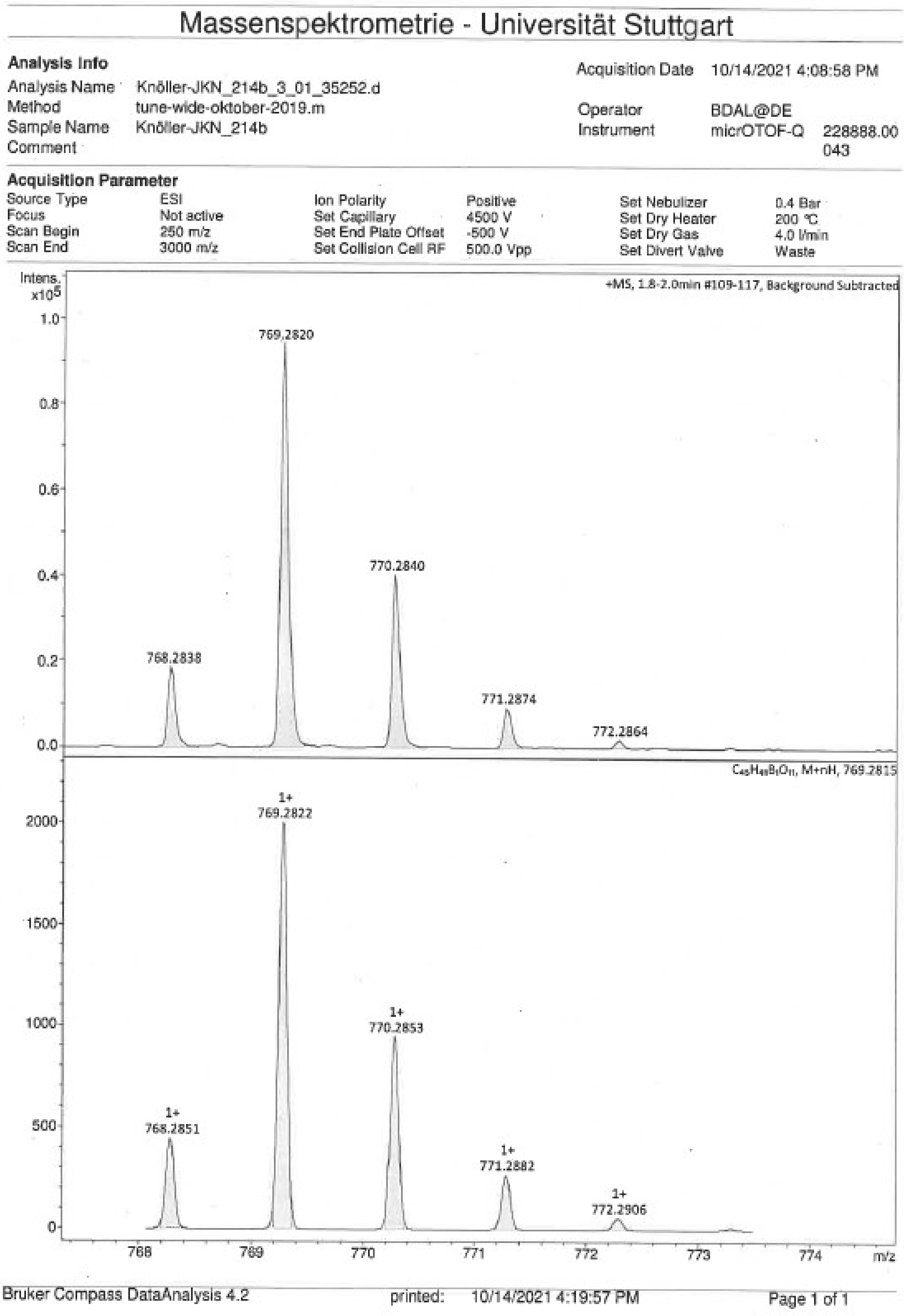


Figure **S17:** HRMS trace of **LC1**.

## Massenspektrometrie - Universität Stuttgart

**Analysis Info**

| | | | |
|---|---|---|---|
| Analysis Name | Knoeller-JKN_206b_APCI.d | Acquisition Date | 8/12/2021 11:12:19 AM |
| Method | tune-low-oktober-2019.m | Operator | BDAL@DE |
| Sample Name | Knoeller-JKN_206b_APCI | Instrument | micrOTOF-Q 228888.00043 |
| Comment | | | |

**Acquisition Parameter**

| | | | | | |
|---|---|---|---|---|---|
| Source Type | APCI | Ion Polarity | Positive | Set Nebulizer | 1.7 Bar |
| Focus | Not active | Set Capillary | 4500 V | Set Dry Heater | 300 °C |
| Scan Begin | 50 m/z | Set End Plate Offset | -500 V | Set Dry Gas | 5.0 l/min |
| Scan End | 1500 m/z | Set Collision Cell RF | 180.0 Vpp | Set Divert Valve | Waste |

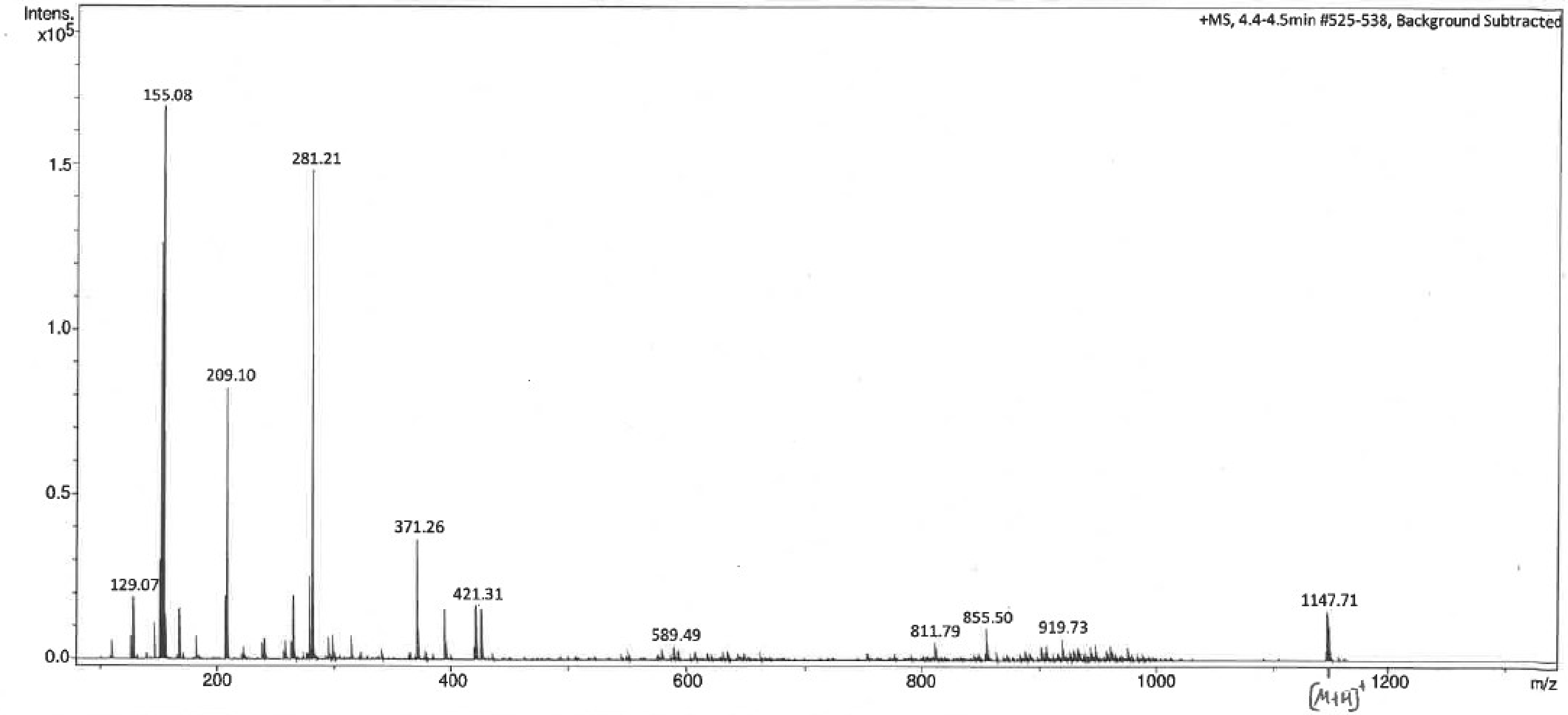


Bruker Compass DataAnalysis 4.2 printed: 8/12/2021 11:27:06 AM Page 1 of 1

Figure **S18:** LRMS trace of **LC4**.

## Massenspektrometrie - Universität Stuttgart

**Analysis Info**

| | | | |
|---|---|---|---|
| Analysis Name | Knoeller-JKN_206b_APCI.d | Acquisition Date | 8/12/2021 11:12:19 AM |
| Method | tune-low-oktober-2019.m | | |
| Sample Name | Knoeller-JKN_206b_APCI | Operator | BDAL@DE |
| Comment | | Instrument | micrOTOF-Q 228888.00043 |

**Acquisition Parameter**

| | | | | | |
|---|---|---|---|---|---|
| Source Type | APCI | Ion Polarity | Positive | Set Nebulizer | 1.7 Bar |
| Focus | Not active | Set Capillary | 4500 V | Set Dry Heater | 300 °C |
| Scan Begin | 50 m/z | Set End Plate Offset | -500 V | Set Dry Gas | 5.0 l/min |
| Scan End | 1500 m/z | Set Collision Cell RF | 180.0 Vpp | Set Divert Valve | Waste |

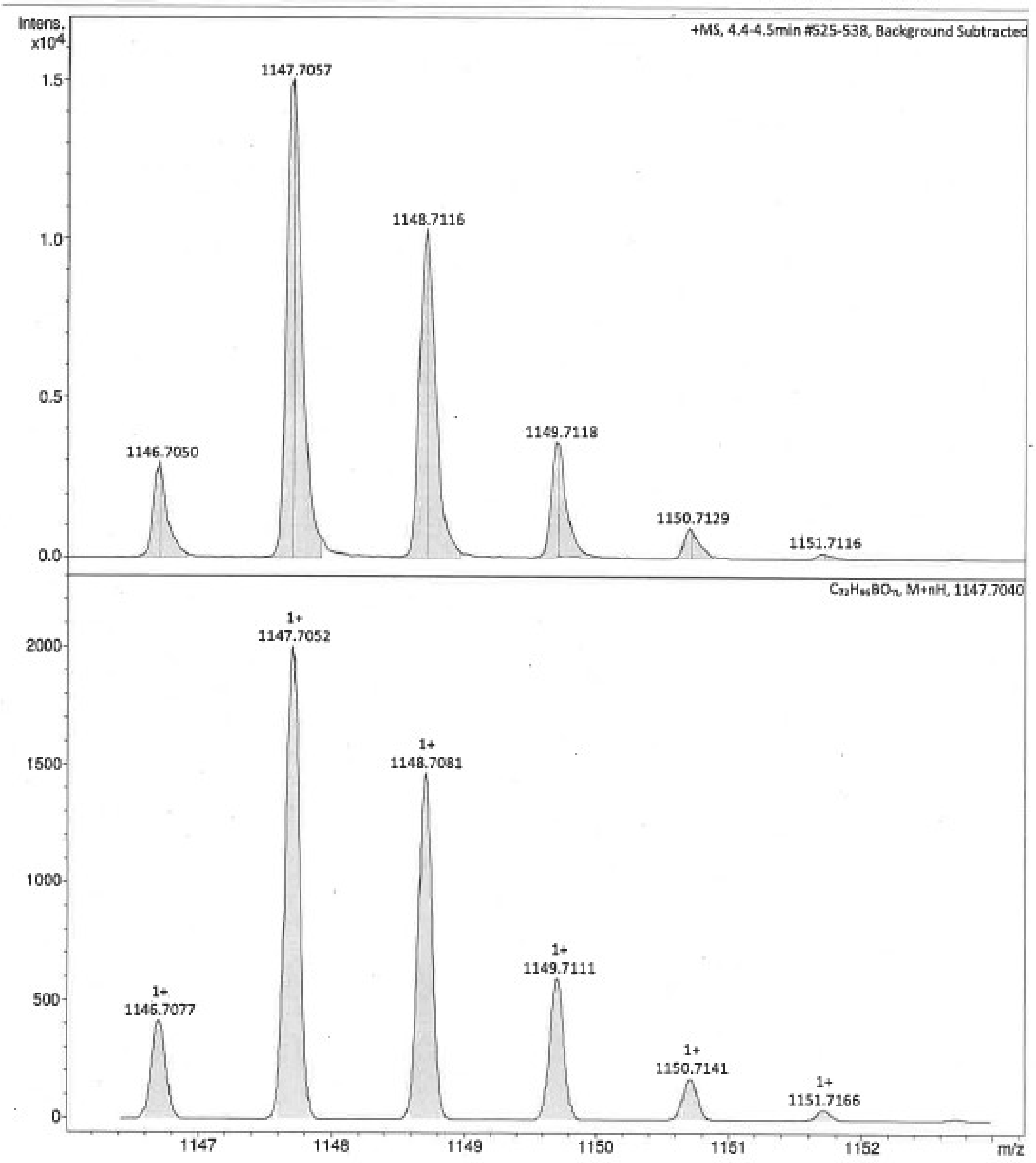


Bruker Compass DataAnalysis 4.2 printed: 8/12/2021 11:26:26 AM Page 1 of 1

Figure **S19:** HRMS trace of **LC4**.

## Massenspektrometrie - Universität Stuttgart

**Analysis Info**

| | | | |
|---|---|---|---|
| Analysis Name | Müller-FMU_022B_12_01_33005.d | Acquisition Date | 7/6/2021 9:33:07 AM |
| Method | tune-wide-oktober-2019.m | Operator | BDAL@DE |
| Sample Name | Müller-FMU_022B | Instrument | micrOTOF-Q 228888.00043 |
| Comment | | | |

**Acquisition Parameter**

| | | | | | | | |
|---|---|---|---|---|---|---|---|
| Source Type | APCI | Ion Polarity | Positive | Set Nebulizer | 1.7 Bar | | |
| Focus | Not active | Set Capillary | 4500 V | Set Dry Heater | 300 °C | | |
| Scan Begin | 250 m/z | Set End Plate Offset | -500 V | Set Dry Gas | 5.0 l/min | | |
| Scan End | 3000 m/z | Set Collision Cell RF | 500.0 Vpp | Set Divert Valve | Waste | | |

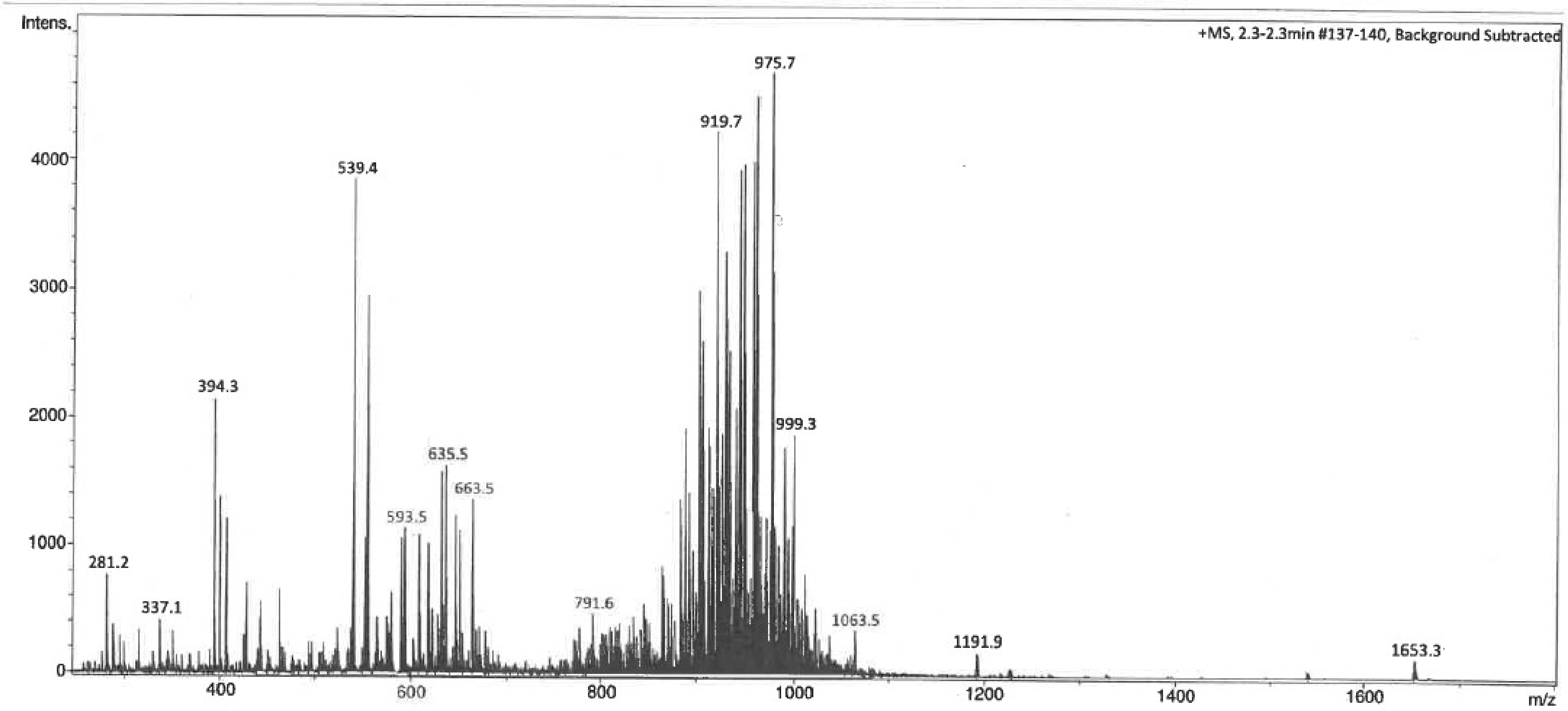


Bruker Compass DataAnalysis 4.2 printed: 7/6/2021 10:13:13 AM Page 1 of 1

Figure **S20:** LRMS trace of **LC8**.

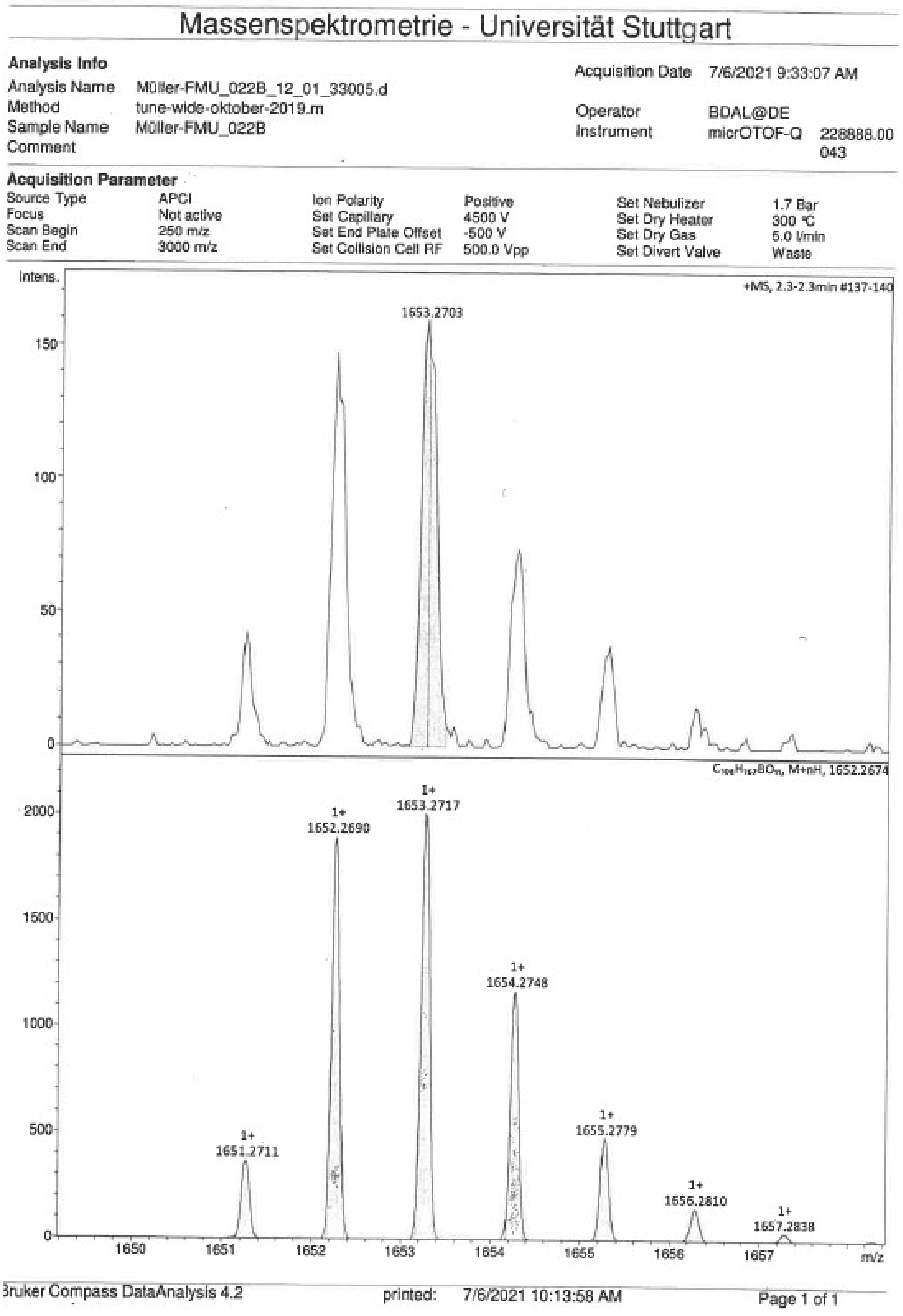


Figure **S21:** HRMS trace of **LC8**.

Müller

## Massenspektrometrie - Universität Stuttgart

**Analysis Info**

| | | | |
|---|---|---|---|
| Analysis Name | Mueller-FMU_024.d | Acquisition Date | 7/20/2021 3:23:33 PM |
| Method | tune-high-oktober-2019.m | Operator | BDAL@DE |
| Sample Name | Müller-FMU_024 | Instrument | micrOTOF-Q 228888.00043 |
| Comment | CHCl3:MeCN=1:1 | | |

**Acquisition Parameter**

| | | | | | |
|---|---|---|---|---|---|
| Source Type | APCI | Ion Polarity | Positive | Set Nebulizer | 1.7 Bar |
| Focus | Active | Set Capillary | 4500 V | Set Dry Heater | 300 °C |
| Scan Begin | 800 m/z | Set End Plate Offset | -500 V | Set Dry Gas | 5.0 l/min |
| Scan End | 5500 m/z | Set Collision Cell RF | 1600.0 Vpp | Set Divert Valve | Waste |

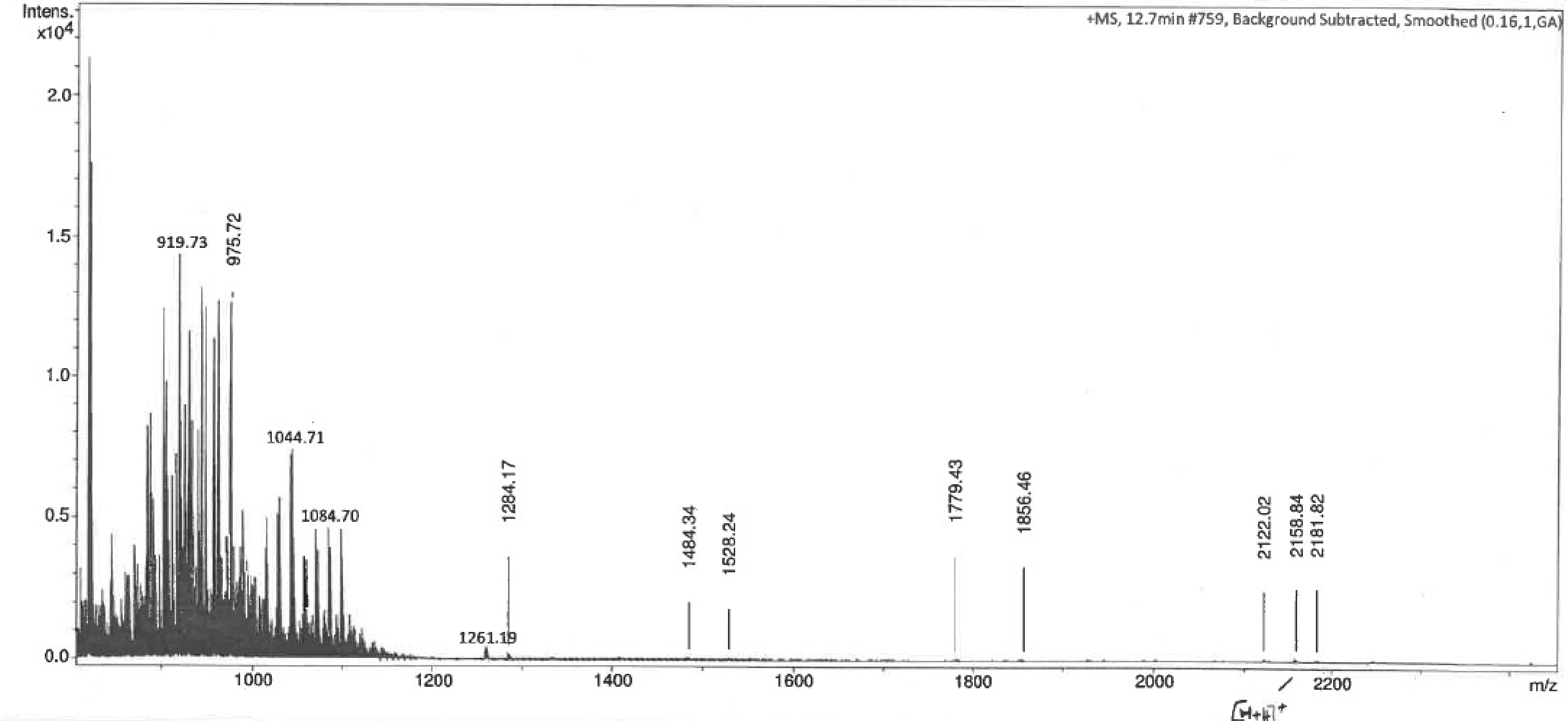


Bruker Compass DataAnalysis 4.2 printed: 7/20/2021 4:45:25 PM Page 1 of 1

Figure **S22:** LRMS trace of **LC12**.

## Massenspektrometrie - Universität Stuttgart

**Analysis Info**

| | | | |
|---|---|---|---|
| Analysis Name | Mueller-FMU_024.d | Acquisition Date | 7/20/2021 3:23:33 PM |
| Method | tune-high-oktober-2019.m | Operator | BDAL@DE |
| Sample Name | Müller-FMU_024 | Instrument | micrOTOF-Q 228888.00043 |
| Comment | CHCl3:MeCN=1:1 | | |

**Acquisition Parameter**

| | | | | | | | |
|---|---|---|---|---|---|---|---|
| Source Type | APCI | Ion Polarity | Positive | Set Nebulizer | 1.7 Bar | | |
| Focus | Active | Set Capillary | 4500 V | Set Dry Heater | 300 °C | | |
| Scan Begin | 800 m/z | Set End Plate Offset | -500 V | Set Dry Gas | 5.0 l/min | | |
| Scan End | 5500 m/z | Set Collision Cell RF | 1600.0 Vpp | Set Divert Valve | Waste | | |

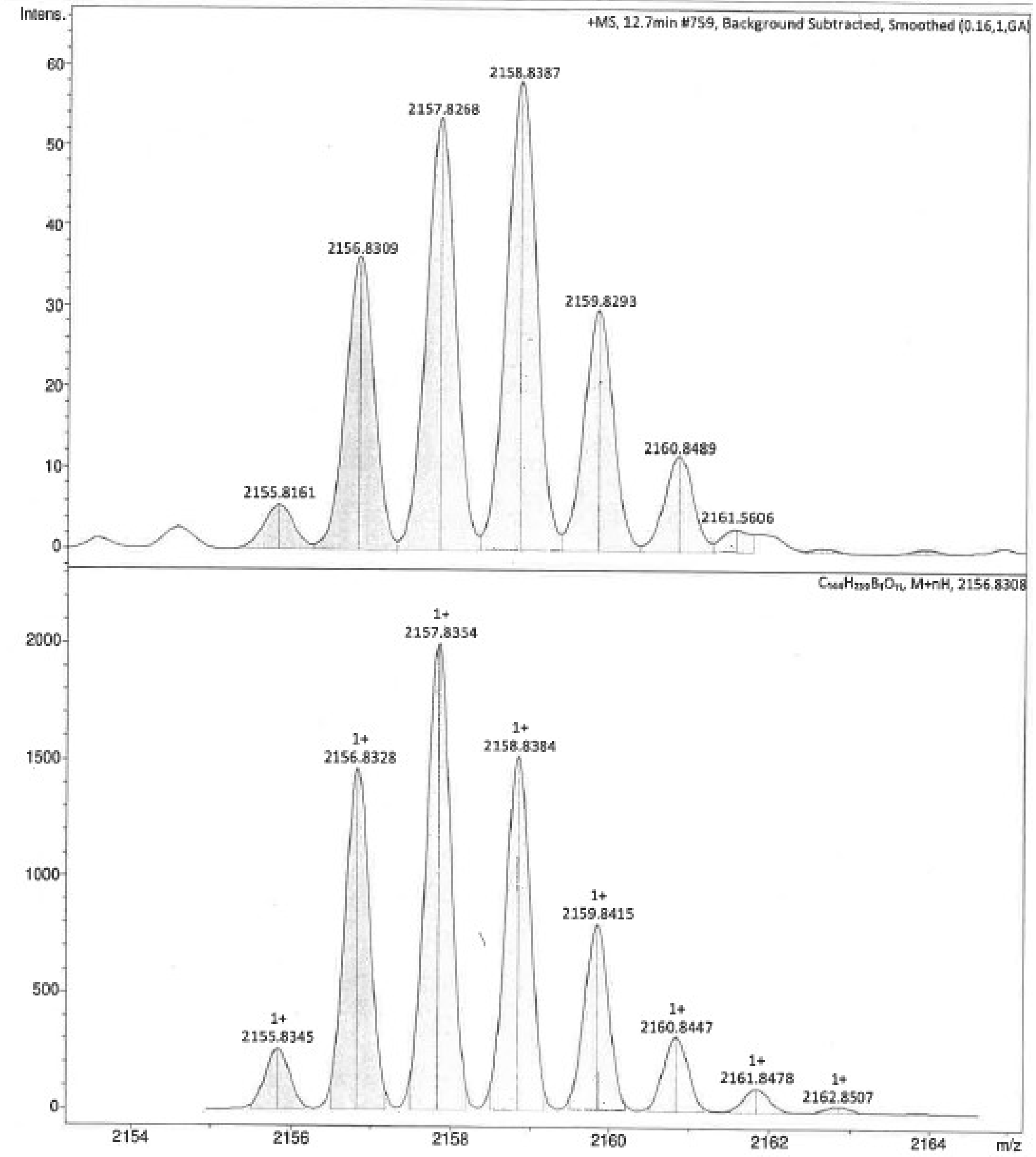


Bruker Compass DataAnalysis 4.2 printed: 7/20/2021 4:42:26 PM Page 1 of 1

Figure **S23:** HRMS trace of **LC12**.

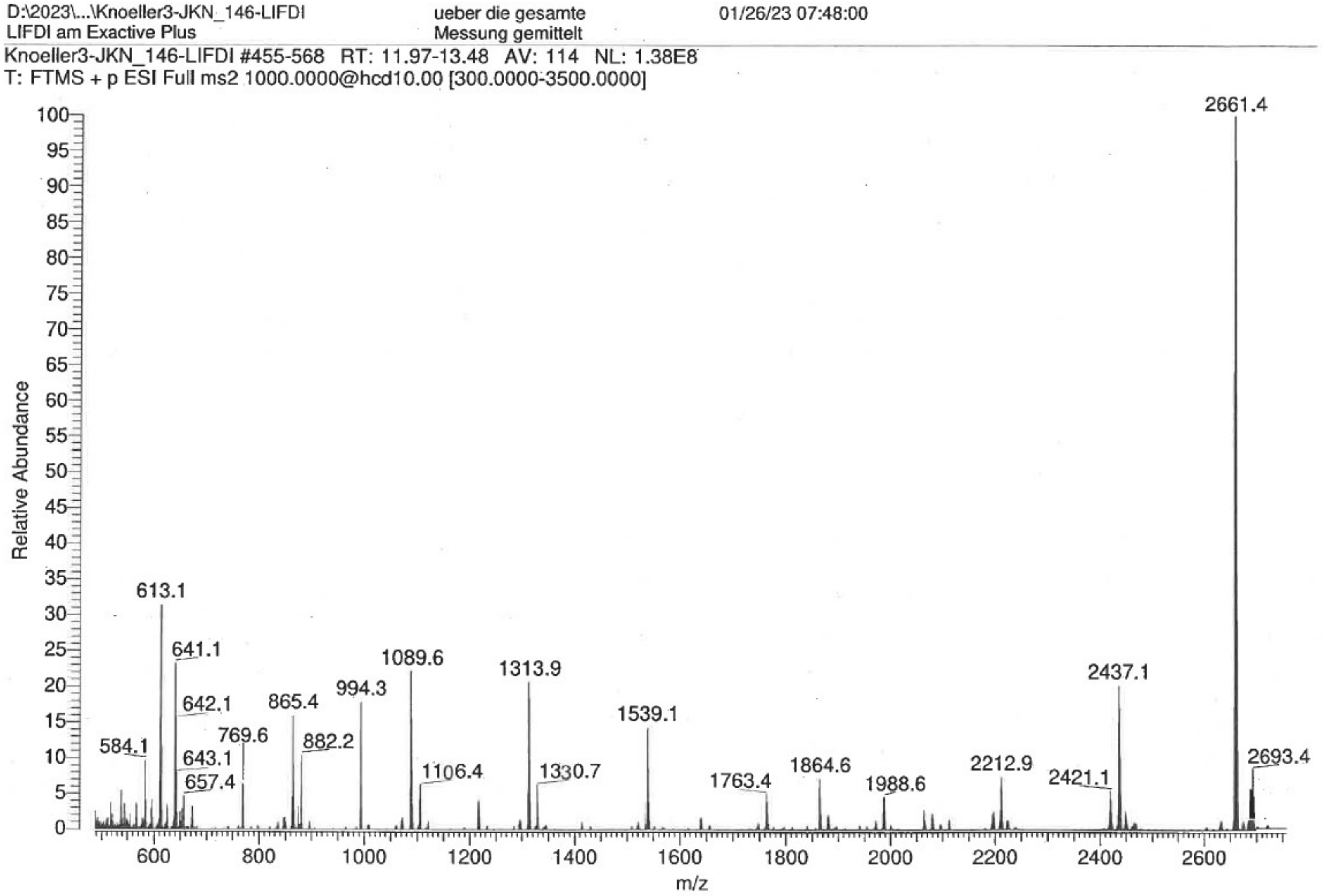


Figure **S24:** LRMS trace of **LC16**.

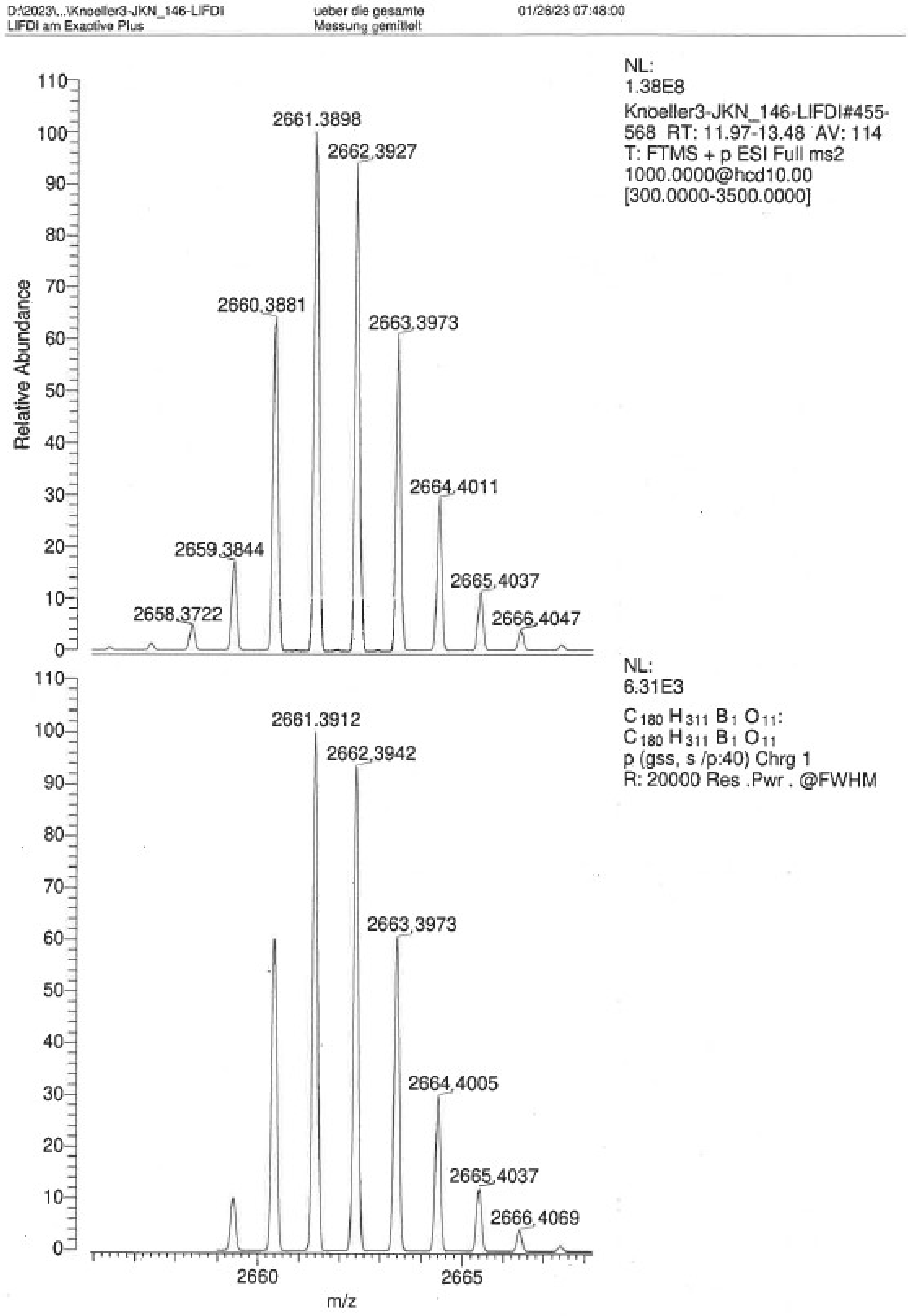


Figure **S25:** HRMS trace of **LC16**.

## 5. Electrochemical Properties of the LCn series

Table **S2:** HOMO and LUMO values of the **LCn** series extracted from DPV measurements and HOMO/ LUMO literature values of **DOBNA** for comparison.

| Compound | $E_{ox}$[a] / V | $E_{HOMO}$ / eV | $E_{red}$[a] / V | $E_{LUMO}$/ eV |
|---|---|---|---|---|
| **LC1** | 1.36 | -6.16 | -1.76 | -3.04 |
| **LC4** | 1.27 | -6.07 | -1.74 | -3.06 |
| **LC8** | 1.26 | -6.06 | -1.74 | -3.06 |
| **LC12** | 1.26 | -6.06 | -1.73 | -3.07 |
| **LC16** | 1.25 | -6.05 | -1.75 | -3.05 |
| **DOBNA**[35] | - | -5.94 | - | -2.79 |
| **BCzBN**[34] | - | -5.55 | - | -2.90 |

[a] referenced vs. standard calomel electrode in a $N_2$ saturated, 0.1 M solution of [$^nBu_4N$]$PF_6$ in $CH_2Cl_2$ via Ferrocene/Ferrocenium ($Fc/Fc^+$) as internal standard (0.46 V vs SCE)[23]; [b] The HOMO and LUMO values were extracted from the anodic / cathodic DPV peak potentials according to $E_{HOMO/LUMO} = -(E_{ox} / E_{red} + 4.8)$ eV.[24]

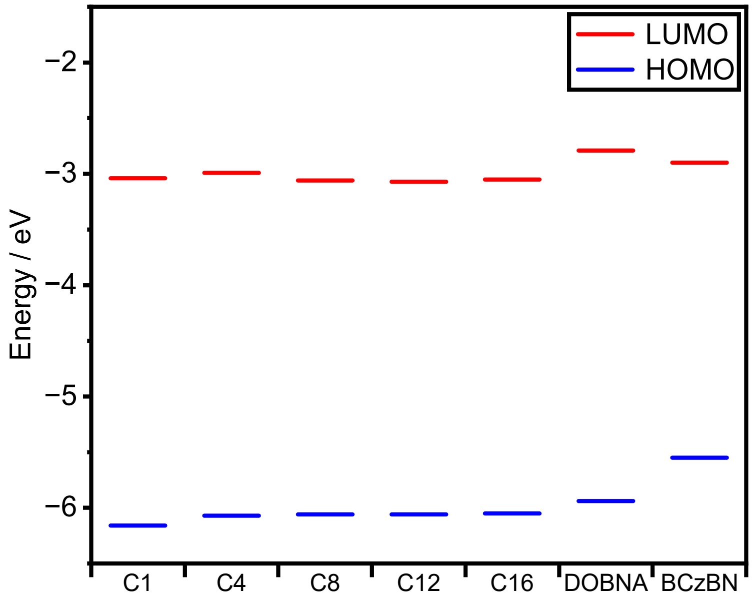


Figure **S26:** HOMO and LUMO values of the **LCn** series extracted from DPV measurements and HOMO/ LUMO literature values of **DOBNA**[35] and **BCzBN**[34] for comparison.

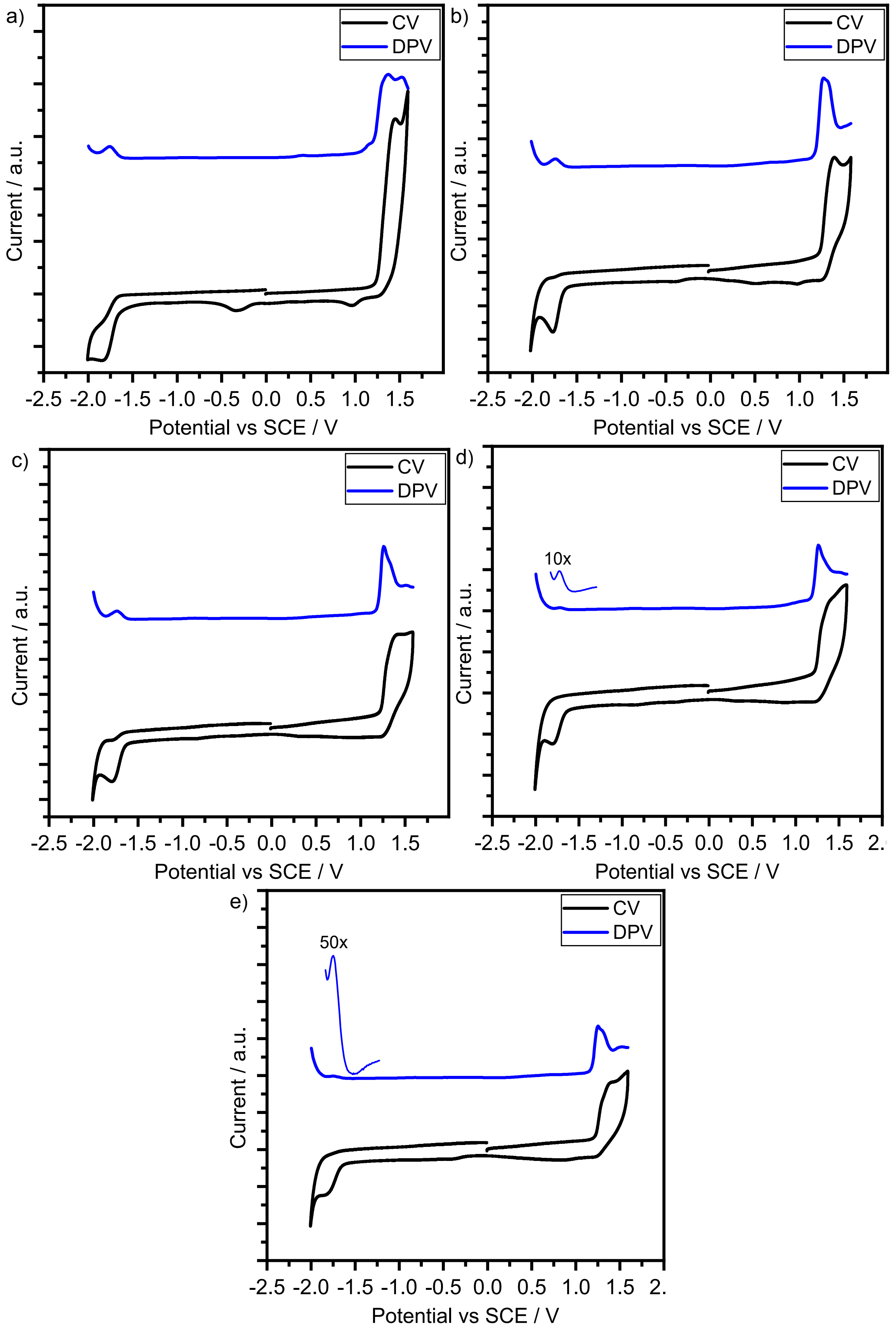


Figure **S27:** CV (black traces) and DPV (blue traces) recorded in $N_2$ saturated, 0.1 M solution of $[^nBu_4N]PF_6$ in $CH_2Cl_2$ vs. Ferrocene/Ferrocenium as internal standard (0.46 V vs SCE,[23] scan rate: 100 mV $s^{-1}$): a) **LC1**, b) **LC4**, c) **LC8**, d) **LC12** (DPV reduction peak magnified by 10×), e) **LC16** (DPV reduction peak magnified by 50×). SCE = standard calomel electrode.

# 6. Mesomorphic Properties

## 6.2. Mesomorphic Properties of the LC Series

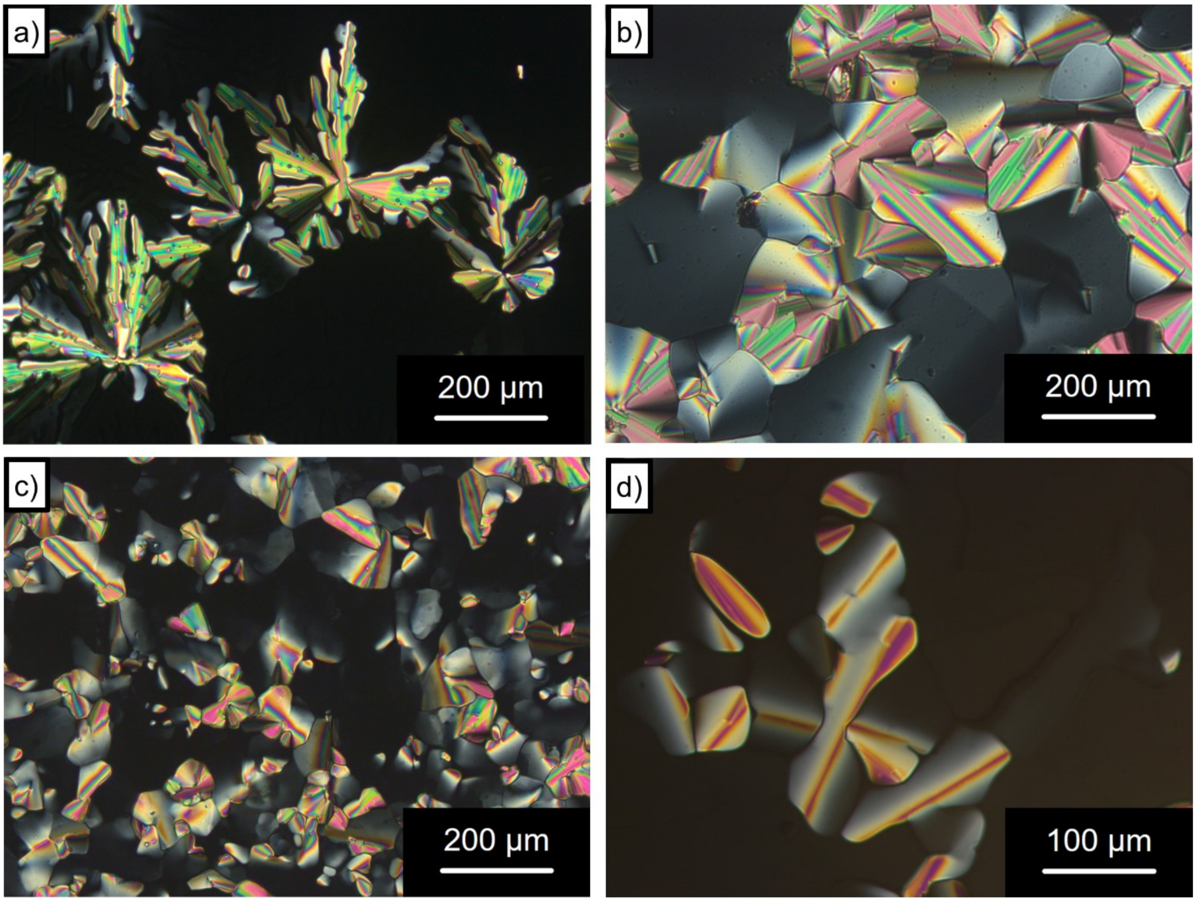


Figure **S28:** Textures obtained on the POM (crossed polarizers, sample sandwiched between two glass slides) upon cooling of: a) **LC4** (160 °C, cooling rate: 5 K $min^{-1}$); b) **LC8** (135 °C, cooling rate: 10 K $min^{-1}$); c) **LC12** (24 °C, cooling rate: 10 K $min^{-1}$); d) **LC16** (86 °C, 0.1 K $min^{-1}$). The black areas correspond to homeotropic alignment of the samples.

Table **S3:** Phase transition temperature *T* / °C and corresponding enthalpies Δ*H* / kJ mol$^{-1}$ of the **DOBNA_LC** series during second heating (2$^{nd}$ H) and cooling (2$^{nd}$ C) cycles in the DSC measurements (heating/ cooling rates: 10 K min$^{-1}$).

| # | | Phase | T / °C (Δ*H* / kJ mol$^{-1}$) | Phase | T / °C (Δ*H* / kJ mol$^{-1}$) | Phase | T / °C (Δ*H* / kJ mol$^{-1}$) | Phase |
|---|---|---|---|---|---|---|---|---|
| **-LC1** | 1$^{st}$ H | Cr | 263.9 (-40.4) | | | Cr | 291.2 (7.67) | I |
| | 1$^{st}$ C [b] | | | | | | | I |
| **-LC4** | 2$^{nd}$ H | G | 44.9 (-12.7) | Cr | 137.9 (-38.4) | $Col_h$ | 174.9 (-2.2) | I |
| | 2$^{nd}$ C | G | 50.0 (-) [a] | | | $Col_h$ | 174.9 (2.0) | I |
| **-LC8** | 2$^{nd}$ H | G | -20 (-) [a] | | | $Col_h$ | 154.1 (-7.3) | I |
| | 2$^{nd}$ C | G | -20 (-) [a] | | | $Col_h$ | 154.6 (6.4) | I |
| **-LC12**[c] | 2$^{nd}$ H | Cr | 7.5 (-29.8) | | | $Col_h$ | 111.9 (-8.4) | I |
| | 2$^{nd}$ C | Cr | 5.3 (28.7) | | | $Col_h$ | 111.7 (8.0) | I |
| **-LC16** | 2$^{nd}$ H | Cr | 49.8 (-152.1) | | | $Col_h$ | 87.4 (-9.1) | I |
| | 2$^{nd}$ C | Cr | 43.9 (79.4) | | | $Col_h$ | 86.4 (7.5) | I |

[a]: Vitrification determined via slow cooling (cooling rate: 1 K min$^{-1}$ under the POM). [b]: no transition observed, presumably due to decomposition of the sample.

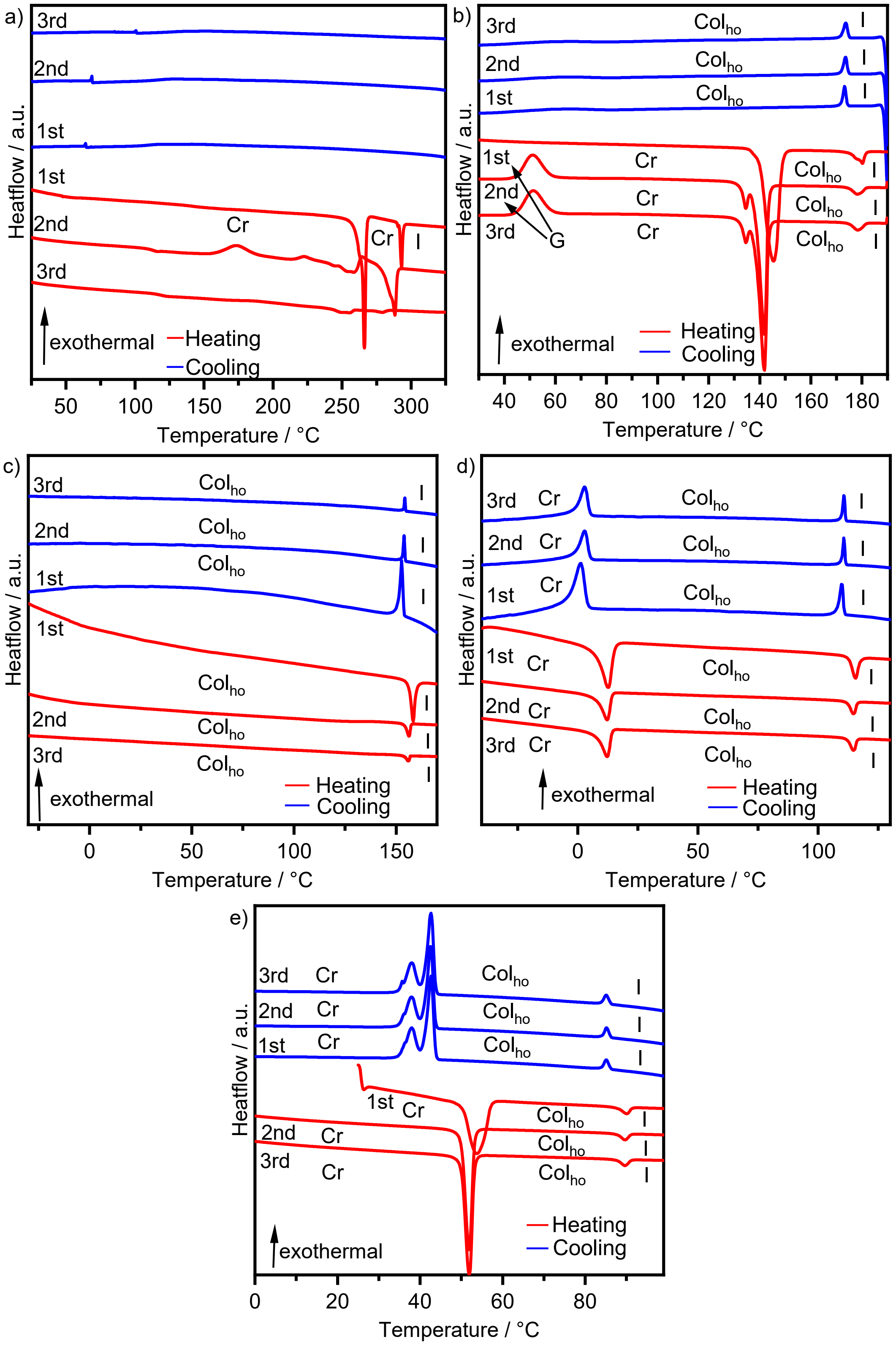


Figure **S29:** DSC thermograms (heating/ cooling rate: 10 K min$^{-1}$) of: a) **LC1** b) **LC4**; c) **LC8**; d) **LC12**; e) **LC16**.

**Table S4:** XRD data of the **DOBNA_LC** series.

| # | Mesophase | Lattice parameters | *d* / Å [a] | Miller indices |
|---|---|---|---|---|
| **LC4** | $Col_h$ at 144 °C<br>*p6 mm* | *a* = 25.50 Å<br>*Z* = 1 | 22.05<br>12.54 (12.73)<br>- (11.02) [b]<br>8.30 (8.33)<br>4.41<br>3.48 | (10)<br>(11)<br>(20)<br>(21)<br>(halo)<br>(π-π) |
| **LC8** | $Col_h$ at 29 °C<br>*p6 mm* | *a* = 31.41 Å<br>*Z* = 1 | 27.20<br>15.71 (15.70)<br>13.63 (13.60)<br>10.28 (10.28)<br>4.26<br>3.59 | (10)<br>(11)<br>(20)<br>(21)<br>(halo)<br>(π-π) |
| **LC12**[c] | $Col_h$ at 29 °C<br>*p6 mm* | *a* = 37.80 Å<br>*Z* = 1 | 32.72<br>4.32<br>3.62 | (10)<br>(halo)<br>(π-π) |
| **LC16** | $Col_h$ at 68 °C<br>*p6 mm* | *a* = 40.1 Å<br>*Z* = 1 | 34.71<br>19.95 (20.04)<br>4.43<br>3.67 | (10)<br>(11)<br>(halo)<br>(π-π) |

[a]: Calculated d values are given in parentheses. [b]: Reflection not observed. [c]: Mesophase assigned via POM as no additional SAXS reflections were detected.

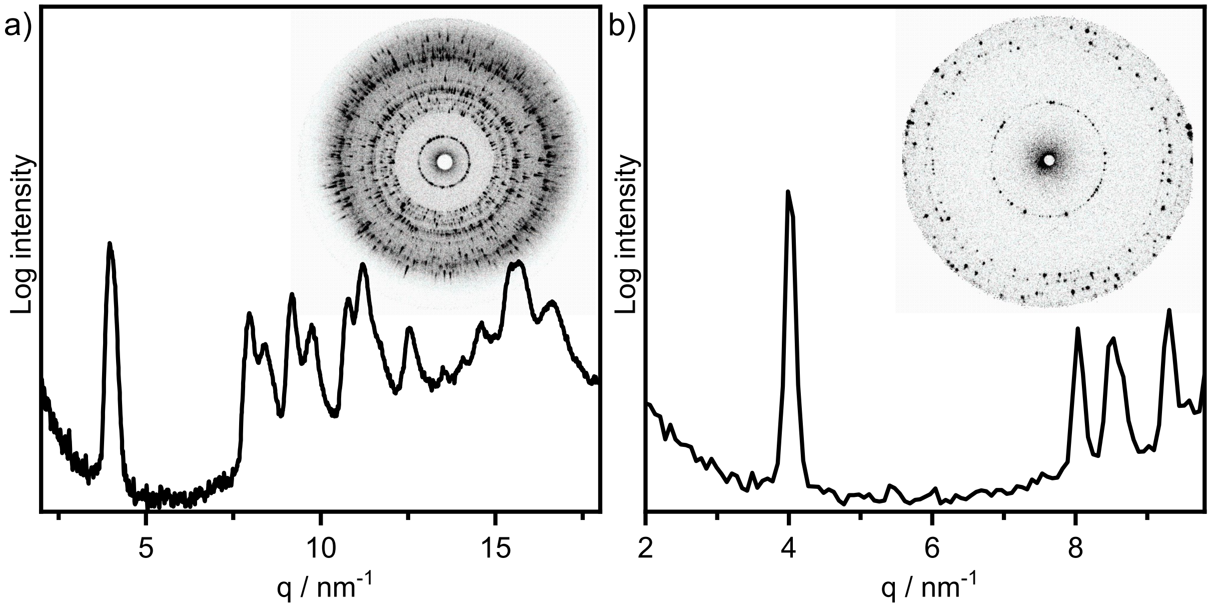


Figure **S30:** a) WAXS diffractogram of **LC1** recorded at 280 °C with the 2D WAXS pattern shown as inset; b) SAXS diffractogram of **LC1** recorded at XX °C with the 2D SAXS pattern shown as inset

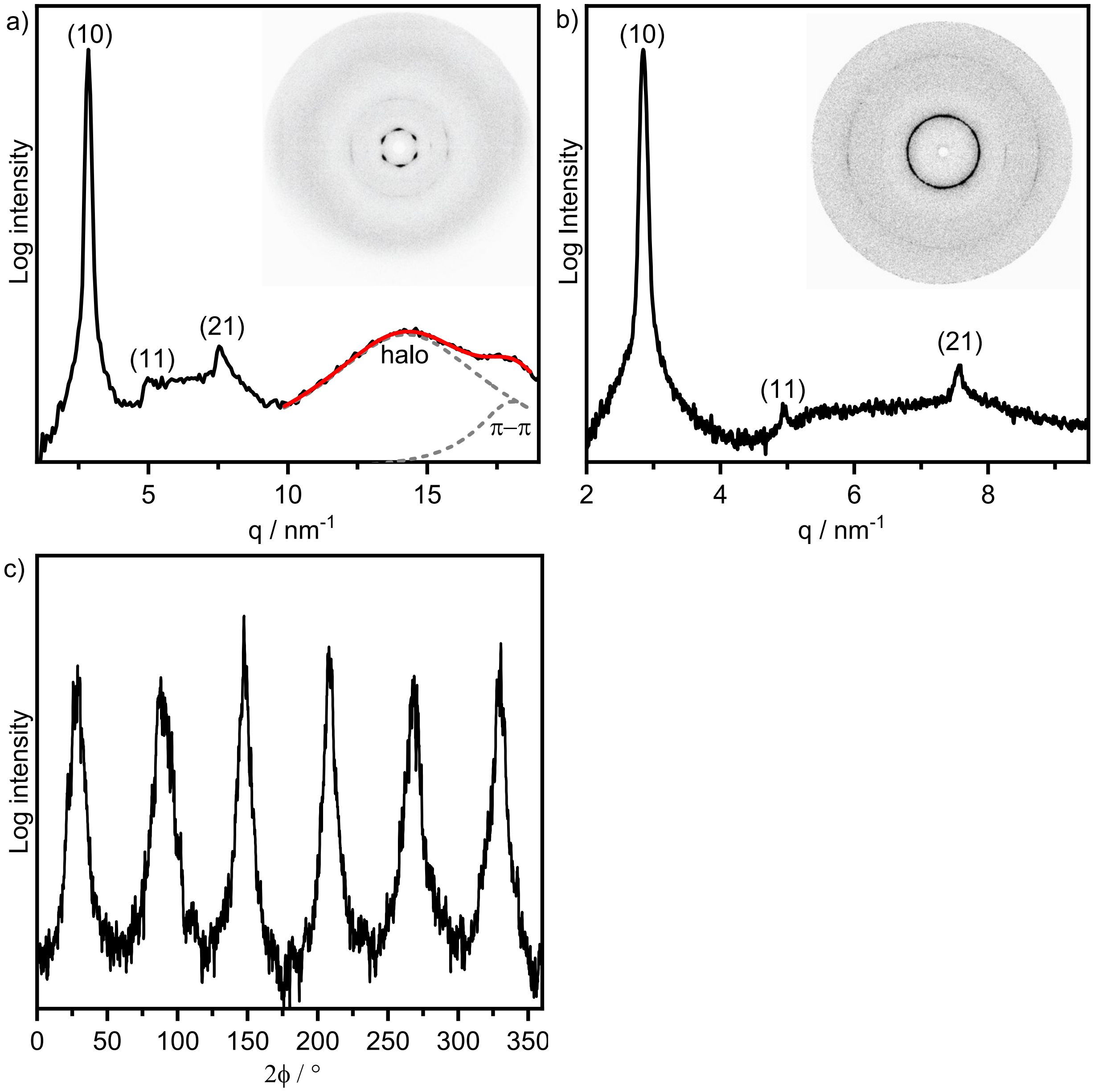


Figure **S31:** a) WAXS diffractogram of **LC4** recorded at 144 °C with the 2D WAXS pattern shown as inset. The red trace represents the fit of the wide-angle region with two Lorentz functions (grey dashed traces); b) SAXS diffractogram of **LC4** recorded at 144 °C with the 2D SAXS pattern shown as inset; c) Azimuthal integration of the (10) reflection in the WAXS pattern of **LC4** recorded at 144 °C displaying the characteristic hexagon of a $Col_h$ phase.

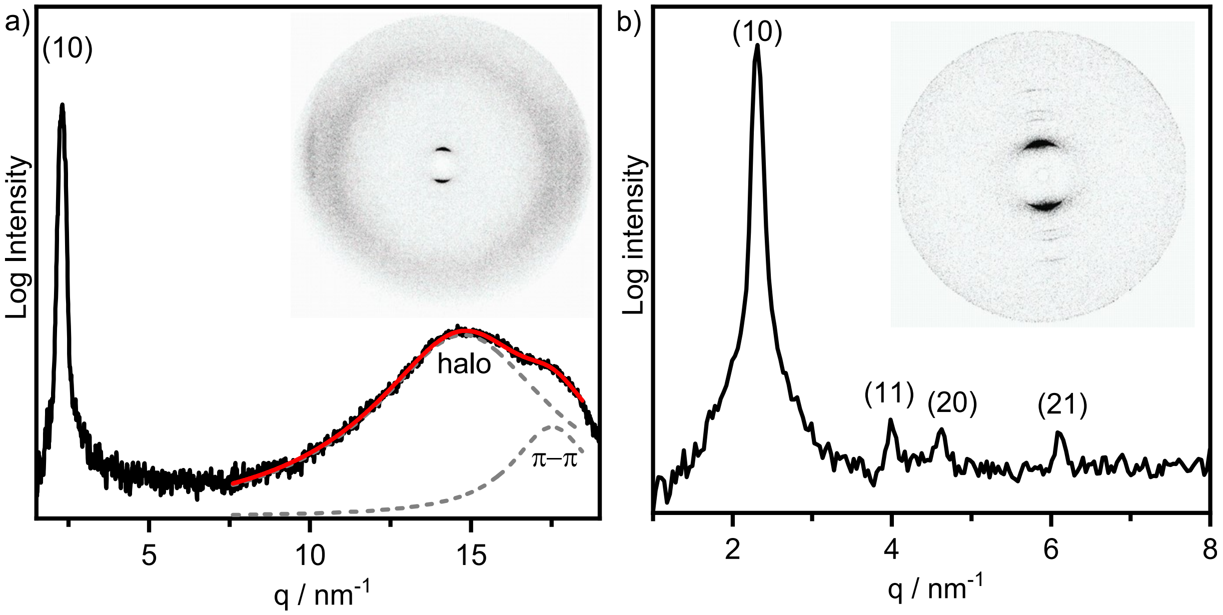


Figure **S32:** a) WAXS diffractogram of **LC8** recorded at 29 °C with the 2D WAXS pattern shown as inset. The red trace represents the fit of the wide-angle region with two Lorentz functions (grey dashed traces); b) SAXS diffractogram of **LC8** recorded at 29 °C with the 2D SAXS pattern shown as inset.

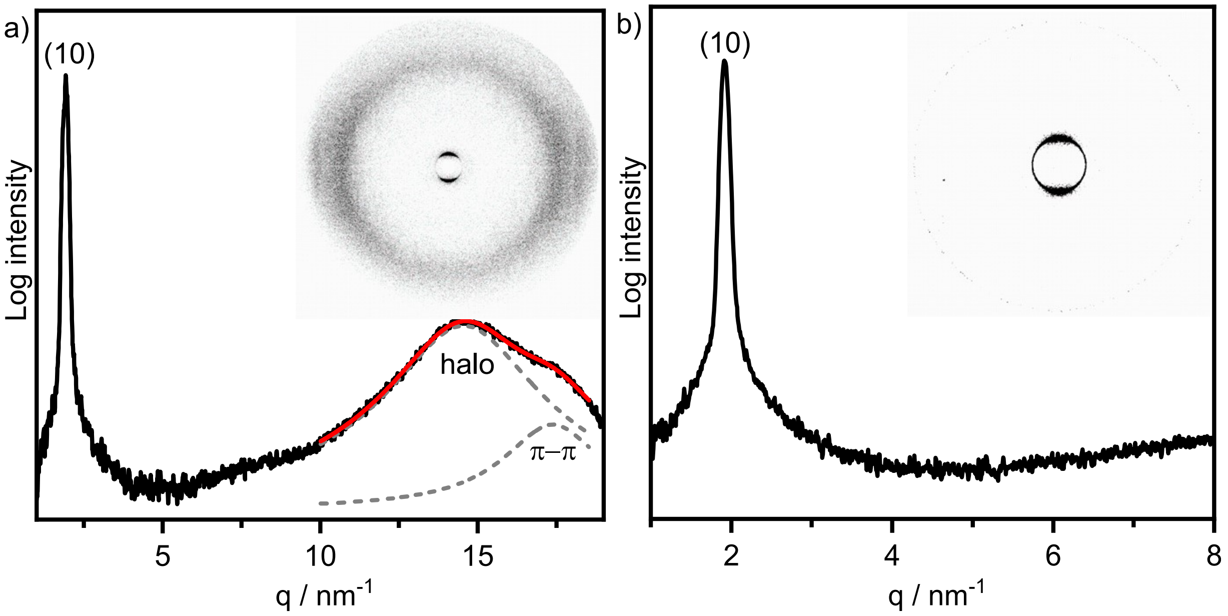


Figure **S33:** a) WAXS diffractogram of **LC12** recorded at 29 °C with the 2D WAXS pattern shown as inset. The red trace represents the fit of the wide-angle region with two Lorentz functions (grey dashed traces); b) SAXS diffractogram of **LC12** recorded at 29 °C with the 2D SAXS pattern shown as inset.

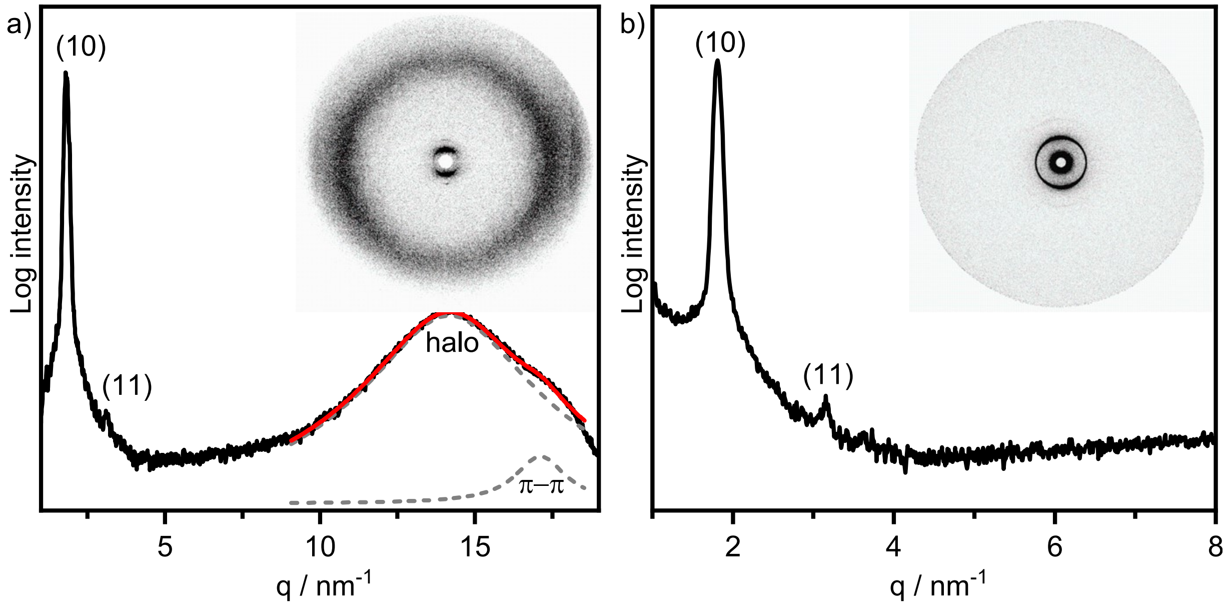


Figure **S34:** a) WAXS diffractogram of **LC16** recorded at 68 °C with the 2D WAXS pattern shown as inset. The red trace represents the fit of the wide-angle region with two Lorentz functions (grey dashed traces); b) SAXS diffractogram of **LC16** recorded at 68 °C with the 2D SAXS pattern shown as inset.

### 6.3. Mesomorphic Properties of the LCn series doped with BCzBN (1wt%)

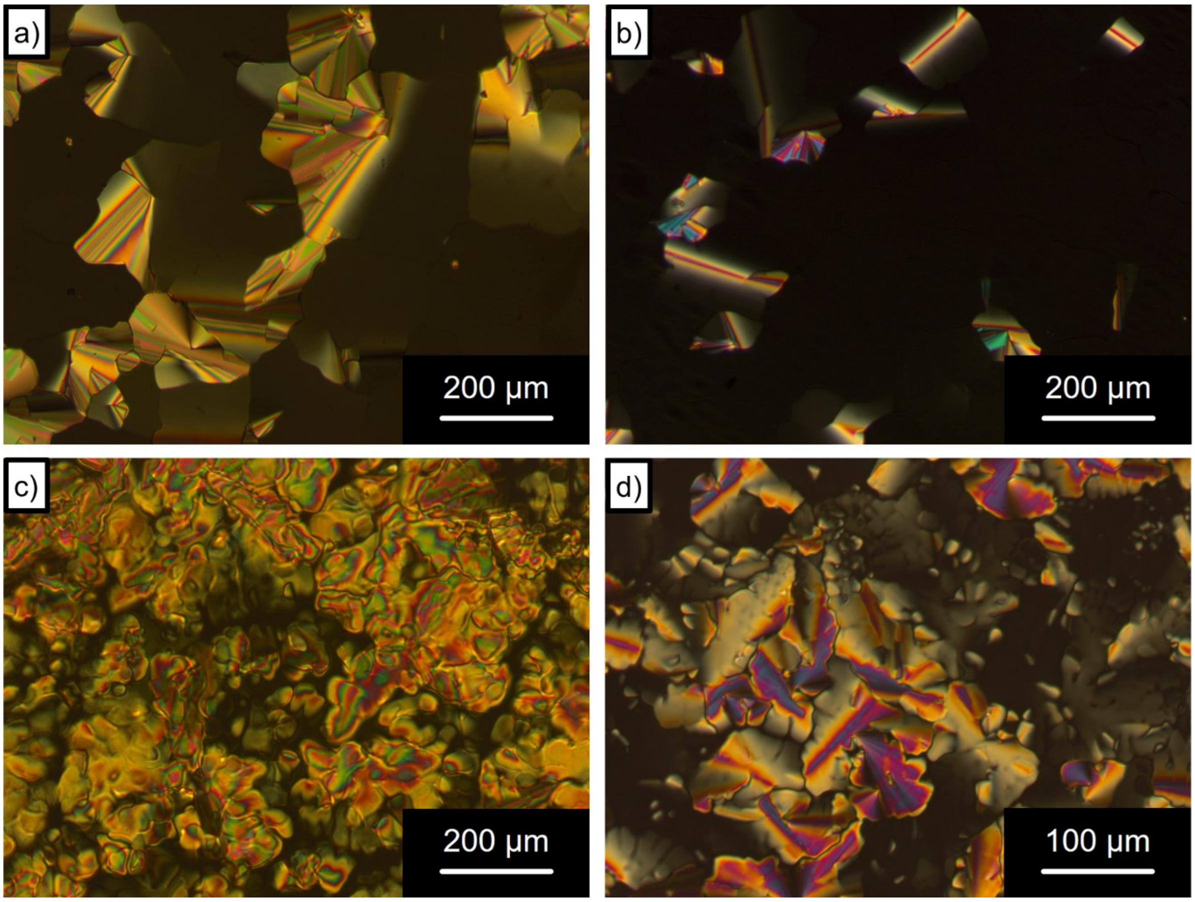


Figure **S35:** Textures obtained on the POM (crossed polarizers, sample sandwiched between two glass slides) upon cooling of the respective **LCn** doped with **BCzBN** (1 wt%): a) **LC4** (160 °C, cooling rate: 1 K $min^{-1}$); b) **LC8** (135 °C, cooling rate: 1 K $min^{-1}$); c) **LC12** (24 °C, cooling rate: 1 K $min^{-1}$); d) **LC16** (86 °C, 1 K $min^{-1}$). The black areas correspond to homeotropic alignment of the samples.

Table **S5:** Phase transition temperature *T* / °C and corresponding enthalpies Δ*H* / kJ mol$^{-1}$ of the **LCn** series doped with **BCzBN** during second heating (2$^{nd}$ H) and cooling (2$^{nd}$ C) cycles in the DSC measurements (heating/ cooling rates: 10 K min$^{-1}$).

| # | | Phase | T / °C (Δ*H* / kJ mol$^{-1}$) | Phase | T / °C (Δ*H* / kJ mol$^{-1}$) | Phase | T / °C (Δ*H* / kJ mol$^{-1}$) | Phase |
|---|---|---|---|---|---|---|---|---|
| **-LC1** | 1$^{st}$ H | | | | | Cr | 287 (-45.7) | I |
| | 1$^{st}$ C [b] | | | | | | | I |
| **-LC4** | 2$^{nd}$ H | G | 50.0 (-13.8) | Cr | 137.1 (-34.8) | $Col_h$ | 165.0 (-1.4) | I |
| | 2$^{nd}$ C | G | 50 (-) [a] | | | $Col_h$ | 172.5 (1.7) | I |
| **-LC8** | 2$^{nd}$ H | G | -20 (-) [a] | | | $Col_h$ | 150.3 (-6.3) | I |
| | 2$^{nd}$ C | G | -20 (-) [a] | | | $Col_h$ | 152.3 (6.3) | I |
| **-LC12**[c] | 2$^{nd}$ H | Cr | 6.0 (-20.3) | | | $Col_h$ | 107.9 (-6.2) | I |
| | 2$^{nd}$ C | Cr | 5.9 (21.1) | | | $Col_h$ | 109.6 (7.4) | I |
| **-LC16** | 2$^{nd}$ H | Cr | 49.3 (-111.8) | | | $Col_h$ | 86.7 (-7.2) | I |
| | 2$^{nd}$ C | Cr | 43.7 (113.3) | | | $Col_h$ | 86.4 (6.0) | I |

[a]: Vitrification determined via slow cooling (cooling rate: 1 K min$^{-1}$ under the POM. [b]: no transition observed, presumably due to decomposition of the sample.

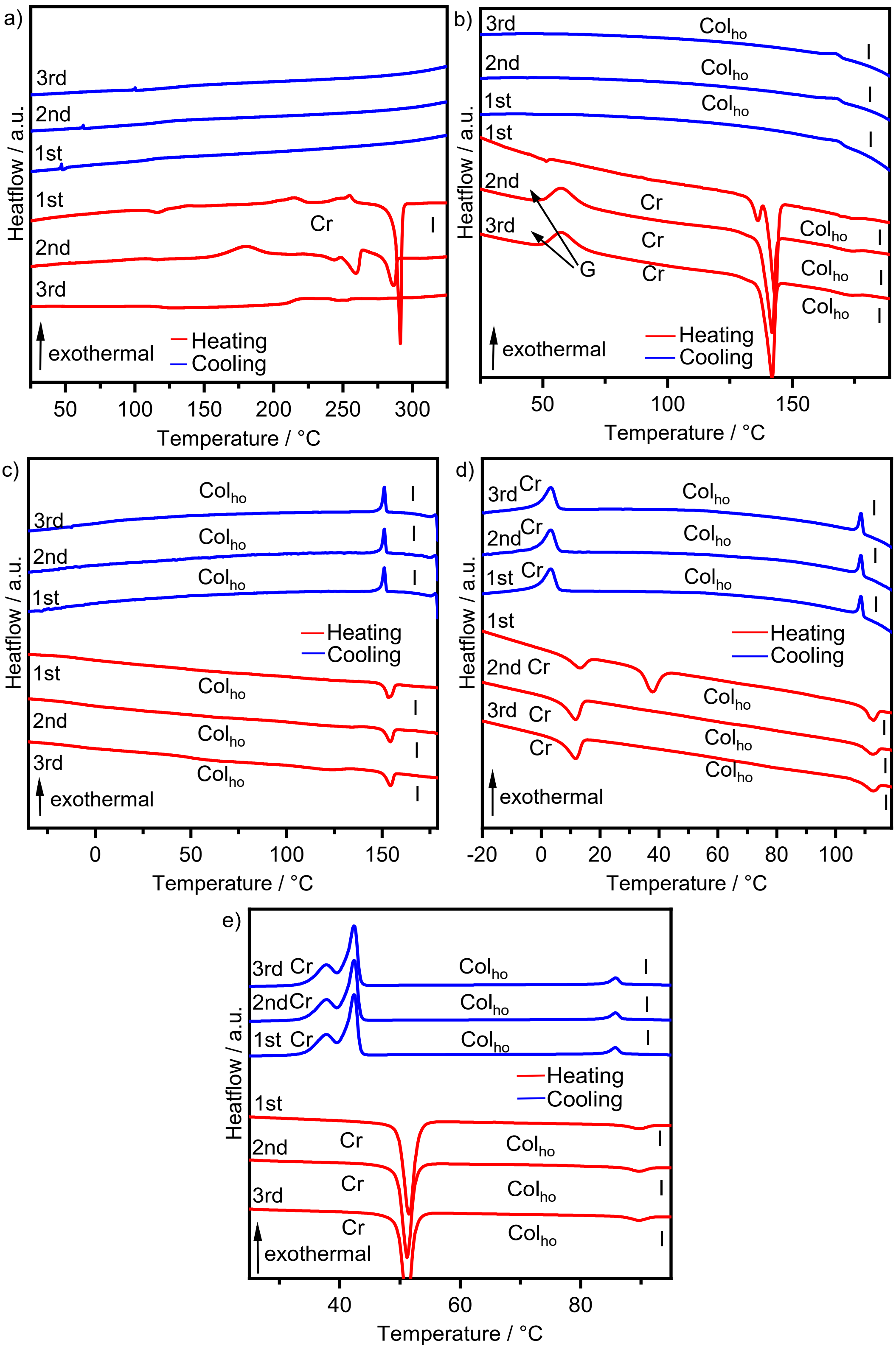


Figure **S36:** DSC thermograms (heating/ cooling rate: 10 K min$^{-1}$) of the respective **LC** derivative doped with **BCzBN** (1 wt%): a) **LC1** b) **LC4**; c) **LC8**; d) **LC12**; e) **LC16**.

Table **S6:** XRD data of the **LCn** series doped with **BCzBN** (1 wt%).

| # | Mesophase | Lattice parameters | $d$ / Å [a] | Miller indices |
|---|---|---|---|---|
| **LC4** | Col$_h$ at 144 °C<br>*p6 mm* | $a$ = 25.30 Å<br>$Z$ = 1<br>$\rho$ = 0.77 | 21.89<br>12.62 (12.64)<br>- (10.95) [b]<br>8.30 (8.27)<br>4.51<br>3.54 | (10)<br>(11)<br>(20)<br>(21)<br>(halo)<br>(π-π) |
| **LC8** | Col$_h$ at 29 °C<br>*p6 mm* | $a$ = 31.40 Å<br>$Z$ = 1<br>$\rho$ = 0.74 | 27.20<br>15.59 (15.70)<br>13.60 (13.60)<br>10.25 (10.28)<br>4.36<br>3.57 | (10)<br>(11)<br>(20)<br>(21)<br>(halo)<br>(π-π) |
| **-C12**[c] | Col$_h$ at 29 °C<br>*p6 mm* | $a$ = 37.21 Å<br>$Z$ = 1<br>$\rho$ = 0.68 | 32.22<br>4.41<br>3.58 | (10)<br>(halo)<br>(π-π) |
| **LC16** | Col$_h$ at 68 °C<br>*p6 mm* | $a$ = 39.43 Å<br>$Z$ = 1<br>$\rho$ = 0.63 | 34.15<br>19.88 (19.72)<br>4.53<br>3.57 | (10)<br>(11)<br>(halo)<br>(π-π) |

[a]: Calculated d values are given in parentheses. [b]: Reflection not observed. [c]: Mesophase assigned via POM as no additional SAXS reflections were detected.

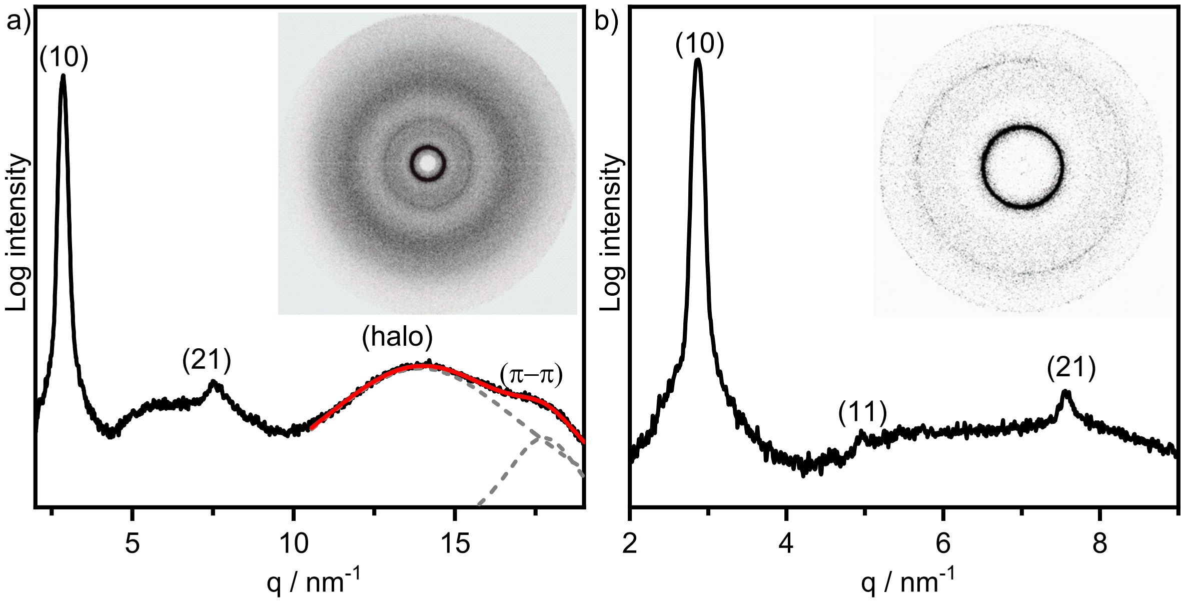


Figure **S37:** a) WAXS diffractogram of **LC4** doped with **BCzBN** (1 wt%) recorded at 144 °C with the 2D WAXS pattern shown as inset. The red trace represents the fit of the wide-angle region with two Lorentz functions (grey dashed traces); b) SAXS diffractogram of **LC4** doped with **BCzBN** (1 wt%) recorded at 144 °C with the 2D SAXS pattern shown as inset.

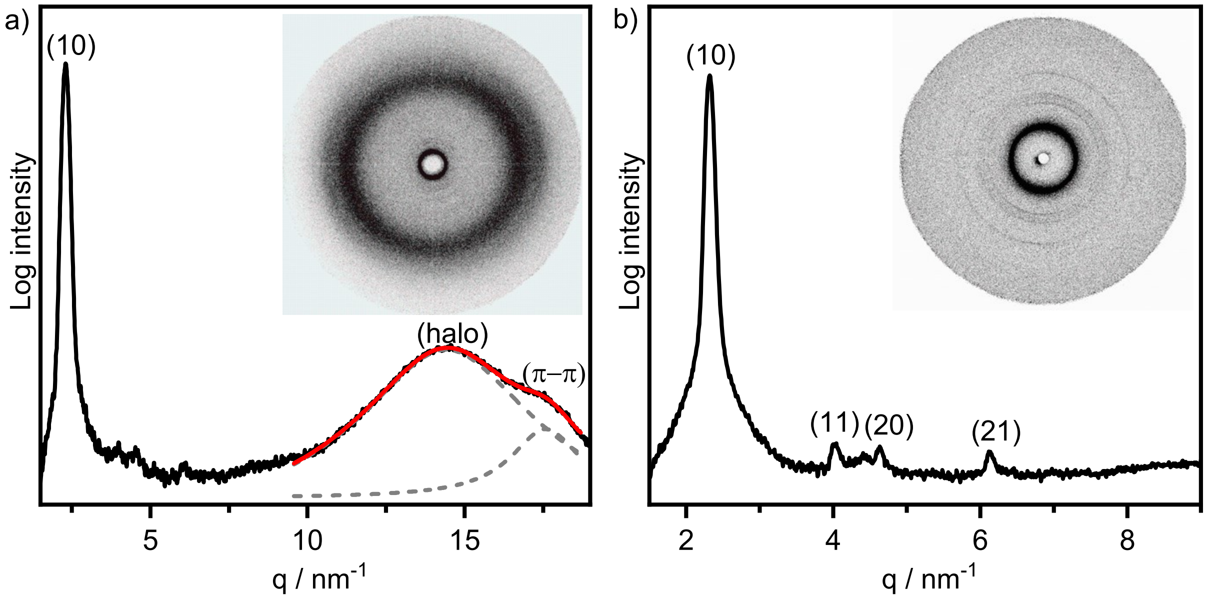


Figure **S38:** a) WAXS diffractogram of **LC8** doped with **BCzBN** (1 wt%) recorded at 29 °C with the 2D WAXS pattern shown as inset. The red trace represents the fit of the wide-angle region with two Lorentz functions (grey dashed traces); b) SAXS diffractogram of **LC8** doped with **BCzBN** (1 wt%) recorded at 29 °C with the 2D SAXS pattern shown as inset.

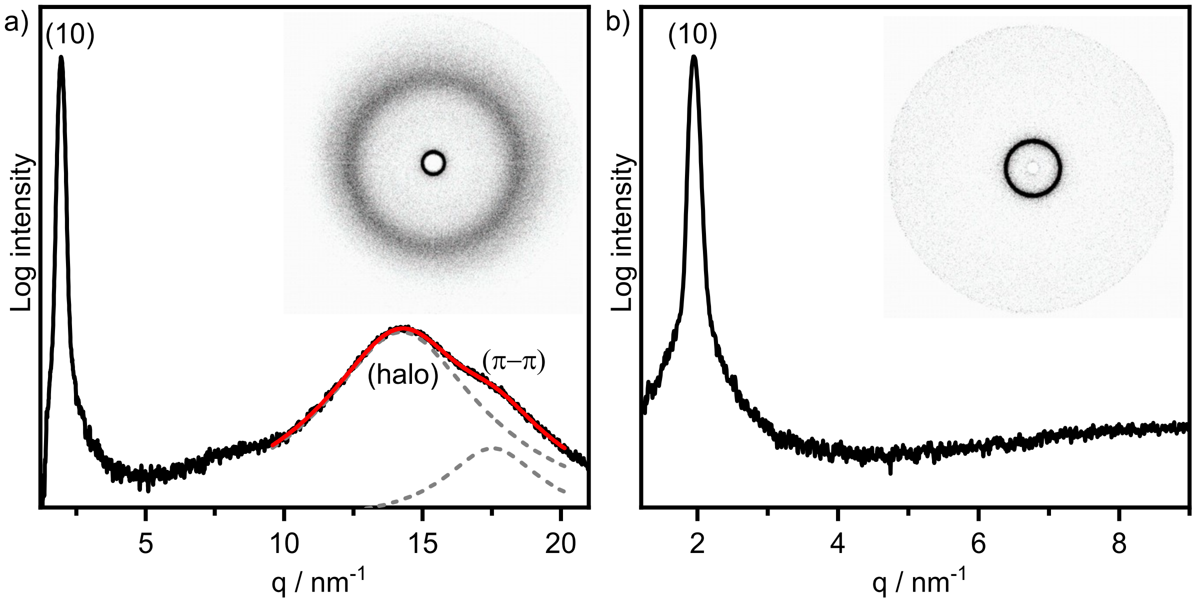


Figure **S39:** a) WAXS diffractogram of **LC12** doped with **BCzBN** (1 wt%) recorded at 29 °C with the 2D WAXS pattern shown as inset. The red trace represents the fit of the wide-angle region with two Lorentz functions (grey dashed traces); b) SAXS diffractogram of **LC12** doped with **BCzBN** (1 wt%) recorded at 29 °C with the 2D SAXS pattern shown as inset.

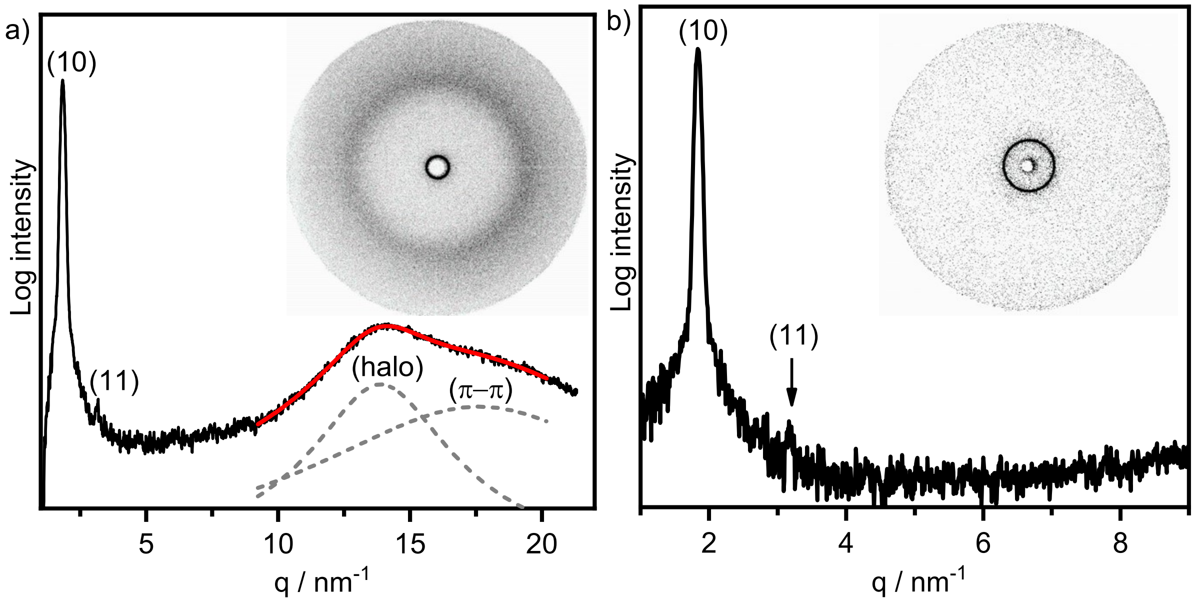


Figure **S40:** a) WAXS diffractogram of **LC16** doped with **BCzBN** (1 wt%) recorded at 68 °C with the 2D WAXS pattern shown as inset. The red trace represents the fit of the wide-angle region with two Lorentz functions (grey dashed traces); b) SAXS diffractogram of **LC16** doped with **BCzBN** (1 wt%) recorded at 68 °C with the 2D SAXS pattern shown as inset.

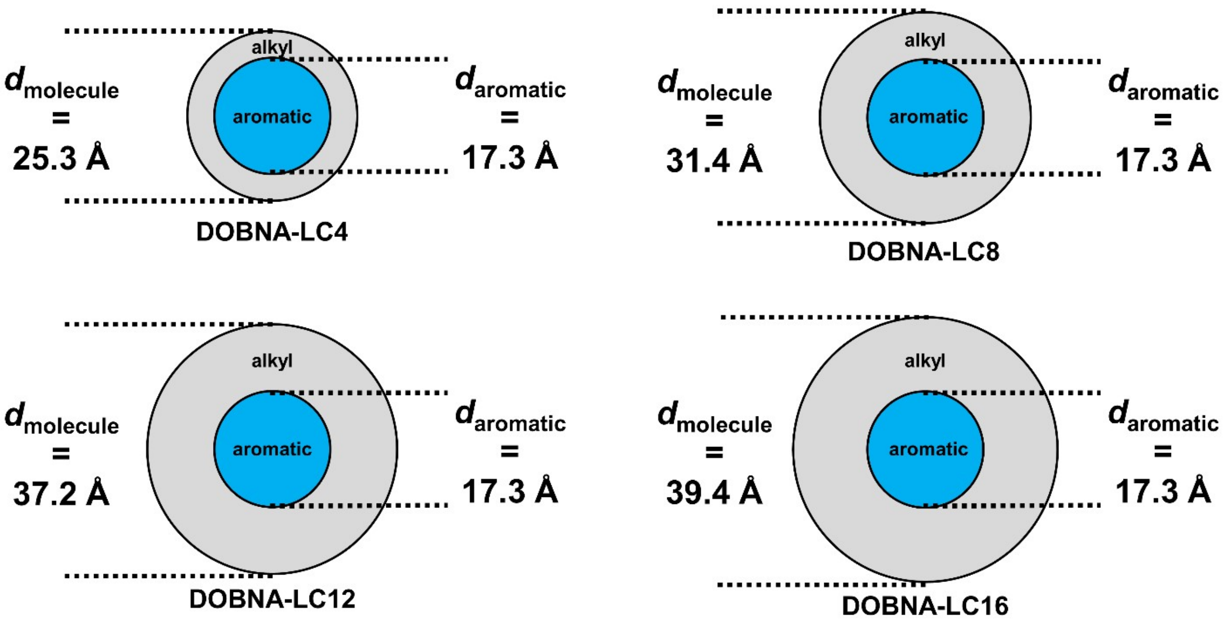


Figure **S41:** Schematic model of **LC4** – **LC16** as discotic molecules derived from doped (**BCzBN**, c = 1 wt%) SAXS data ($d_{\text{molecule}} = a$) and DFT calculations ($d_{\text{aromatic}}$) showcasing the growing non-polar alkyl-part with increasing chain length of **LC**.

# 7. Photophysical Properties

## 7.2. Photophysical Properties of the LC Series in Solution

Table **S7:** Photophysical data of the **LC** series in dilute toluene solution (c = 0.02 mM, steady-state: $\lambda_{exc}$ = 350 nm, time-resolved: $\lambda_{exc}$ = 373 nm, $\Delta E_{ST}$**:** $\lambda_{exc}$ = 343 nm).

| # | $\lambda_{abs}$ / nm ($\varepsilon$ / $10^4$ $M^{-1}$ $cm^{-1}$) | $\lambda_{PL}$ / nm | FWHM / nm (FWHM / meV) | Stokes shift / meV (stokes shift / $cm^{-1}$) | $\Phi_{PL}$ / %. | $\tau_P$ / ns | $\Delta E_{ST}$ / eV |
|---|---|---|---|---|---|---|---|
| **LC1** | 339 (6.1)<br>393 (4.5) | 407 | 25 (183.2) | 108.5<br>(875.3) | 58 | 3.99 | 0.45 |
| **LC4** | 342 (4.8)<br>393 (3.8) | 408 | 23 (168.5) | 116.0<br>(935.5) | 62 | 3.73 | 0.44 |
| **LC8** | 340 (3.9)<br>392 (3.0) | 408 | 24 (176.3) | 124.0<br>(1000.4) | 63 | 3.65 | 0.46 |
| **LC12** | 340 (4.9)<br>392 (3.8) | 408 | 23 (168.5) | 124.0<br>(1000.4) | 60 | 3.66 | -* |
| **LC16** | 340 (5.2)<br>392 (4.0) | 407 | 25 (183.2) | 116.6<br>(940.2) | 60 | 3.66 | -* |

*$\Delta E_{ST}$ could not be determined reliably, as sample aggregated while rapidly freezing the dilute toluene solution with liquid $N_2$.

Table **S8:** Solvatochromism data of the **LC** series in solvents of different polarity (c = 0.02 mM, $\lambda_{exc}$ = 350 nm).

| solvent | Methylcyclohexane | | Toluene | | $CH_2Cl_2$ | | THF | | Butyronitrile | |
|---|---|---|---|---|---|---|---|---|---|---|
| $E_T(30)$ / kcal · $mol^{-1}$ | ~ 31[a] | | 33.9[33] | | 37.4[36] | | 40.7[36] | | 42.5[36] | |
| # | $\lambda_{abs}$ / nm | $\lambda_{PL}$ / nm | $\lambda_{abs}$ / nm | $\lambda_{PL}$ / nm | $\lambda_{abs}$ / nm | $\lambda_{PL}$ / nm | $\lambda_{abs}$ / nm | $\lambda_{PL}$ / nm | $\lambda_{abs}$ / nm | $\lambda_{PL}$ / nm |
| **LC1** | 335<br>391 | 401 | 339<br>393 | 407 | 337<br>391 | 411 | 339<br>392 | 410 | 337<br>391 | 410 |
| **LC4** | 339<br>392 | 401 | 339<br>392 | 408 | 340<br>392 | 410<br>478 | 342<br>393 | 407 | 340<br>392 | 411<br>507 |
| **LC8** | 339<br>392 | 402 | 340<br>392 | 408 | 340<br>391 | 411<br>478 | 342<br>393 | 408 | 341<br>392 | 411<br>503 |
| **LC12** | 339<br>392 | 402 | 340<br>392 | 408 | 340<br>391 | 411<br>478 | 342<br>393 | 408 | 341<br>392 | 410<br>508 |
| **LC16** | 339<br>392 | 401 | 340<br>392 | 407 | 340<br>392 | 410<br>478 | 342<br>393 | 407 | 341<br>392 | 401<br>500 |

[a]: no $E_T(30)$ value reported for methylcyclohexane. Other aliphatics have an $E_T(30)$ ~ 31 kcal · $mol^{-1}$ and this value is given as rough estimate for methylcyclohexane.[36]

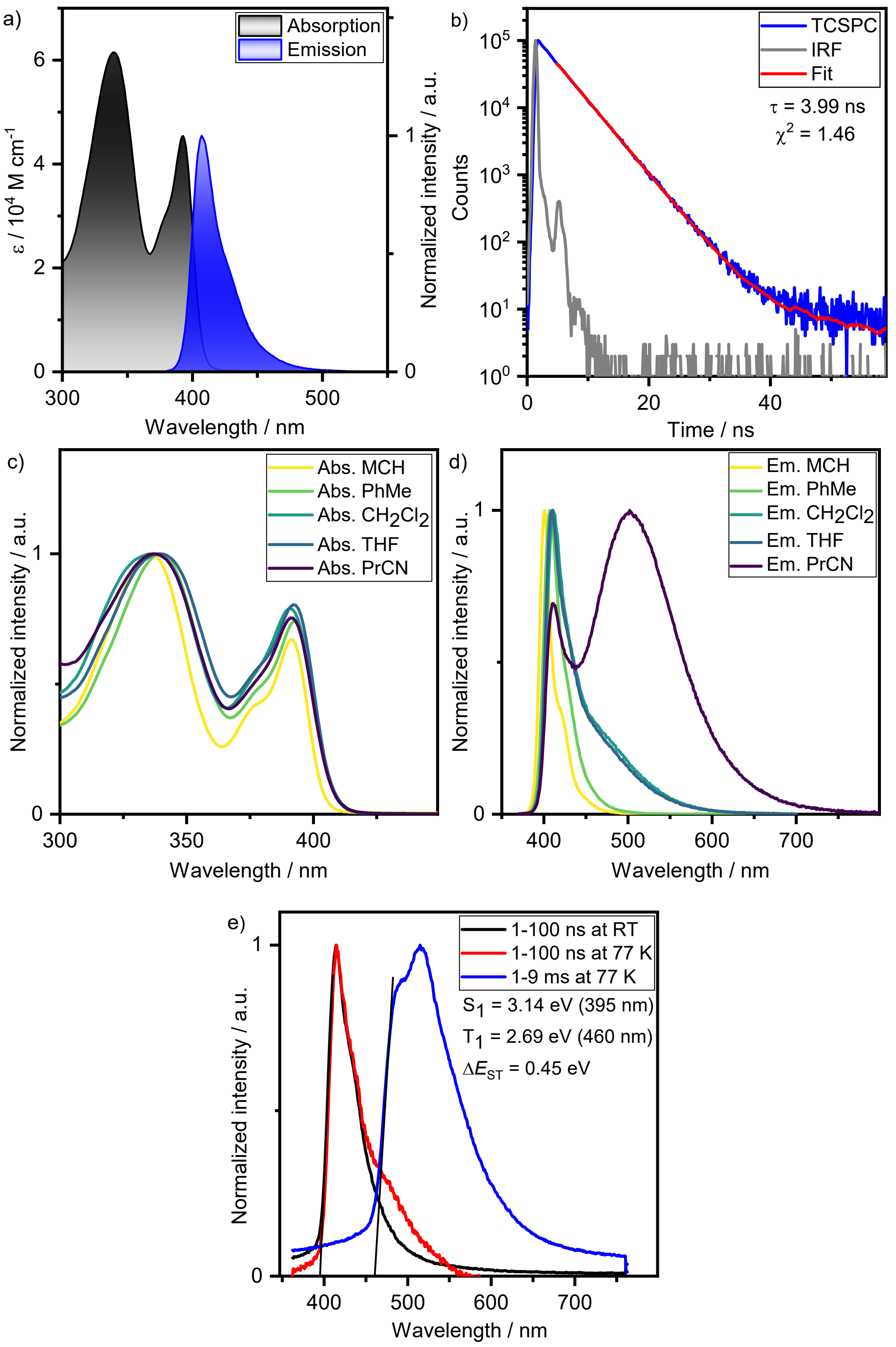


Figure **S42:** Solution photophysics of **LC1** in degassed toluene (c = 0.02 mM): a) steady-state absorption (black trace) and emission (blue trace) ($\lambda_{exc}$ = 350 nm); b) luminescence decay (blue trace) with monoexponential fit (red trace) and IRF (grey trace) in the ns range ($\lambda_{exc}$ = 373 nm); c) solvatochromism in solvents of different polarity; d) solvatofluorochromism in solvents of different polarity; e) fluorescence (red trace), phosphorescence (blue trace) and corresponding onsets as $S_1$ and $T_1$ energies at 77K in a frozen toluene matrix ($\lambda_{exc}$ = 343 nm). MCH = methylcyclohexane, PhMe = toluene, PrCN = butyronitrile.

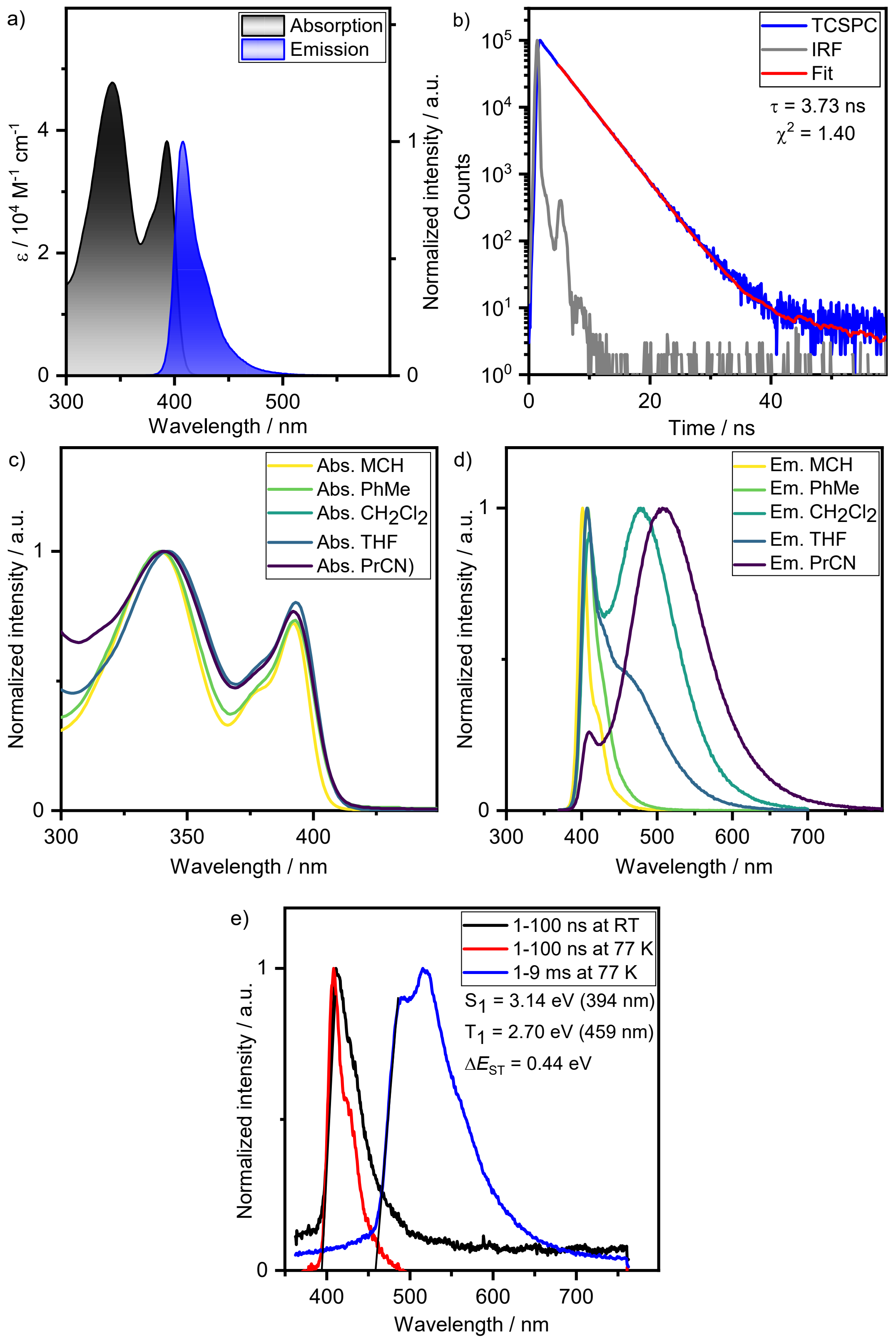


Figure **S43:** Solution-state photophysics of **LC4** in degassed toluene (c = 0.02 mM): a) steady-state absorption (black trace) and emission (blue trace) ($\lambda_{exc}$ = 350 nm); b) luminescence decay (blue trace) with monoexponential fit (red trace) and IRF (grey trace) in the ns range ($\lambda_{exc}$ = 373 nm); c) solvatochromism in solvents of different polarity; d) solvatochromism in solvents of different polarity; e) fluorescence (red trace), phosphorescence (blue trace) and corresponding onsets as $S_1$ and $T_1$ energies at 77K in a frozen toluene matrix ($\lambda_{exc}$ = 343 nm). MCH = methylcyclohexane, PhMe = toluene, PrCN = butyronitrile.

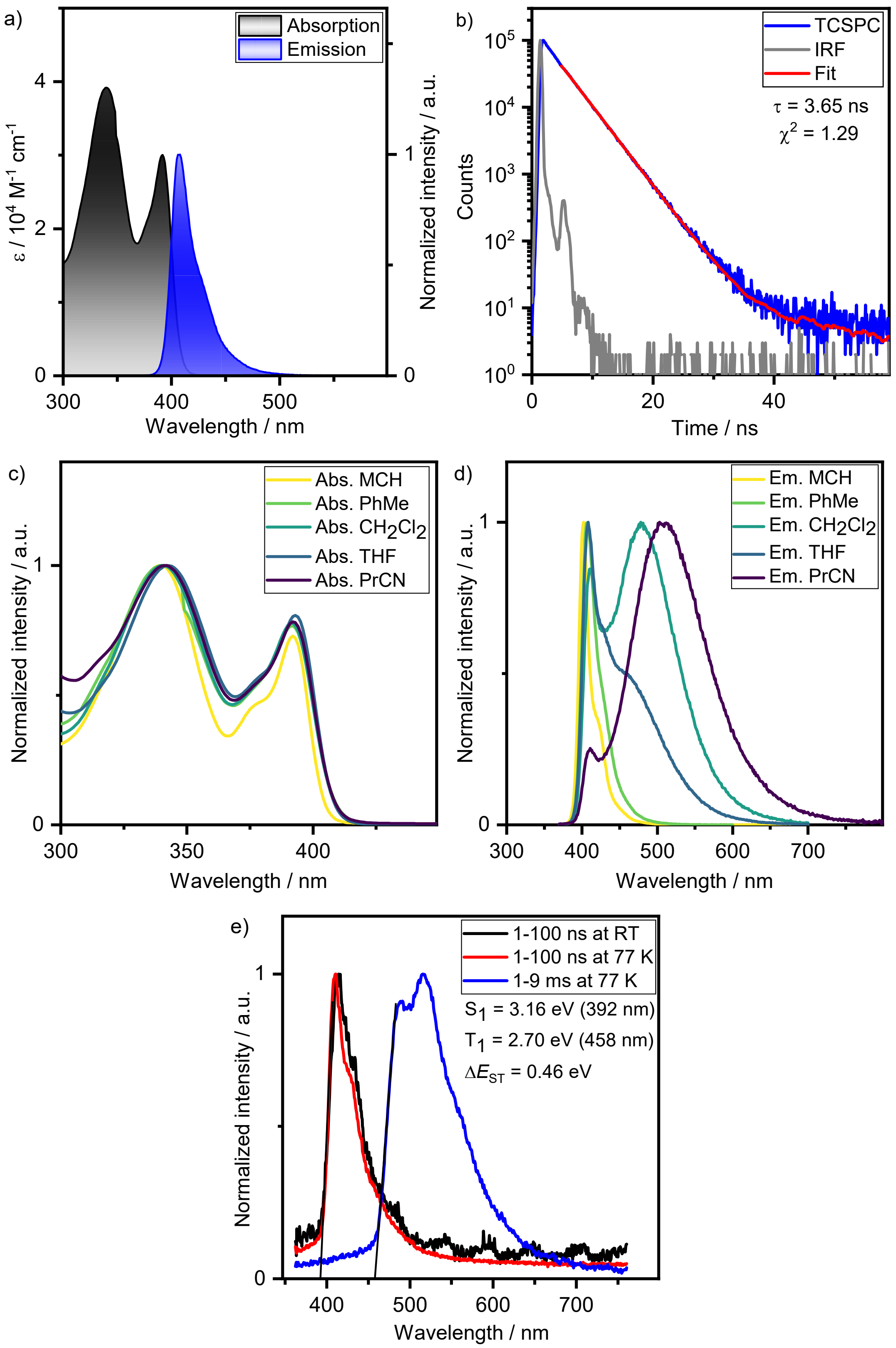


Figure **S44:** Solution-state photophysics of **LC8** in degassed toluene (c = 0.02 mM): a) steady-state absorption (black trace) and emission (blue trace) ($\lambda_{exc}$ = 350 nm); b) luminescence decay (blue trace) with monoexponential fit (red trace) and IRF (grey trace) in the ns range ($\lambda_{exc}$ = 373 nm); c) solvatochromism in solvents of different polarity; d) solvatochromism in solvents of different polarity; e) fluorescence (red trace), phosphorescence (blue trace) and corresponding onsets as $S_1$ and $T_1$ energies at 77K in a frozen toluene matrix ($\lambda_{exc}$ = 343 nm). MCH = methylcyclohexane, PhMe = toluene, PrCN = butyronitrile.

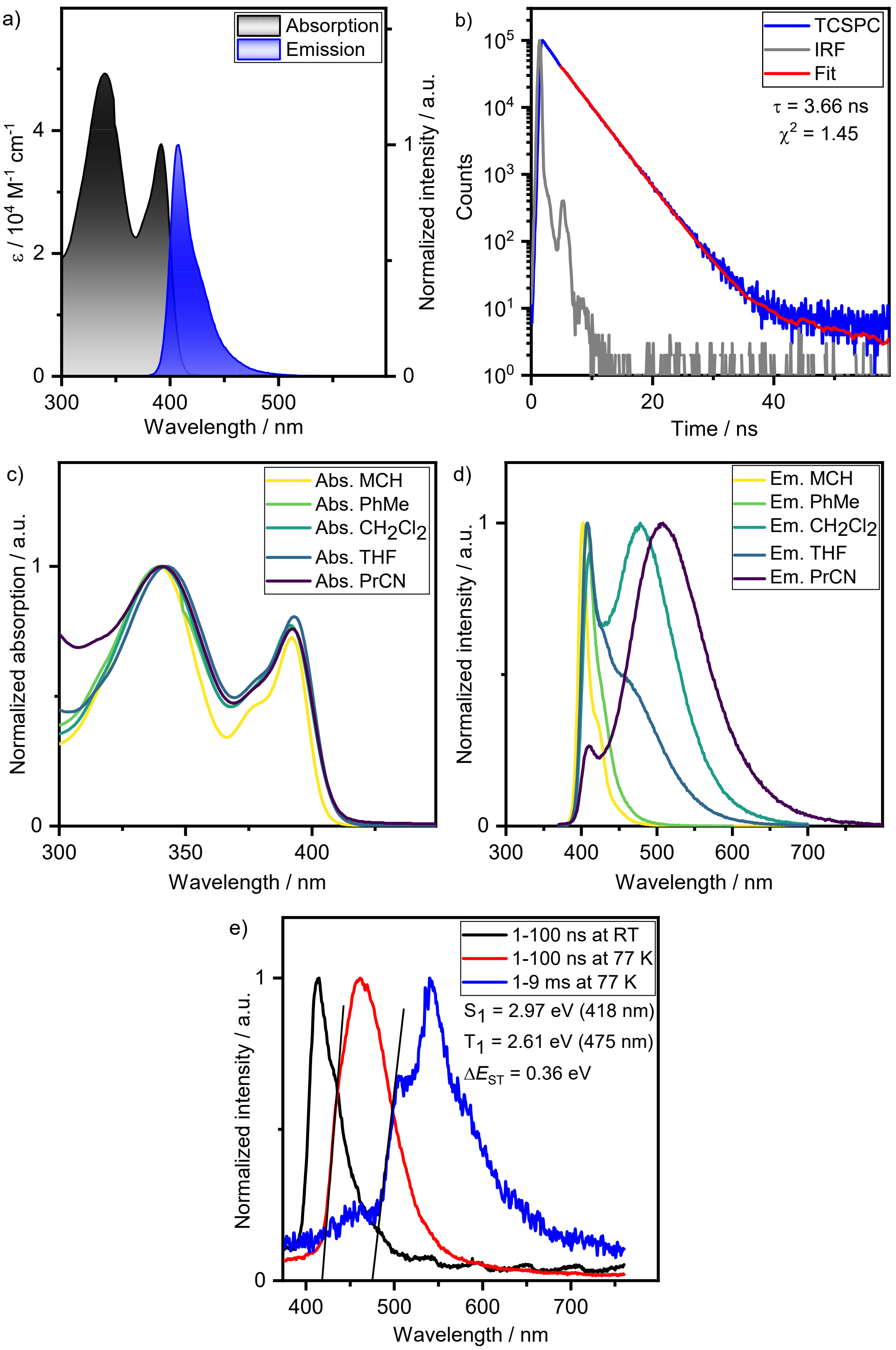


Figure **S45:** Solution-state photophysics of **LC12** in degassed toluene (c = 0.02 mM): a) steady-state absorption (black trace) and emission (blue trace) ($\lambda_{exc}$ = 350 nm); b) luminescence decay (blue trace) with monoexponential fit (red trace) and IRF (grey trace) in the ns range ($\lambda_{exc}$ = 373 nm); c) solvatochromism in solvents of different polarity; d) solvatochromism in solvents of different polarity; e) fluorescence (red trace), phosphorescence (blue trace) and corresponding onsets as $S_1$ and $T_1$ energies at 77K in a frozen toluene matrix ($\lambda_{exc}$ = 343 nm). MCH = methylcyclohexane, PhMe = toluene, PrCN = butyronitrile.

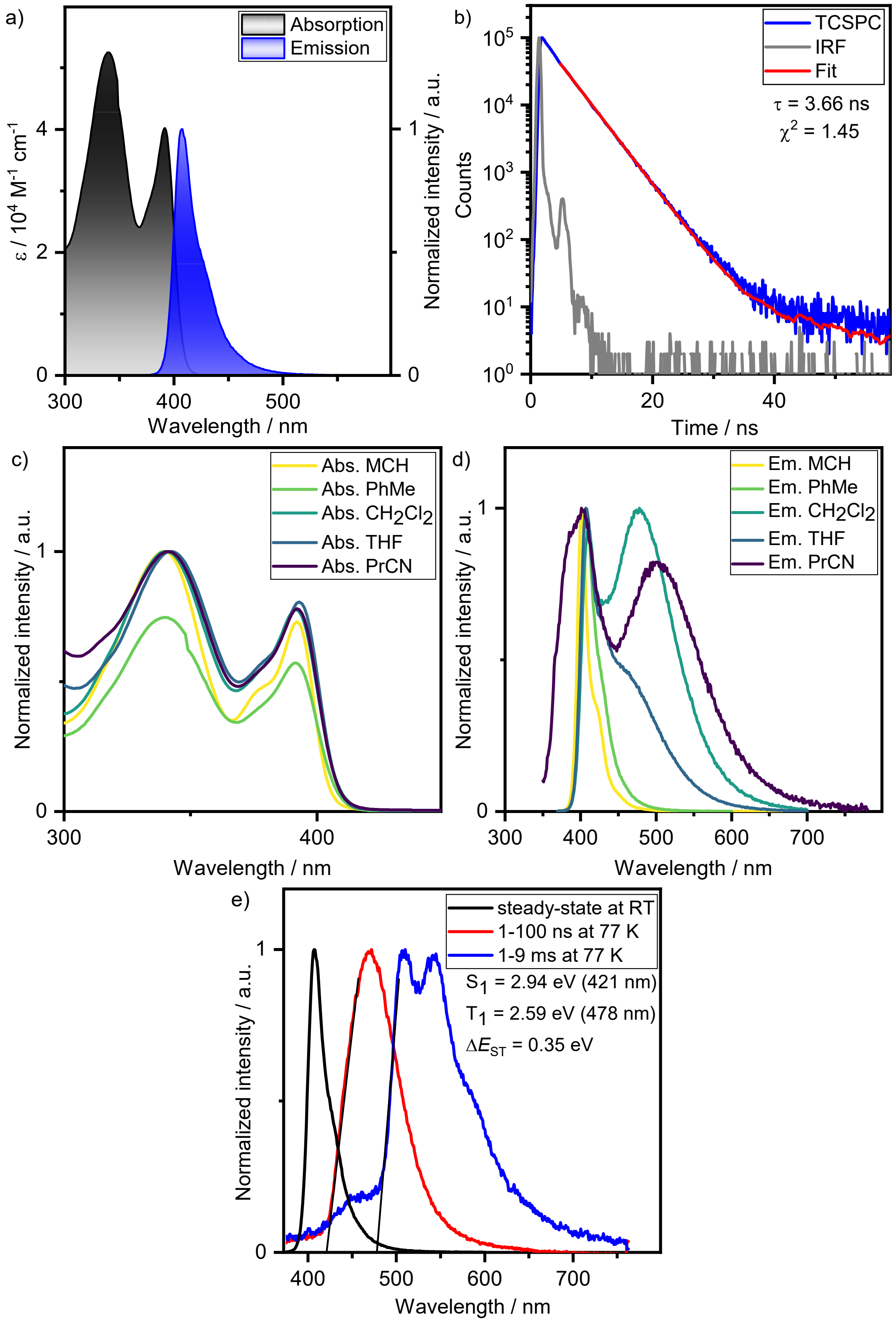


Figure **S46:** Solution-state photophysics of **LC16** in degassed toluene (c = 0.02 mM): a) steady-state absorption (black trace) and emission (blue trace) ($\lambda_{exc}$ = 350 nm); b) luminescence decay (blue trace) with monoexponential fit (red trace) and IRF (grey trace) in the ns range ($\lambda_{exc}$ = 373 nm); c) solvatochromism in solvents of different polarity; d) solvatochromism in solvents of different polarity: the fluorescence spectrum in PrCN showed broadened bands, presumably due to solubility issues; e) fluorescence (red trace), phosphorescence (blue trace) and corresponding onsets as $S_1$ and $T_1$ energies at 77K in a frozen toluene matrix ($\lambda_{exc}$ = 343 nm). MCH = methylcyclohexane, PhMe = toluene, PrCN = butyronitrile.

## 7.3. Photophysical Properties of the LC Series in Neat Films

Table **S9:** Photophysical data of the **LCn** series in neat film (spin-coated, steady-state: $\lambda_{exc}$ = 350 nm, time-resolved: $\lambda_{exc}$ = 379 nm, $\Delta E_{ST}$**:** $\lambda_{exc}$ = 343 nm).

| # | $\lambda_{abs}$ / nm | $\lambda_{PL}$ / nm | FWHM / nm (FWHM / meV) | Stokes shift / meV (stokes shift / cm$^{-1}$) | $\Phi_{PL}$ / %. | $\tau_p$ / ns | $\Delta E_{ST}$ / eV |
|---|---|---|---|---|---|---|---|
| **LC1** | 340<br>397 | 459 | 74 (430.7) | 421.8 (3402.4) | 6 | 6.98 | 0.41 |
| **LC4** | 340<br>392 | 434 | 52 (323.8) | 306.1 (2468.7) | 22 | 6.66 | ~0.34* |
| **LC8** | 341<br>399 | 472 | 70 (403.7) | 480.6 (3876.2) | 19 | 16.62 | 0.37 |
| **LC12** | 341<br>398 | 463 | 88 (500.4) | 437.3 (3527.4) | 19 | 11.87 | 0.44 |
| **LC16** | 341<br>402 | 456 | 80 (462.1) | 365.2 (2945.8) | 20 | 9.95 | 0.40 |

*Please note: **LC4** exhibited prompt emission in the phosphorescence spectrum and the extracted $\Delta E_{ST}$ is thus just a rough estimate.

Table **S10:** Fitting parameters of the triple exponential fits of the TCSPC measurements of the **LCn** series.

| # | $\tau_1$ / ns | $A_1$ / a.u. | $\tau_2$ / ns | $A_2$ / a.u. | $\tau_3$ / ns | $A_3$ / a.u. | $\tau_{avg}$ / ns | $\chi^2$ / a.u. |
|---|---|---|---|---|---|---|---|---|
| **LC1** | 4.25 | 7836.80 | 11.20 | 3962.79 | 38.72 | 144.28 | 6.98 | 1.18 |
| **LC4** | 5.32 | 20097.66 | 10.09 | 6035.15 | 48.93 | 149.98 | 6.66 | 1.17 |
| **LC8** | 6.87 | 14483.32 | 19.30 | 28028.71 | 37.42 | 3187.34 | 16.62 | 1.12 |
| **LC12** | 5.69 | 14920.83 | 14.73 | 18957.85 | 35.83 | 1584.98 | 11.87 | 1.09 |
| **LC16** | 4.98 | 7030.49 | 13.04 | 6551.35 | 36.65 | 404.66 | 9.95 | 1.12 |

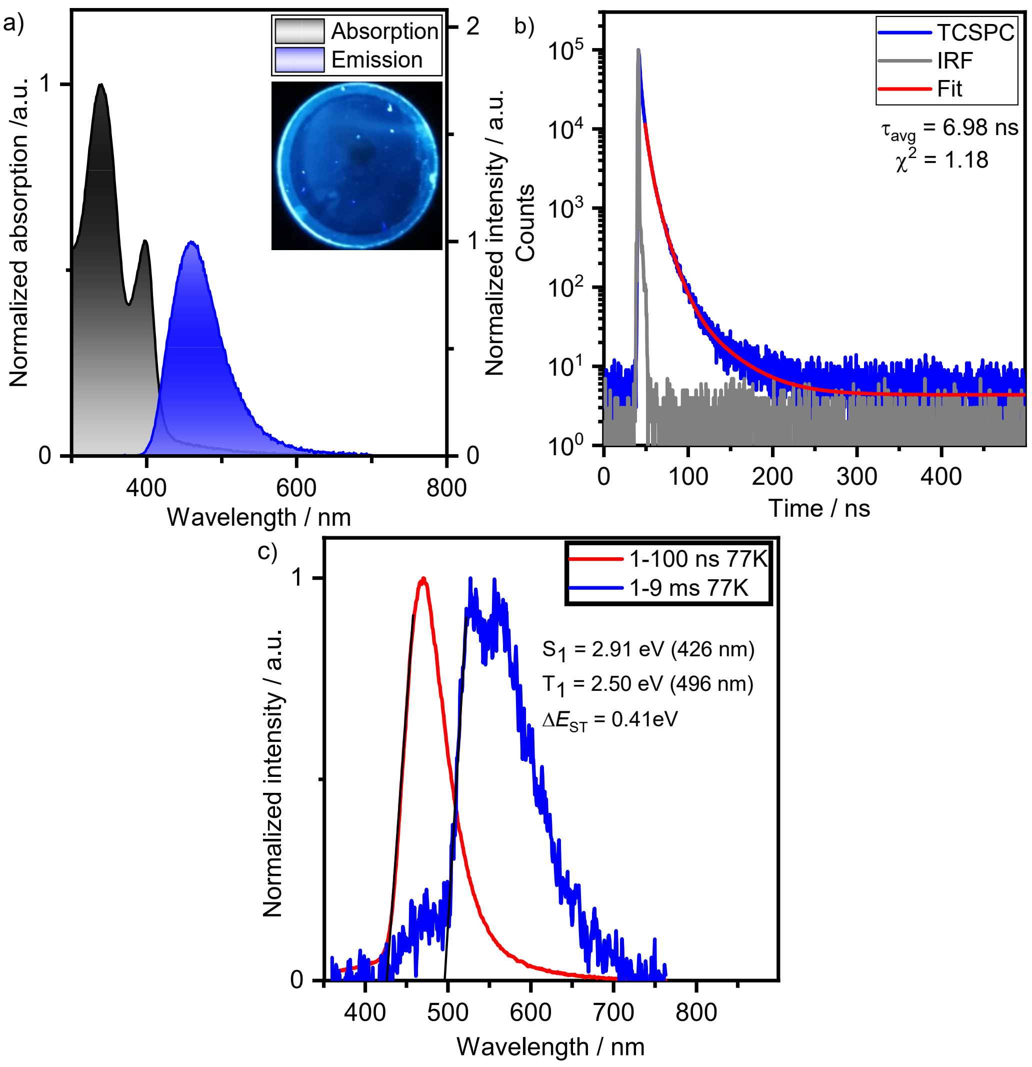


Figure **S47:** Photophysics of **LC1** in a spin coated film: a) steady-state absorption (black trace) and emission (blue trace) ($\lambda_{exc}$ = 350 nm), inset shows the film under UV light; b) luminescence decay (blue trace) with triple exponential fit (red trace) and IRF (grey trace) in the ns range ($\lambda_{exc}$ = 379 nm); c) Fluorescence spectrum (red trace, 1 – 100 ns, 77 K) phosphorescence spectrum (blue trace, 1 – 9 ms, 77 K) and lines (black) for extracting $S_1$ and $T_1$ energies as onsets of fluorescence and phosphorescence spectra, respectively ($\lambda_{exc}$ = 343 nm).

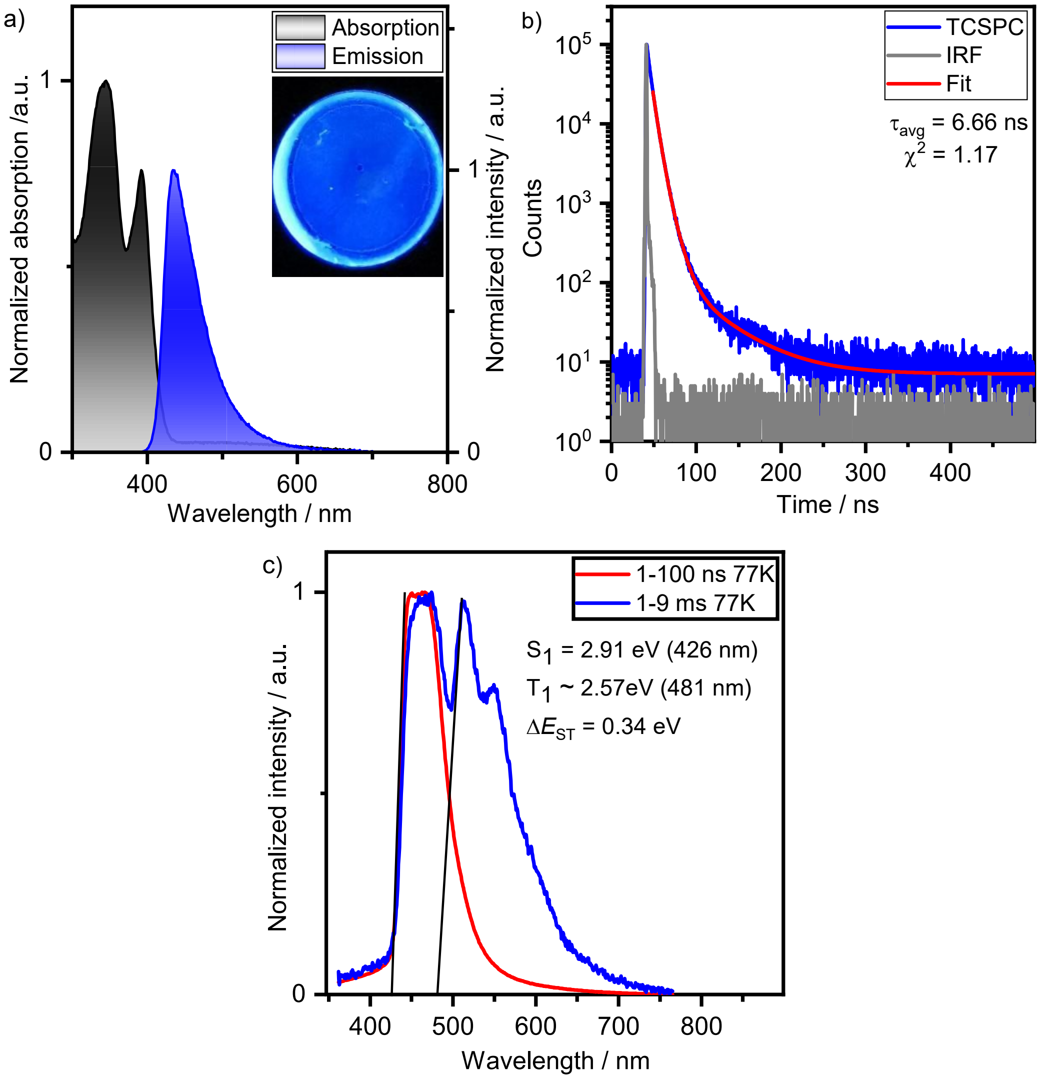


Figure **S48:** Photophysics of **LC4** in a spin coated film: a) steady-state absorption (black trace) and emission (blue trace) ($\lambda_{exc}$ = 350 nm), inset shows the film under UV light; b) luminescence decay (blue trace) with triple exponential fit (red trace) and IRF (grey trace) in the ns range ($\lambda_{exc}$ = 379 nm); c) Fluorescence spectrum (red trace, 1 – 100 ns, 77 K) phosphorescence spectrum (blue trace, 1 – 9 ms, 77 K) and lines (black) for extracting $S_1$ and $T_1$ energies as onsets of fluorescence and phosphorescence spectra, respectively ($\lambda_{exc}$ = 343 nm). Please note: **LC4** exhibited prompt emission in the phosphorescence spectrum, the extracted $T_1$ energy is thus only a rough estimate.

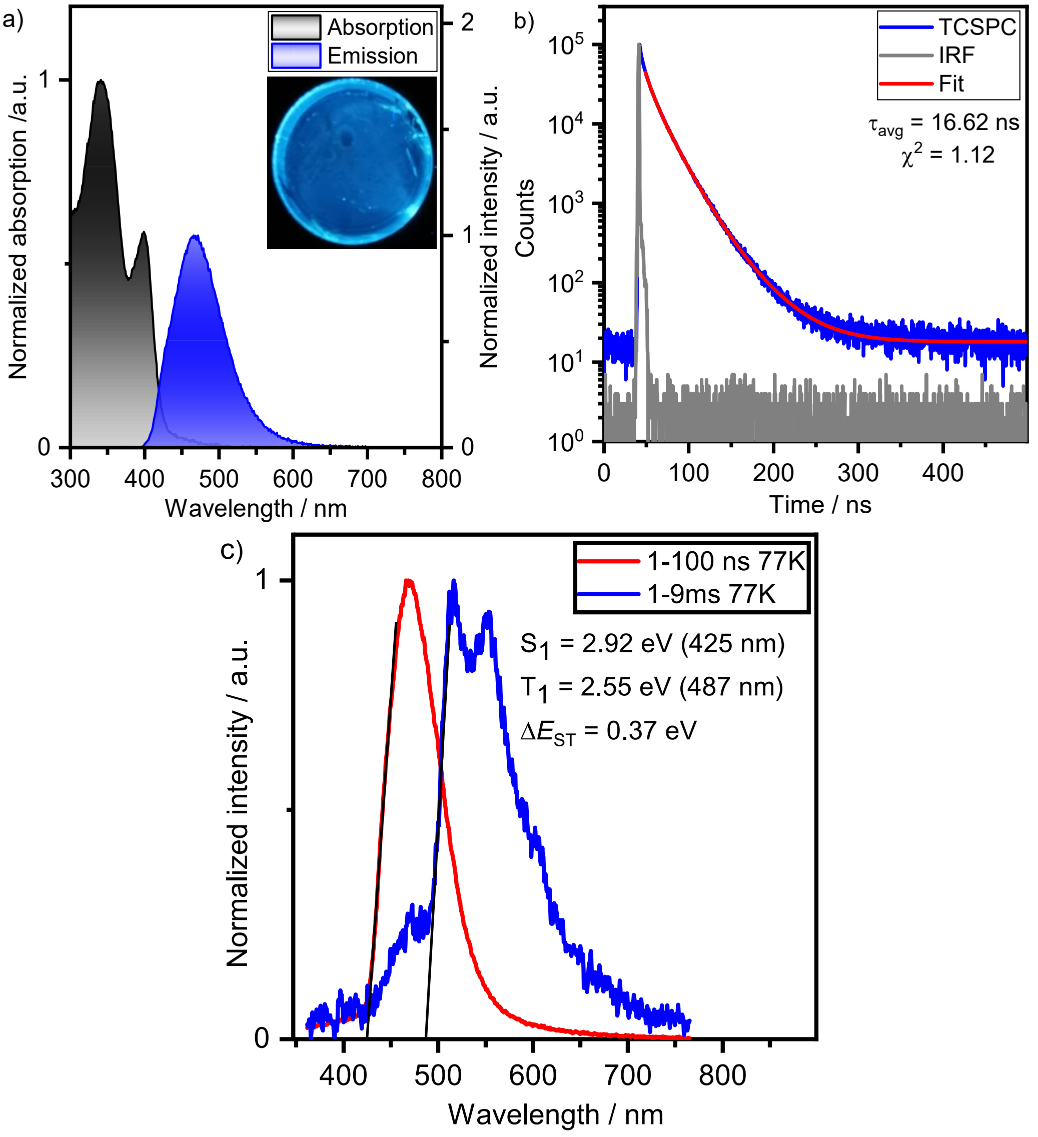


Figure **S49:** Photophysics of **LC8** in a spin coated film: a) steady-state absorption (black trace) and emission (blue trace) ($\lambda_{exc}$ = 350 nm), inset shows the film under UV light; b) luminescence decay (blue trace) with triple exponential fit (red trace) and IRF (grey trace) in the ns range ($\lambda_{exc}$ = 379 nm); c) Fluorescence spectrum (red trace, 1 – 100 ns, 77 K) phosphorescence spectrum (blue trace, 1 – 9 ms, 77 K) and lines (black) for extracting $S_1$ and $T_1$ energies as onsets of fluorescence and phosphorescence spectra, respectively ($\lambda_{exc}$ = 343 nm).

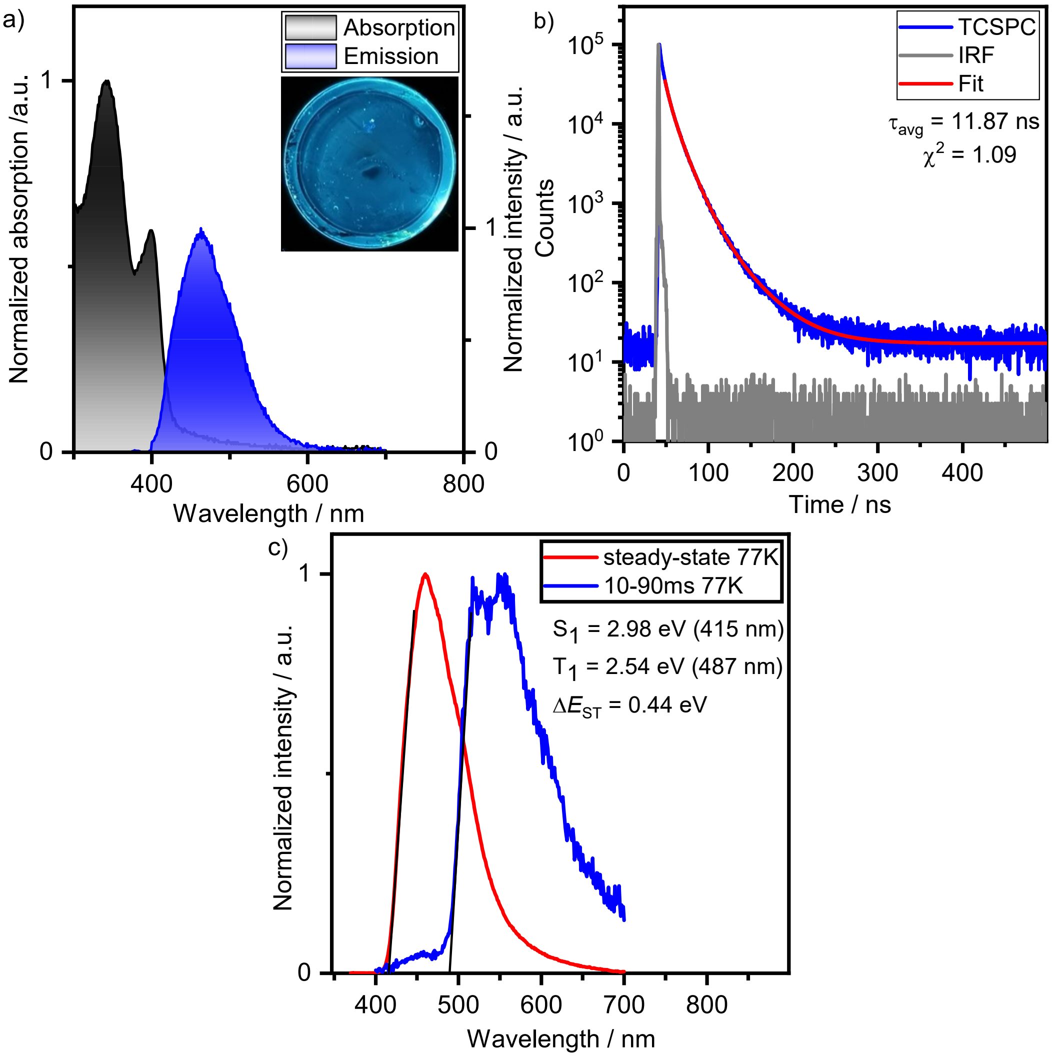


Figure **S50:** Photophysics of **LC12** in a spin coated film: a) steady-state absorption (black trace) and emission (blue trace) ($\lambda_{exc}$ = 350 nm), inset shows the film under UV light; b) luminescence decay (blue trace) with triple exponential fit (red trace) and IRF (grey trace) in the ns range ($\lambda_{exc}$ = 379 nm); c) steady-state spectrum (red trace, 77 K), time-gated spectrum (blue trace, 10 – 90 ms, 77 K) and lines (black) for extracting $S_1$ and $T_1$ energies as onsets of steady-state and time-gated spectra, respectively.

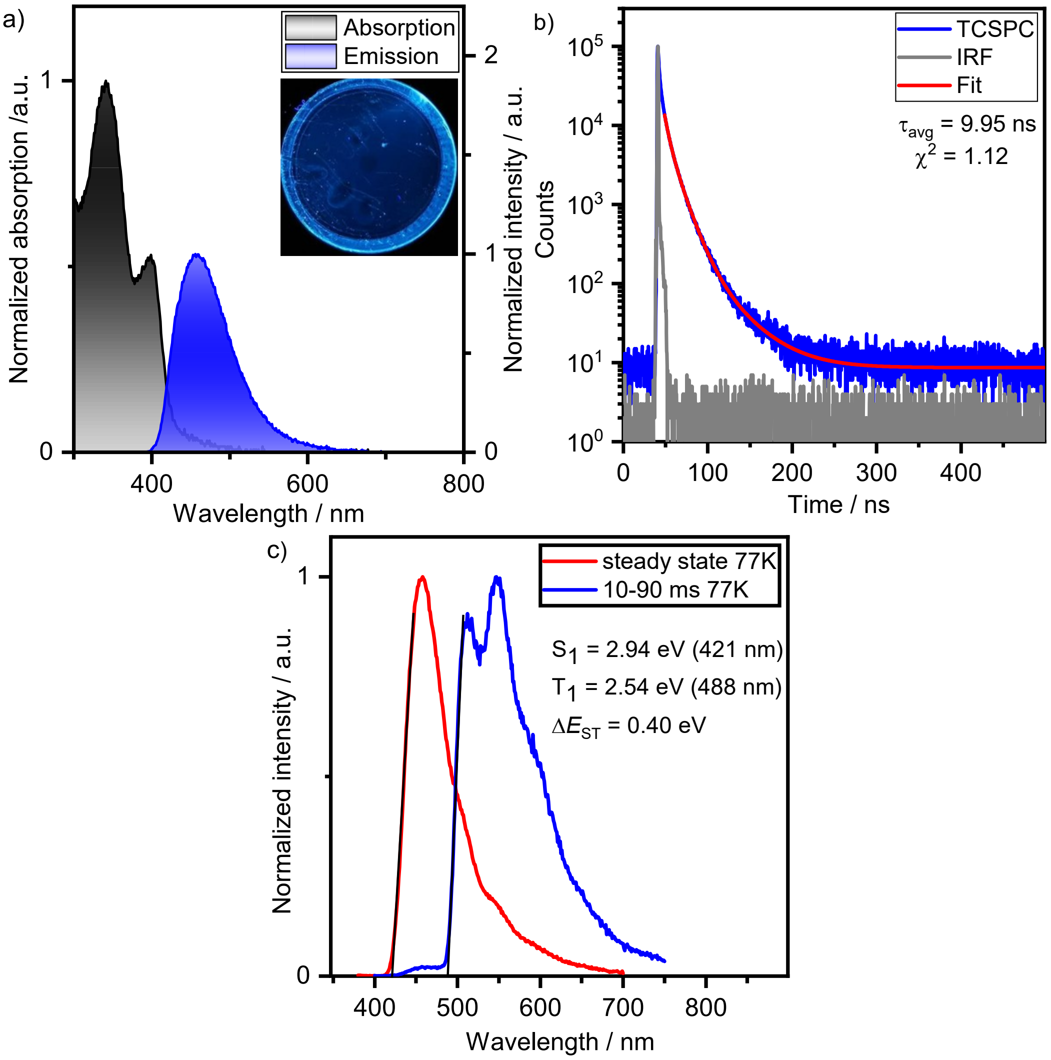


Figure **S51:** Photophysics of **LC16** in a spin coated film: a) steady-state absorption (black trace) and emission (blue trace) ($\lambda_{exc}$ = 350 nm), inset shows the film under UV light; b) luminescence decay (blue trace) with triple exponential fit (red trace) and IRF (grey trace) in the ns range ($\lambda_{exc}$ = 379 nm); c) steady-state spectrum (red trace, 77 K), time-gated spectrum (blue trace, 10 – 90 ms, 77 K) and lines (black) for extracting $S_1$ and $T_1$ energies as onsets of steady-state and time-gated spectra, respectively.

### 7.4. Photophysical Properties of the LC Films doped with BCzBN (1wt%)

Table **S11:** Photophysical data of the **LC** series doped with **BCzBN** (1wt % steady-state: $\lambda_{exc}$ = 350 nm, time-resolved: $\lambda_{exc}$ = 379 nm).

| **BCzBN:** | *c* **BCzBN** (mol%) | $\lambda_{PL}$ / nm | FWHM / nm (FWHM / meV) | Φ / %. | $\tau_p$ / ns | $\tau_d$ / µs |
|---|---|---|---|---|---|---|
| **LC1** | 1.2 | 493 | 47 (232.2) | 33 | 5.51 | -* |
| **LC4** | 1.8 | 487 | 42 (214.6) | 68 | 8.03 | 37.8 |
| **LC8** | 2.5 | 476 | 14 (76.3) | 41 | 8.19 | 108.5 |
| **LC12** | 3.3 | 475 | 15 (81.9) | 44 | 4.04 | 91.5 |
| **LC16** | 4.0 | 479 | 31 (163.9) | 50 | 6.46 | 103.1 |

* no delayed emission detectable.

Table **S12:** Fitting parameters of the triple exponential fits of the TCSPC measurements of the **LC** series doped with **BCzBN** (1wt%).

| **BCzBN:** | $\tau_1$ / ns | $A_1$ / a.u. | $\tau_2$ / ns | $A_2$ / a.u. | $\tau_3$ / ns | $A_3$ / a.u. | $\tau_{avg}$ / ns | $\chi^2$ / a.u. |
|---|---|---|---|---|---|---|---|---|
| **LC1** | 4.17 | 9.24 | 47.54 | 34190.16 | 11253.70 | 94.50 | 5.51 | 1.07 |
| **LC4** | 5.70 | 11.59 | 40.15 | 41822.48 | 23859.02 | 398.78 | 8.03 | 1.11 |
| **LC8** | 5.00 | 14.32 | 33.35 | 24045.67 | 9047.90 | 843.88 | 8.19 | 1.17 |
| **LC12** | 2.48 | 7.14 | 23.18 | 14068.90 | 5378.69 | 282.49 | 4.04 | 1.26 |
| **LC16** | 4.34 | 10.24 | 40.69 | 33140.12 | 15368.30 | 360.53 | 6.46 | 1.08 |

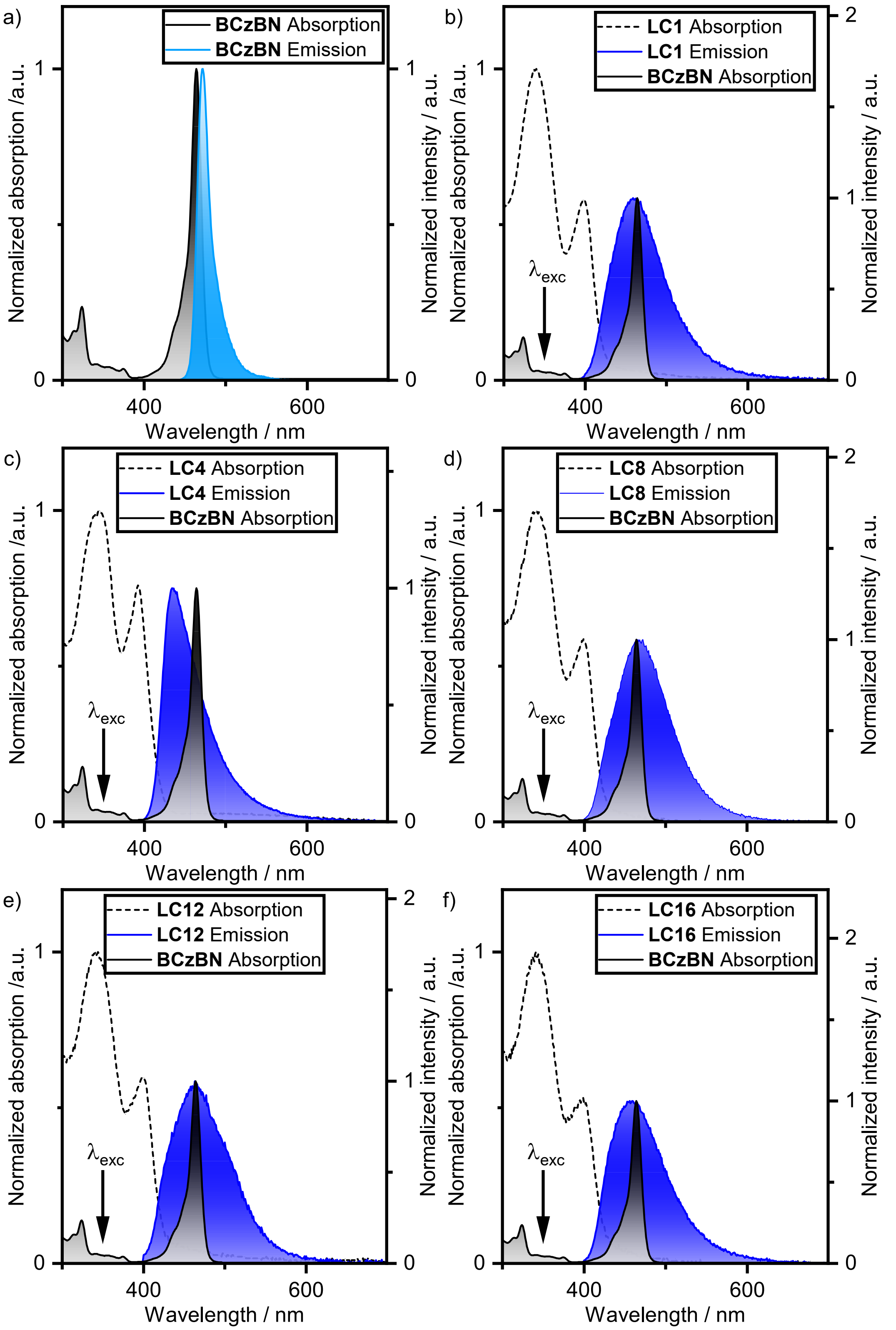


Figure **S52:** a) Absorption (black trace) and emission (blue trace) of **BCzBN** in methylcyclohexane (c = 0.02 mM, $\lambda_{exc}$ = 420 nm); overlap of the absorption (black traces, 0.02 mM in methylcyclohexane) of **BCzBN** with the emission spectra of the respective **LCn** derivative in a neat film, absorption of the respective **LCn** derivative in a neat film shown for comparison (dashed traces): b) for **LC1**, c) for **LC4**, d) for **LC8**, e) for **LC12**, for **LC16**. Please note: The comparison of **LCn** and **BCzBN** absorption is of qualitative nature and not corrected for the concentration of **BCzBN** in the doped films. $\lambda_{exc}$ is the excitation of the doped films.

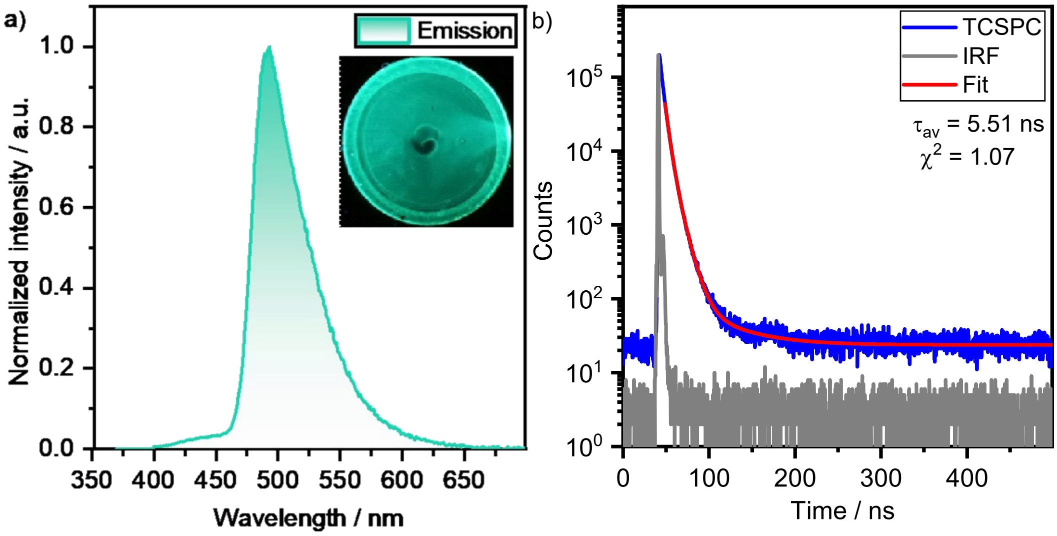


Figure **S53:** Photophysics of **LC1** doped with **BCzBN** (1 wt%) in a spin coated film: a) steady-state emission (blue trace, $\lambda_{exc}$ = 350 nm), inset shows the film under UV light; b) luminescence decay (blue trace) with triple exponential fit (red trace) and IRF (grey trace) in the ns range ($\lambda_{exc}$ = 379 nm).

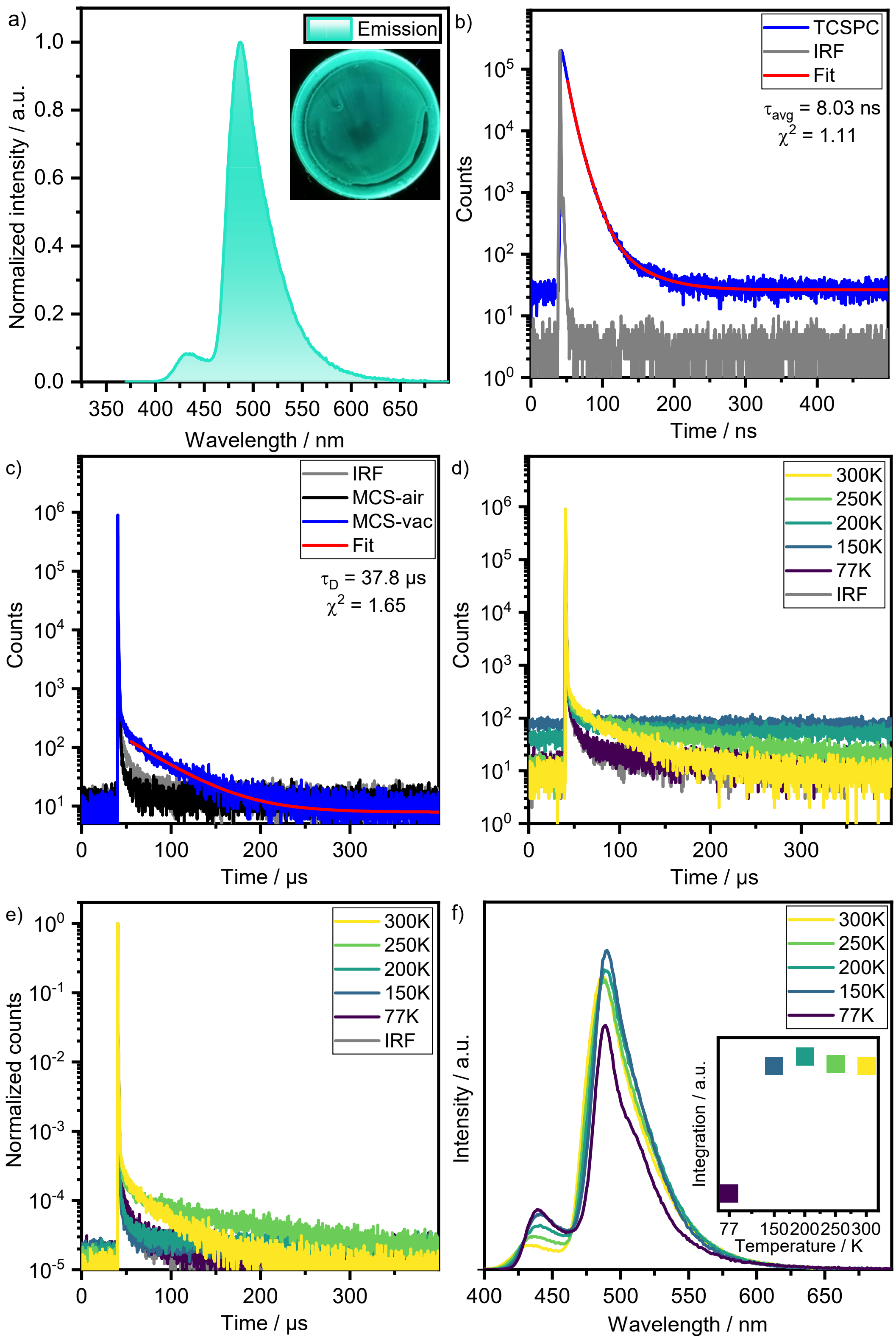


Figure **S54** Photophysics of **LC4** doped with **BCzBN** (1 wt%) in a spin coated film: a) steady-state emission ($\lambda_{exc}$ = 350 nm), inset shows the film under UV light; b) luminescence decay (blue trace) with triple exponential fit (red trace) and IRF (grey trace) in the ns range ($\lambda_{exc}$ = 379 nm); c) luminescence decay under air (black trace) and vacuum (blue trace) and IRF (grey trace) in the µs range ($\lambda_{exc}$ = 379 nm); d) temperature dependent luminescence decay in the µs range ($\lambda_{exc}$ = 379 nm); e) Normalized MCS data; f) temperature dependent steady-state photoluminescence ($\lambda_{exc}$ = 350 nm).

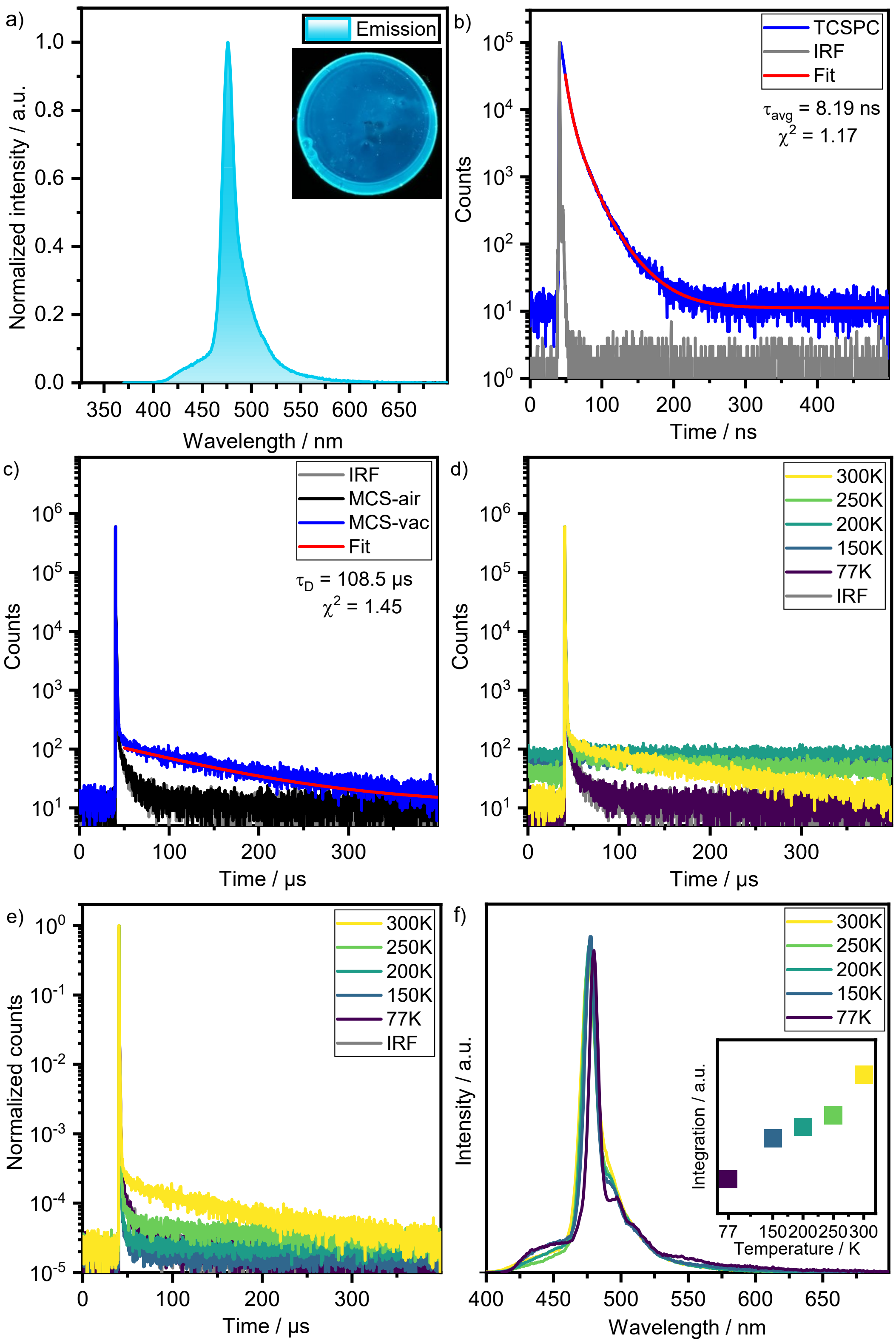


Figure **S55:** Photophysics of **LC8** doped with **BCzBN** (1 wt%) in a spin coated film: a) steady-state emission ($\lambda_{exc} = 350$ nm), inset shows the film under UV light; b) luminescence decay (blue trace) with triple exponential fit (red trace) and IRF (grey trace) in the ns range ($\lambda_{exc} = 379$ nm); c) luminescence decay under air (black trace) and vacuum (blue trace) and IRF (grey trace) in the μs range ($\lambda_{exc} = 379$ nm); d) temperature dependent luminescence decay in the μs range ($\lambda_{exc} = 379$ nm); e) Normalized MCS data; f) temperature dependent steady-state photoluminescence ($\lambda_{exc} = 350$ nm).

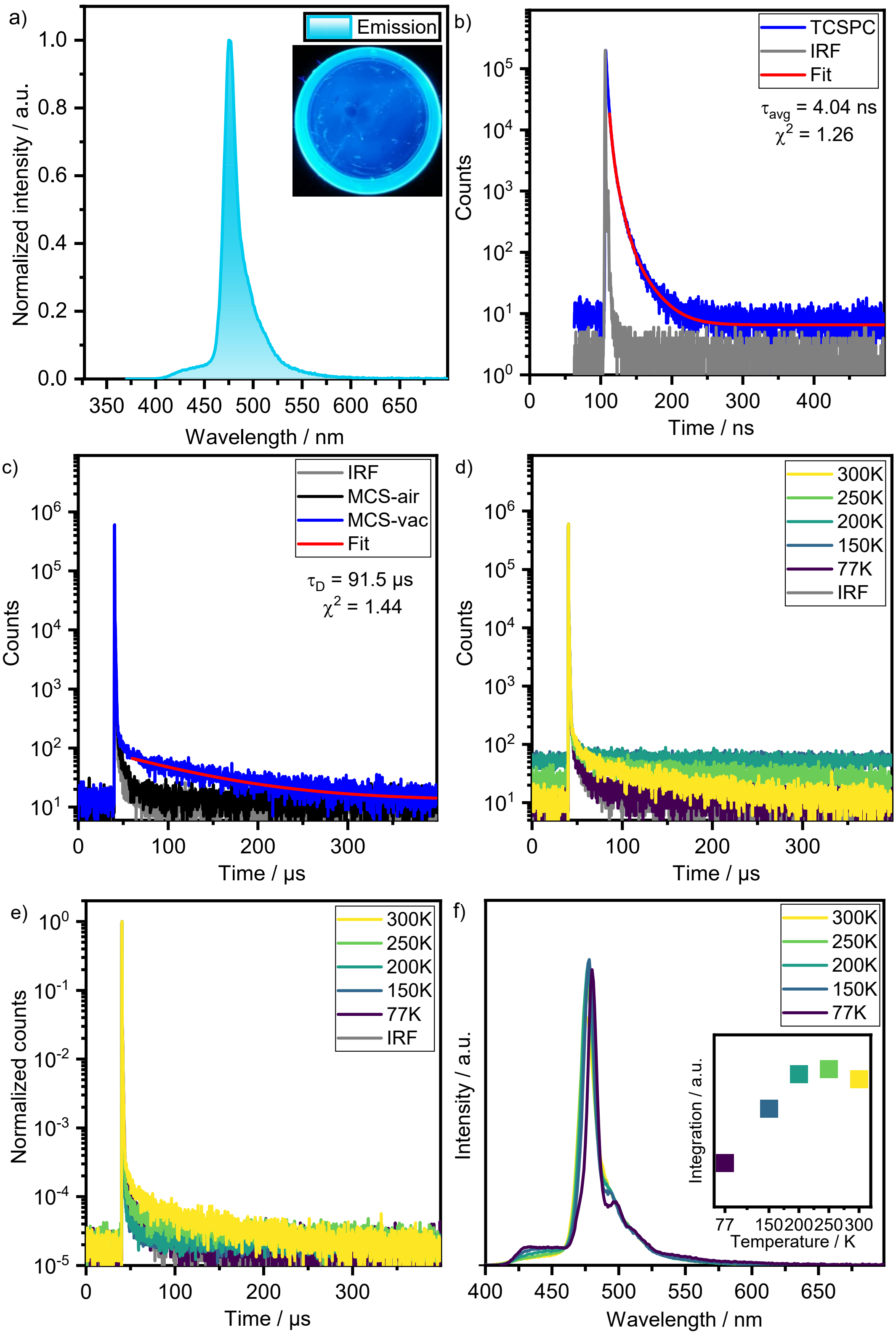


Figure **S56:** Photophysics of **LC12** doped with **BCzBN** (1 wt%) in a spin coated film: a) steady-state emission ($\lambda_{exc}$ = 350 nm), inset shows the film under UV light; b) luminescence decay (blue trace) with triple exponential fit (red trace) and IRF (grey trace) in the ns range ($\lambda_{exc}$ = 379 nm); c) luminescence decay under air (black trace) and vacuum (blue trace) and IRF (grey trace) in the µs range ($\lambda_{exc}$ = 379 nm); d) temperature dependent luminescence decay in the µs range ($\lambda_{exc}$ = 379 nm); e) Normalized MCS data; f) temperature dependent steady-state photoluminescence ($\lambda_{exc}$ = 350 nm).

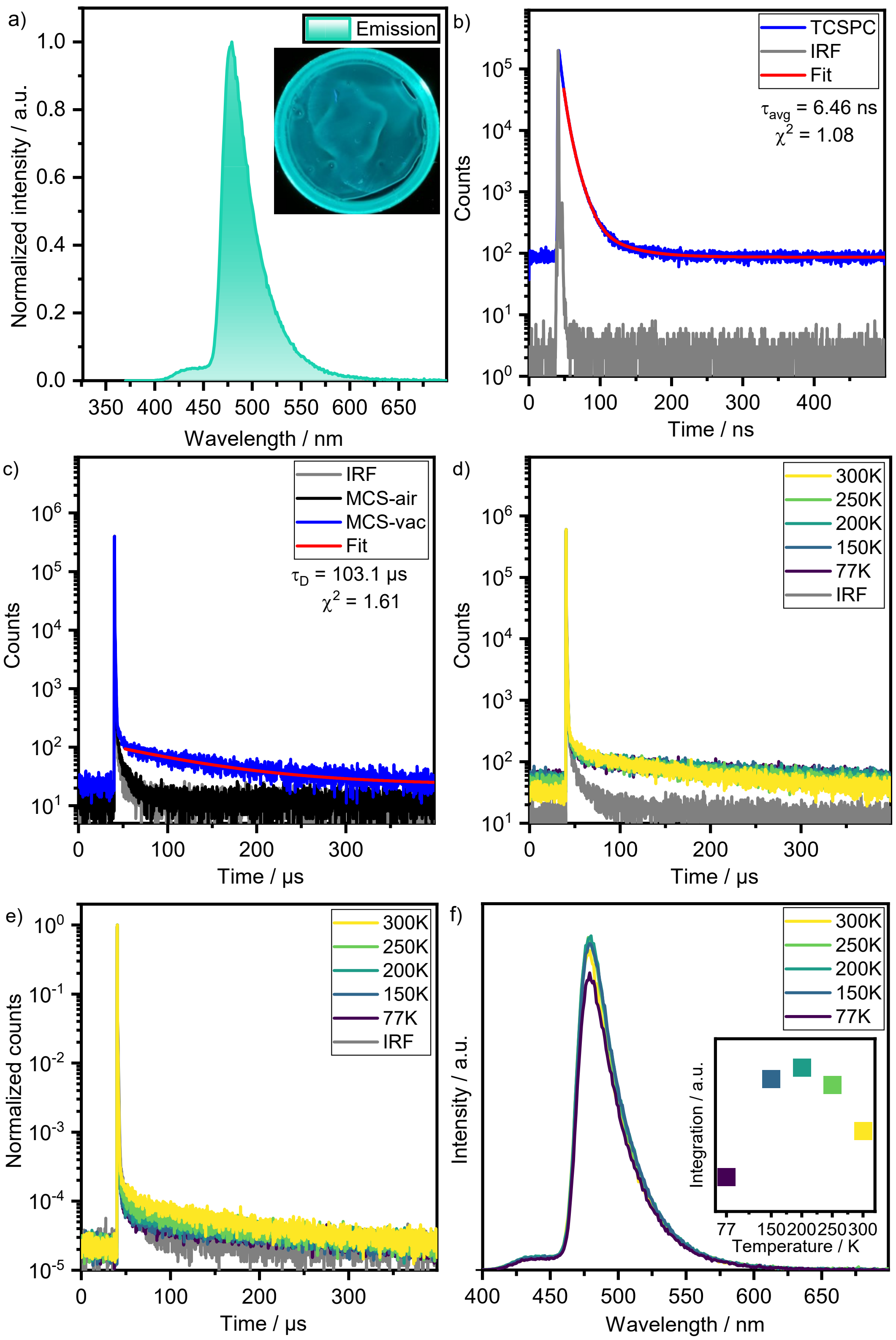


Figure **S57:** Photophysics of **LC16** doped with **BCzBN** (1 wt%) in a spin coated film: a) steady-state emission ($\lambda_{exc}$ = 350 nm), inset shows the film under UV light; b) luminescence decay (blue trace) with triple exponential fit (red trace) and IRF (grey trace) in the ns range ($\lambda_{exc}$ = 379 nm); c) luminescence decay under air (black trace) and vacuum (blue trace) and IRF (grey trace) in the µs range ($\lambda_{exc}$ = 379 nm); d) temperature dependent luminescence decay in the µs range ($\lambda_{exc}$ = 379 nm) ; e) Normalized MCS data; f) temperature dependent steady-state photoluminescence ($\lambda_{exc}$ = 350 nm).

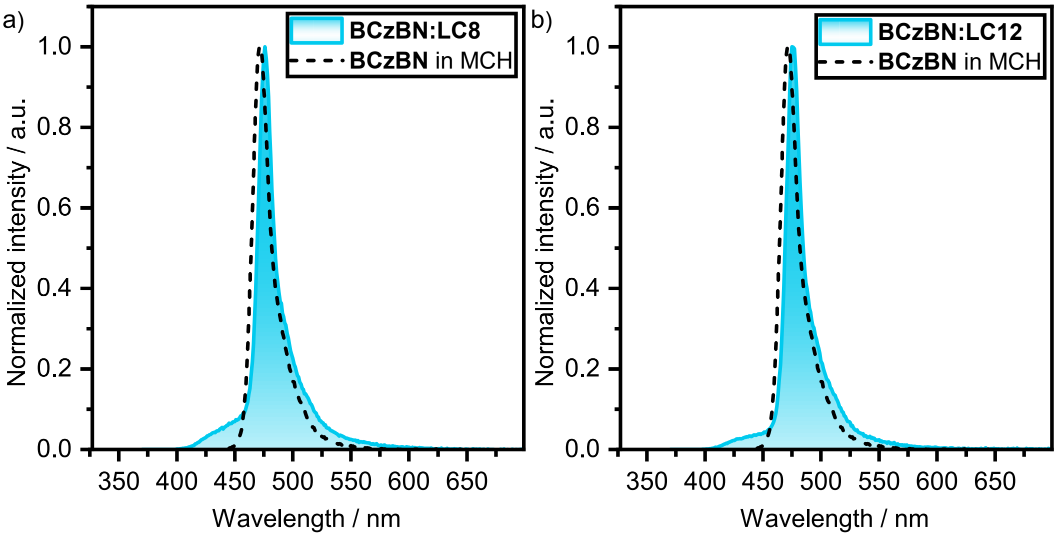


Figure **S58:** Comparison of the emission of **BCzBN** in MCH (methylcyclohexane, 0.02 mM, black dashed traces, $\lambda_{exc}$ = 420 nm) with the emission of a) **BCzBN** in **LC8** and b) **BCzBN** in **LC12**.

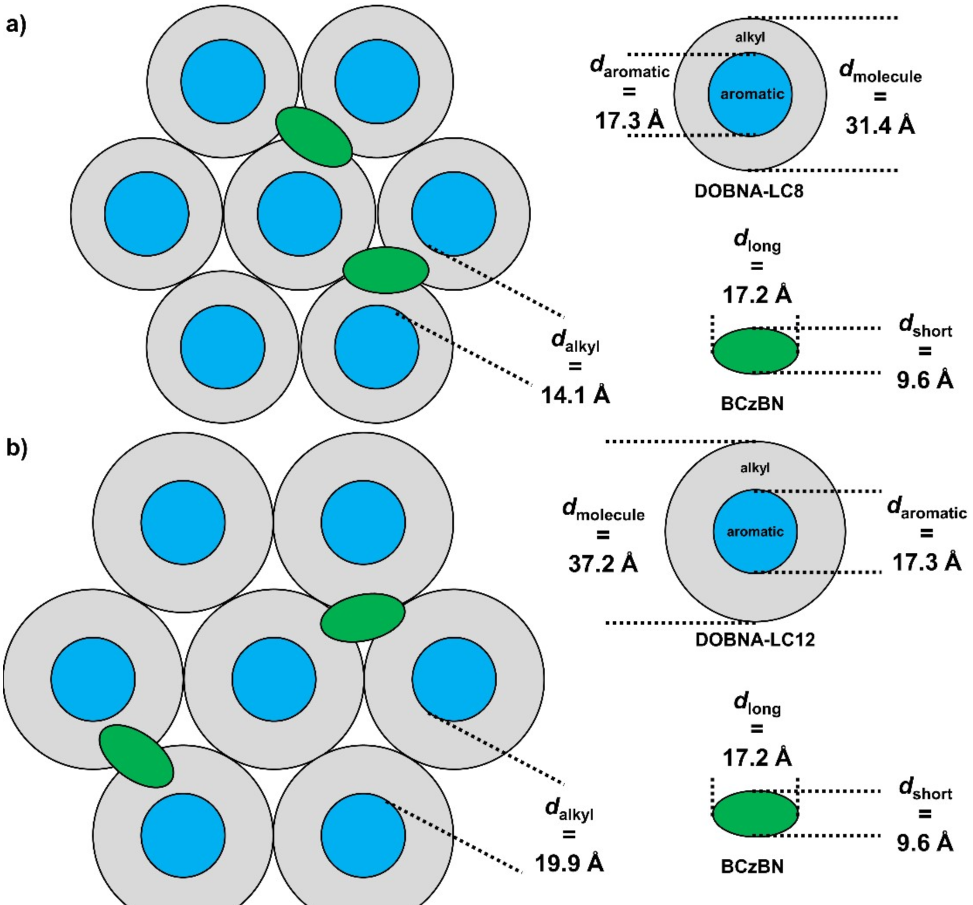


Figure **S59:** Schematic packing models of the **BCzBN:LCn** films derived from SAXS data ($d_{molecule} = a$), DFT calculations ($d_{aromatic}$), $d_{long}$ and $d_{short}$ from single crystal Xray structure of BCzBN (CCDC: 2032785)[37] and photophysical data (assembly of the emitter in the alkyl domains) for: a) **LC8:BCzBN**, b) **LC12:BCzBN**.

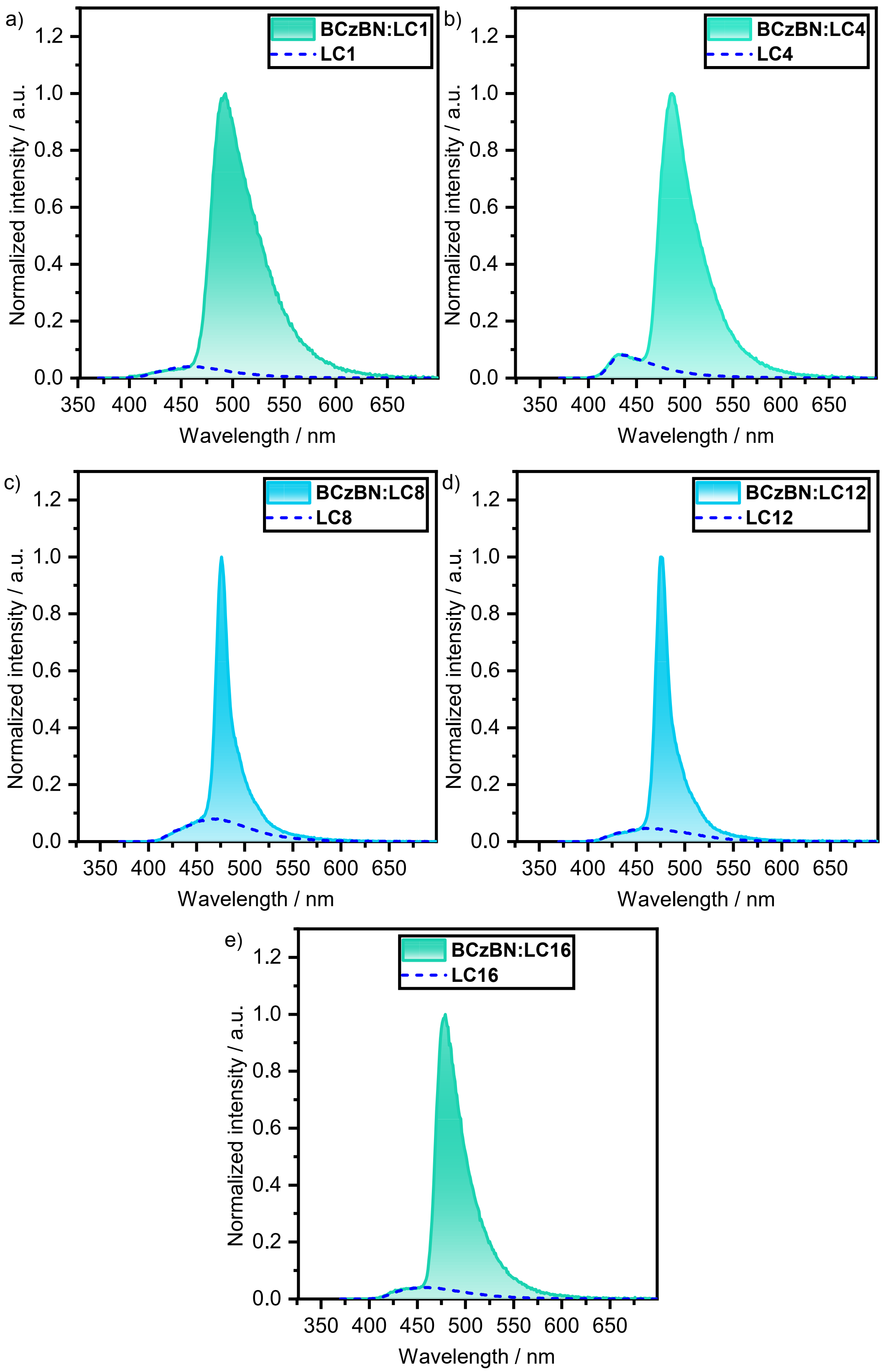


**Figure S60:** Emission spectra of the **BCzBN:LCn** mixtures with scaled emission spectra of the respective **LCn** host in spin coated films ($\lambda_{exc}$ = 350 nm) for: a) **BCzBN:LC1**; b) **BCzBN:LC4**; c) **BCzBN:LC8**; d) **BCzBN:LC12**; e) **BCzBN:LC16**.

**Table S13:** Integrals of the whole spectra of **BCzBN:LCn** ($I_{DA}$), integrals of the scaled spectra of the respective host **LCn** ($I_D$, Figure S60) and observed FRET efficiency ($E_{PR,}$ $E_{PR} = (I_{DA} - I_D)/I_{DA}$).[37]

| BCzBN: | LC-1 | LC4 | LC8 | LC12 | LC16 |
|---|---|---|---|---|---|
| $I_{DA}$ / a.u. | 57.09 | 53.6 | 26.82 | 26.02 | 41.91 |
| $I_D$ / a.u. | 3.47 | 5.15 | 6.93 | 4.29 | 3.58 |
| observed FRET efficiency ($E_{PR}$) / % | 95 | 90 | 74 | 84 | 92 |

# 8. Literature


1 Gaussian 16, Revision C.01, M. J. Frisch, G. W. Trucks, H. B. Schlegel, G. E. Scuseria, M. A. Robb, J. R. Cheeseman, G. Scalmani, V. Barone, G. A. Petersson, H. Nakatsuji, X. Li, M. Caricato, A. V. Marenich, J. Bloino, B. G. Janesko, R. Gomperts, B. Mennucci, H. P. Hratchian, J. V. Ortiz, A. F. Izmaylov, J. L. Sonnenberg, D. Williams-Young, F. Ding, F. Lipparini, F. Egidi, J. Goings, B. Peng, A. Petrone, T. Henderson, D. Ranasinghe, V. G. Zakrzewski, J. Gao, N. Rega, G. Zheng, W. Liang, M. Hada, M. Ehara, K. Toyota, R. Fukuda, J. Hasegawa, M. Ishida, T. Nakajima, Y. Honda, O. Kitao, H. Nakai, T. Vreven, K. Throssell, J. A. Montgomery, Jr., J. E. Peralta, F. Ogliaro, M. J. Bearpark, J. J. Heyd, E. N. Brothers, K. N. Kudin, V. N. Staroverov, T. A. Keith, R. Kobayashi, J. Normand, K. Raghavachari, A. P. Rendell, J. C. Burant, S. S. Iyengar, J. Tomasi, M. Cossi, J. M. Millam, M. Klene, C. Adamo, R. Cammi, J. W. Ochterski, R. L. Martin, K. Morokuma, O. Farkas, J. B. Foresman, and D. J. Fox, Gaussian, Inc., Wallingford CT, **2016**.

2 F. Neese, *WIREs Comput. Mol. Sci.,* 2022, **12**, e1606.

3 F. Neese, *WIREs Comput. Mol. Sci.,* 2012, **2**, 73–78.

4 C. Adamo and V. Barone, *J. Chem. Phys.,* 1999, **110**, 6158–6170.

5 G. A. Petersson, T. G. Tensfeldt, J. A. Montgomery, *J. Chem. Phys.,* 1991, **94**, 6091–6101.

6 M. Casanova-Páez and L. Goerigk, *J. Chem. Theory Comput.*, 2021, **17**, 5165–5186.

7 R. A. Kendall, T. H. Dunning and R. J. Harrison, *J. Chem. Phys.,* 1992, **96**, 6796–6806.

8 O. S. Lee and E. Zysman-Colman, Digichem (version 6) InSilico Computing, St Andrews, Scotland, 2024.

9 O. S. Lee, M. Gather and E. Zysman-Colman, ChemRxiv. (Preprint), 2024, 10.26434/chemrxiv-2024-v9vrf.

10 N. M. O'boyle, A. L. Tenderholt and K. M. Langner, *J. Comput. Chem.*, 2008, **29**, 839–845.

11 W. Humphrey, A. Dalke and K. Schulten, *J. Mol. Graphics*, 1996, **14**, 33–38.

12 J. Stone, Master's Thesis, Computer Science Department, University of Missouri-Rolla, 1998.

13 J. D. Hunter, *Comput. Sci. Eng.*, 2007, **9**, 90–95.

14 N. M. O'Boyle, M. Banck, C. A. James, C. Morley, T. Vandermeersch and G. R. Hutchison, *J. Cheminform.*, 2011, **3**, 33.

15 N. M. O'Boyle and G. R. Hutchison, *Chem. Cent. J.*, 2008, **2**, 24.

16 X. Gao, S. Bai, D. Fazzi, T. Niehaus, M. Barbatti and W. Thiel, *J. Chem. Theory Comput.*, 2017, **13**, 515–524.

17 D. B. G. Williams and M. Lawton, *J. Org. Chem.*, 2010, **75**, 8351–8354.

18 S. Hitosugi, D. Tanimoto, W. Nakanishi and H. Isobe, *Chem. Lett.*, 2012, **41**, 972–973.

19 G. R. Fulmer, A. J. M. Miller, N. H. Sherden, H. E. Gottlieb, A. Nudelman, B. M. Stoltz, J. E. Bercaw and K. I. Goldberg, *Organometallics*, 2010, **29**, 2176–2179.

20 A. Sillen and Y. Engelborghs, *Photochem. Photobiol.*, 1998, **67**, 475–486.

21 G. A. Crosby and J. N. Demas, *J. Phys. Chem.*, 1971, **75**, 991–1024.

22 A. M. Brouwer, *Pure and Applied Chemistry*, 2011, **83**, 2213–2228.

23 N. G. Connelly and W. E. Geiger, *Chem. Rev.*, 1996, **96**, 877–910.

24 C. M. Cardona, W. Li, A. E. Kaifer, D. Stockdale and G. C. Bazan, *Adv. Mater.*, 2011, **23**, 2367–2371.

25 T. Yasuda, T. Shimizu, F. Liu, G. Ungar and T. Kato, *J. Am. Chem. Soc.*, 2011, **133**, 13437–13444.

26 A. Kapf, H. Eslahi, M. Blanke, M. Saccone, M. Giese and M. Albrecht, *New J. Chem.*, 2019, **43**, 6361–6371.

27 H. Maeda, Y. Haketa and T. Nakanishi, *J. Am. Chem. Soc.*, 2007, **129**, 13661–13674.

28 F. Leroux, T. U. Hutschenreuter, C. Charrière, R. Scopelliti and R. W. Hartmann, *HCA*, 2003, **86**, 2671–2686.

29 J. W. Coe, M. C. Wirtz, C. G. Bashore and J. Candler, *Org. Lett.*, 2004, **6**, 1589–1592.

30 B. Adelizzi, P. Chidchob, N. Tanaka, B. A. G. Lamers, S. C. J. Meskers, S. Ogi, A. R. A. Palmans, S. Yamaguchi and E. W. Meijer, *J. Am. Chem. Soc.*, 2020, **142**, 16681–16689.

31 T. Wöhrle, S. J. Beardsworth, C. Schilling, A. Baro, F. Giesselmann and S. Laschat, *Soft Matter*, 2016, **12**, 3730–3736.

32 S. Sugiura, W. Matsuda, W. Zhang, S. Seki, N. Yasuda and H. Maeda, *J. Org. Chem.*, 2019, **84**, 8886–8898.

33 Y. Haketa, S. Sakamoto, K. Chigusa, T. Nakanishi and H. Maeda, *J. Org. Chem.*, 2011, **76**, 5177–5184.

34 S. Xu, Q. Yang, Y. Zhang, H. Li, Q. Xue, G. Xie, M. Gu, J. Jin, L. Huang and R. Chen, *Chinese Chemical Letters*, 2021, **32**, 1372–1376.

35 H. Hirai, K. Nakajima, S. Nakatsuka, K. Shiren, J. Ni, S. Nomura, T. Ikuta and T. Hatakeyama, *Angew. Chem. Int. Ed.*, 2015, **54**, 13581–13585.
36 C. Reichardt and T. Welton, *Solvents and Solvent Effects in Organic Chemistry*, Wiley, 2010.
37 J. J. McCann, U. B. Choi, L. Zheng, K. Weninger and M. E. Bowen, *Biophysical Journal*, 2010, **99**, 961–970.
38 M. Yang, I. S. Park and T. Yasuda, *J. Am. Chem. Soc.*, 2020, **142**, 19468–19472.
